# Stochastic Forces in Microbial Community Assembly: Founding Community Size Governs Divergent Ecological Trajectories

**DOI:** 10.1111/ele.70388

**Published:** 2026-05-03

**Authors:** Ibuki Hayashi, Martina Sánchez‐Pinillos, Hirokazu Toju

**Affiliations:** ^1^ Laboratory of Ecosystems and Coevolution, Graduate School of Biostudies Kyoto University Kyoto Japan; ^2^ Centre for Forest Research Université du Québec à Montréal Montreal Quebec Canada; ^3^ Center for Living Systems Information Science (CeLiSIS), Graduate School of Biostudies Kyoto University Kyoto Japan

**Keywords:** alternative stable states, alternative transient states, community assembly, ecological dynamic regimes, invasion ecology, microbiome dynamics, multistability, positive feedback, priority effects, stability landscapes

## Abstract

Biological community dynamics arise from both deterministic and stochastic processes. While species' responses to environmental factors define attractors of community structure, stochasticity, particularly during early assembly, can redirect ecological trajectories. However, quantifying such roles of stochasticity in community assembly has remained challenging. We tracked community assembly in two multi‐replicated experimental systems, each with four levels of founding community size, analysing > 3000 samples across four time points. Stronger initial stochasticity led to greater divergence of both population‐ and community‐level consequences. Strikingly, conspicuous differentiation into alternative trajectories of community assembly occurred when the absolute number of founding prokaryotic cells was less than the order of 10^4^. Thus, quantitative differences in stochasticity produced qualitative differences in community fate. These results demonstrate that early stochastic events can have enduring impacts on ecological dynamics. Deeper quantitative insights into stochasticity will reorganise our views on biological invasions, agroecosystem microbiome management, and therapeutics of human‐associated microbiomes.

## Graphical abstract

The processes driving community assembly are broadly categorised into deterministic and stochastic ones. With a multi‐replicated experimental design, we quantified the extent to which stochasticity at initial stages of community assembly could drive divergence into alternative ecological trajectories.

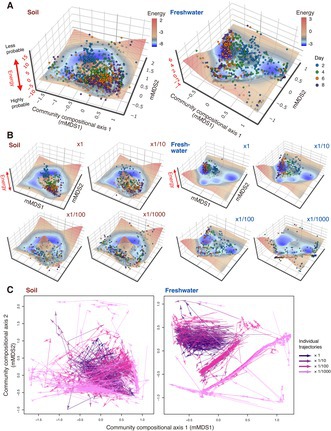

## 1 Introduction

**FIGURE 1:**
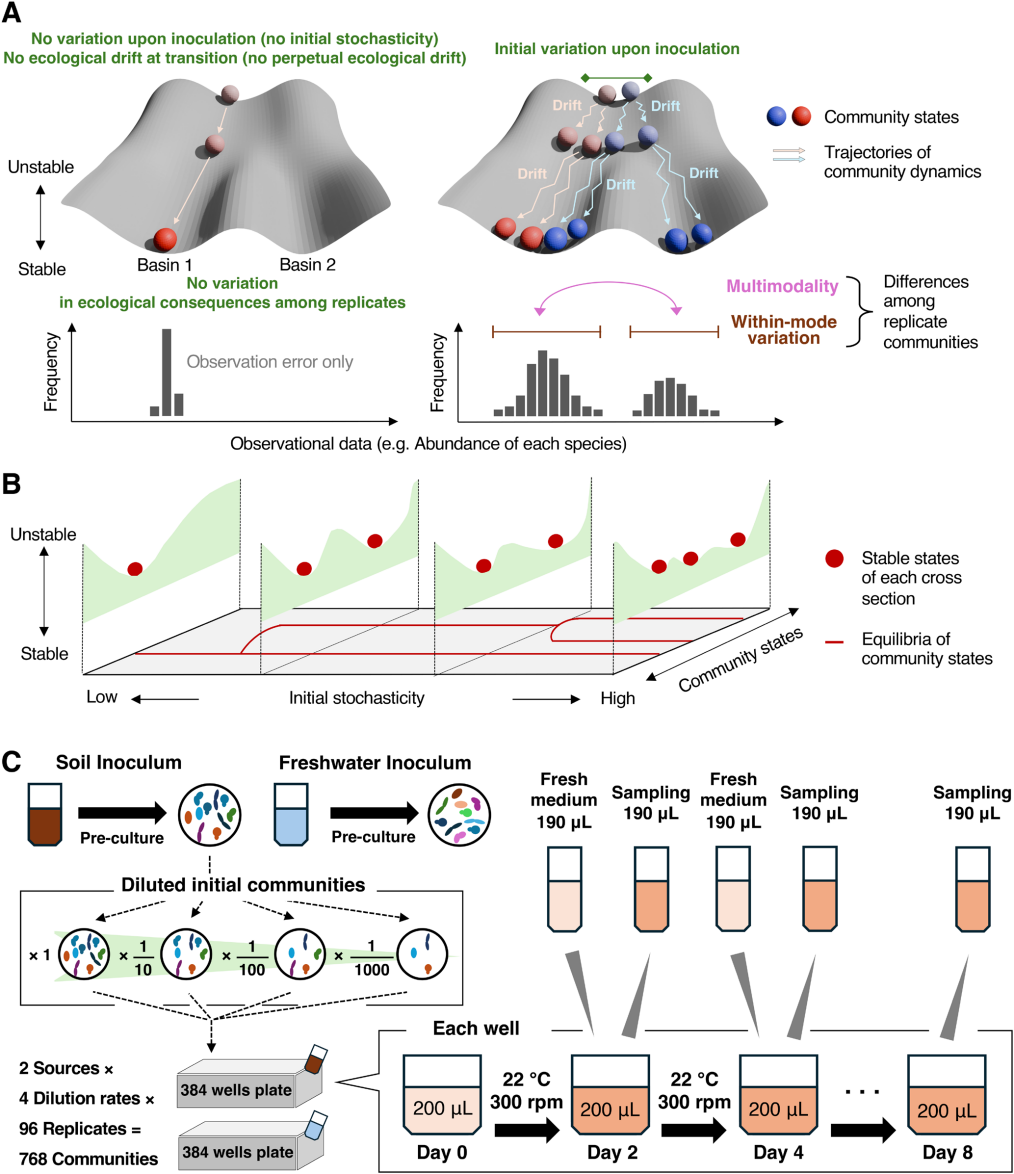
Theoretical concepts and experimental design. (A) Interplay of deterministic and stochastic processes (Framework 1). In fully deterministic ecological processes, divergence into alternative community states does not occur (left panel). In real ecosystems, divergence into alternative “basins” of attraction would occur on a stability landscape depending on stochasticity at the onset of a community (initial stochasticity) and ecological drift during community assembly. (right panel). Among‐replicate variation in community structure can be quantified based on a histogram as detailed in Figures 2 and 3. (B) Initial stochasticity as a hyperparameter (Framework 2). In this alternative conceptual framework, initial stochasticity organises the architecture of the stability landscape. For example, bifurcations or multifurcations (i.e., changes in the number of attractors) may occur along an axis of initial stochasticity. (C) Experimental design. Each microbiome sample derived from a soil or freshwater ecosystem was precultured with cycloheximide at room temperature for 2 days to remove eukaryotes. Each inoculum source was diluted across four dilution series, yielding eight inoculum settings (2 sources × 4 dilution series). For each inoculum setting, 96 replicate experimental communities were constructed. A fraction of the culture fluid was sampled every 2 days (48 h), and an equivalent volume of fresh medium was added to the continuous culture system throughout the eight‐day experiment (8 inoculum settings × 96 replicates × 4 time points = 3072 community samples).

The processes driving community assembly are broadly categorised into deterministic and stochastic ones (Clark 2009; Chase and Myers 2011; Vellend et al. 2014; Zhou and Ning 2017; Shoemaker et al. 2020). Community structure is shaped by deterministic “selection” processes, including environmental filtering (Kraft et al. 2015) and species interactions (HilleRisLambers et al. 2012). On a “ball‐and‐cup” analogy of community assembly, the deterministic processes are represented by the architecture of a landscape depicting the relationship between community‐scale structure and stability (Figure 1A, left) (Beisner et al. 2003; Ridolfi et al. 2007; Van Meerbeek et al. 2021). Along with the selection processes, stochastic fluctuations in community compositions operate continuously throughout community dynamics (Arani et al. 2021; Hastings et al. 2021). For example, ecological drift, which results from random demographic events occurring irrespective of species' traits, acts persistently and adds complexities to temporal changes in community structure (Hubbell 2005; Vellend et al. 2014; Gilbert and Levine 2017). On a stability landscape, such stochastic processes are illustrated as continuous fluctuations of community states, which may eventually diverge into different basins representing alternative stable states (Beisner et al. 2003; Fukami 2015) (Figure 1A, right). Thus, fundamental insights into community assembly are available only when we understand the interplay between deterministic processes (changes in landscape architecture) and stochastic processes (random demographic fluctuations) (Jeraldo et al. 2012; Vellend et al. 2014; Måren et al. 2018; Ning et al. 2020).

Whereas possible destinations of community dynamics (system's attractors) per se are set by deterministic processes, stochastic processes can determine the direction of community assembly (i.e., divergence into alternative basins). In particular, during early and unstable phases of community assembly, slight differences in community compositions can have great impacts on the subsequent community dynamics, driving divergence into alternative basins of attraction (Zhou et al. 2013; Lerch et al. 2023; Hayashi et al. 2024). In the absence of stochasticity during the initial stages of assembly, repeated observations under identical external conditions are expected to yield identical communities (Figure 1A, left). Importantly, the extent to which communities diverge into alternative states depends strongly on the level of initial stochasticity, provided that species invasions do not occur at later stages (Scheffer and Carpenter 2003; Lopes et al. 2024). Theoretical studies have shown that the population size of each founding species limits the range of subsequent community dynamics, thereby regulating divergence into alternative basins (Abbott and Nolting 2017; Kalirad and Sommer 2024). Nonetheless, we still have limited empirical knowledge of how stochasticity during early assembly generates “multi‐stable” patterns at the community scale.

In comprehensively understanding community dynamics on stability landscapes, quantitative experimental insights into stochastic processes are essential (Shoemaker et al. 2020). In principle, quantifying stochasticity requires repeated observations. In other words, the roles of stochasticity on community assembly are evaluated only when we gain bird's‐eye views of large numbers of replicate communities (Szabo et al. 2022; Pascual‐García et al. 2025). As discussed above, the existence of multiple basins does not guarantee that alternative community states will actually be observed (Spagnolo et al. 2003; Mankin et al. 2006). Thus, it is crucial to examine how the level of divergence into alternative basins depends on stochasticity at community foundation events, based on multi‐replicated observations of community temporal dynamics. Experimentally, this research design raises the question of how initial stochasticity can be systematically controlled and quantified. When communities are founded by sampling individuals from a common species pool, stochastic variation in each species' abundance (the number of individuals of each species) arises from demographic sampling (i.e., initial stochasticity). Under binomial or multinomial sampling assumptions, these stochastic effects decrease as the size of the founding community (total number of individuals) increases, resulting in reduced among‐replicate variability in community structure. Accordingly, in experimental systems with many replicates, initial stochasticity can be controlled by manipulating the total number of individuals in founding communities while keeping deterministic processes effectively constant (Siqueira et al. 2020). Microbial communities are ideal for systematically designed studies because they allow highly replicated experiments that capture diverse ecological outcomes (e.g., differentiation into multiple basins) and, their short generation times relative to macro‐organisms enable experiments to be completed within short time frames (Estrela et al. 2022; Hayashi et al. 2024). Consequently, highly replicated microbiome experiments with systematically varied founding community sizes will provide a powerful framework for quantifying the diversification of community dynamics.

We herein constructed a highly replicated experimental system to examine how initial stochasticity, which was systematically controlled by varying the number of microbial cells in founding (source) communities, could determine divergence into alternative community states. We established laboratory microbiomes using two types of source communities (soil and freshwater), each of which was subjected to a dilution series with four levels. For each dilution level, 96 replicate communities were tracked across four time points. The community compositional analysis of > 3000 samples allowed us to examine how initial stochasticity level could influence community assembly. We hypothesised that lowering inoculum concentrations would increase the level of initial stochasticity, which, in turn, could amplify variation in community dynamics. We tested this hypothesis through three complementary analyses allowing the quantification of among‐sample divergence in community structure. First, using the frequency distribution of each species' abundance across the 96 replicates at each time point, we extracted two indices, “multimodality” and “within‐mode variation”, as measures of discrete dynamics and within‐basin fluctuations, respectively (Figure 1A, right), and examined their relationships with initial stochasticity. Second, we extended this quantitative approach to the community level by calculating the same two indices for whole‐community states. Third, by applying “ecological dynamic regime” analysis for time‐series community data (Sánchez‐Pinillos et al. 2023), we quantitatively examined how founding community size could determine the diversity of community dynamic trajectories. Overall, the systematic experimental design with a high number of replicate communities provides quantitative insights into the mechanisms underlying the realisation of alternative ecological fates.

## 2 Material and Methods

### 2.1 Concepts and Assumptions

In considering how initial stochasticity level influences community stability, there can be alternative conceptual frameworks for interpreting observed patterns. One is to assume a fixed stability landscape structure on which community dynamics are driven by stochastic processes such as ecological drift, which cause random demographic fluctuations (Framework 1). In this assumption, the landscape structure remains unchanged with initial stochasticity, and temporal dynamics vary among replicate communities depending on starting positions (founding community compositions) and the subsequent fluctuations on the fixed landscape (Figure 1A, right) (Chase 2003; Vellend et al. 2014; Song 2025). By contrast, deterministic processes themselves may vary depending on initial stochasticity level (Courchamp et al. 1999; Stephens et al. 1999). Under this alternative interpretation, observed community consequences are explained by assuming that the topography of the stability landscape varies across different levels of initial stochasticity (Framework 2) (Abreu et al. 2019; Venturelli et al. 2018). In this framework, initial stochasticity is treated as a “hyperparameter” that controls the architecture of the stability landscape, such as the number and configuration of attractors (Figure 1B). Note that we consider communities maintained under constant environmental conditions: hence, conceptual frameworks that explicitly assume environmental variability (Chesson 2017) do not apply to the present experimental system.

In this study, we interpret community assembly data mainly based on Framework 1. We quantify among‐replicate variation in community compositions using a statistical distribution model. We then examine whether initial stochasticity level influences variation in population‐ and community‐level consequences. Subsequently, to interpret the data based on Framework 2, we apply recently proposed statistical platforms assuming the dynamic nature of stability landscapes and calculating statistical metrics of ecological trajectories (Suzuki et al. 2021; Sánchez‐Pinillos et al. 2023; Masuda et al. 2025).

### 2.2 Continuous Culture of Microbiomes

We used two types of field‐collected microbiomes as source communities of the experiment. One microbiome was sampled from the surface soil (0–10 cm in depth) of a warm‐temperate forest (34.972° N, 135.958° E) on January 30, 2023, and the other was from the surface water of a nearby freshwater pond (34.974° N, 135.967° E). Each source was filtered and pre‐cultured in PBS buffer containing cycloheximide for 48 h to eliminate non‐prokaryotic organisms.

To make a gradient of founding community size, the base inoculum sample (×1) was serially diluted for three steps, making ×1/10, ×1/100, and ×1/1000 inocula for each of the soil and freshwater sources (Figure 1C). We introduced each of the eight inoculum microbiomes (2 sources × 4 dilution rates) into M9 medium containing minimal inorganic additives and three carbon sources (Table S1), with 96 replicates each. Communities were cultured with serial dilution transfers and sampled every two days for eight days. In total, 3072 microbiome samples (8 inoculum settings × 96 replicates × 4 time points) were collected for the subsequent statistical analyses (see Supporting Information for detailed sampling and culturing procedures).

### 2.3 DNA Metabarcoding and Bioinformatics

The community compositions of the microbiome samples were analysed based on 16S rRNA amplicon sequencing with a previously reported protocol of molecular and bioinformatic procedures (Hayashi et al. 2024). We obtained (i) amplicon sequence variants (ASVs), representing biologically meaningful unique sequences resolved at single‐nucleotide resolution; (ii) operational taxonomic units (OTUs), generated by clustering ASVs at 99% sequence similarity; and (iii) genus‐ or family‐level taxonomic assignments for the ASVs (Supporting Information).

To reveal the community compositions and size of the source communities (soil and freshwater), we used a quantitative amplicon sequencing platform for estimating the copy numbers of 16S rRNA genes in sample aliquots (Fujita et al. 2023a) (Figure S1). The 16S rRNA gene copy estimates were then converted into the estimated number of prokaryotic cells in the inoculum suspensions. In this calculation, 16S rRNA gene tandem repeat number per genome was inferred for each taxon using the RasperGade16S database v0.0.1.0 (Gao and Wu 2023).

### 2.4 Estimation of Initial Stochasticity

**FIGURE 2:**
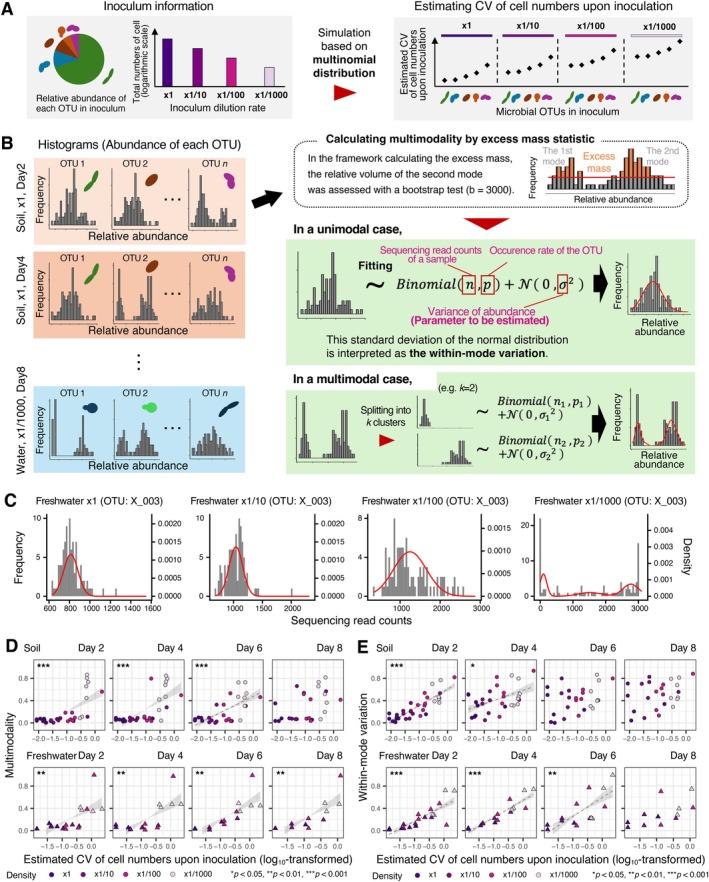
Initial stochasticity and divergence in OTU abundance among replicate communities. (A) Estimation of the coefficient of variation of cell numbers upon inoculation. When the relative abundance of each OTU and the total number of cells in the inoculum are known, the number of cells of each OTU introduced at inoculation follows a multinomial distribution, enabling the calculation of its coefficient of variation (CV) across simulated replicates. The CV of cell numbers is expected to increase with decreasing relative abundance and decreasing total numbers of inoculated cells. (B) Multimodality and within‐mode variation of OTU abundance. For each microbial OTU, the degree to which its abundance histogram across replicates exhibited multiple peaks was assessed as multimodality. For each peak identified in the multimodality analysis, among‐replicate variation was modelled as a binomial process with additive standard normal noise (mixture model). Within‐mode variation is then evaluated as the standard deviation of the standard normal distribution. (C) Examples of abundance histograms. For an OTU (“X_003”) in the freshwater microbiome experiment, the histograms of read counts across replicate communities are overlaid with predictions from the mixture model (panel B). The data of Day 2 at each inoculum dilution rate are shown (see Figure S15 for results on other OTUs). (D) Relationship between initial stochasticity and multimodality. By targeting the OTUs commonly observed across experimental replicates (the section “Multimodality and within‐mode variation in OTU abundance” in Supporting Information), the relationship between the estimated CV of cell numbers upon inoculation (panel A) and multimodality was examined for each source microbiome type (soil or freshwater) at each time point. The multimodality estimates are scaled from 0 to 1 across the panels. Lines represent significant linear regressions (FDR < 0.05; Table S7). (E) Relationship between initial stochasticity and within‐mode variation. The within‐mode variation scores are scaled from 0 to 1 across the panels.

We assumed that initial stochasticity, defined as stochastic among‐replicate variation in each species' abundance (the number of individuals of each species) arising from demographic sampling, could be decomposed into population‐level (OTU‐level) statistical distributions. Accordingly, the cell‐count data for each OTU in each inoculum were used to quantify the magnitude of stochasticity at the foundation of experimental microbiomes. Specifically, we modelled the OTU cell counts in each sample assuming a multinomial distribution. This distribution was parameterised by the mean total number of cells in the inoculum suspension and the relative abundance of each OTU in the suspension (Figures S2 and S3). This approach allowed us to simulate cell numbers as multinomial random samples across the 96 replicates (covering 2 sources × 4 dilution rates) and subsequently calculate their expected coefficient of variation (CV). The resulting CV values were used as quantitative measures of initial stochasticity upon inoculation at the OTU level (Figures 2A and S4). We confirmed that the CV values significantly increased with increasing inoculum dilution rates (Table S2).

### 2.5 Multimodality and Within‐Mode Variation in OTU Abundance

For each inoculum setting, we examined the degree to which the abundance of each prokaryotic OTU varied among replicate community samples at each time point after the inoculation event. Based on the stability landscape concept of community assembly, among‐sample variation in OTU abundance was quantified sequentially with two types of indices (Figure 1A, right).

For the first step, the presence of multiple peaks in the histogram of each OTU's abundance across replicate samples was examined with a “multimodality” index, which gives insights into the presence of multiple basins of attraction in the microbiome assembly (Figure 2B). A scaled excess mass statistic calculated in a multimodality test (Ameijeiras‐Alonso et al. 2019) was used as a multimodality measure (Supporting Information and Figures S5, S6).

Next, for each peak identified in the histogram, “within‐mode” variation among replicate samples was calculated by assuming fluctuations within each basin of a stability landscape. Here, within‐mode variation was expected to capture the variability among replicate communities that fall within the same basin of attraction, primarily arising from initial stochasticity. To quantify this within‐mode variation, the frequency distribution of OTU abundance within each peak (mode) was assumed to follow a mixture model of a binomial distribution and a standard normal distribution (Figure 2B).

We then performed a linear regression analysis to examine whether each type of among‐replicate variation could increase with increasing initial stochasticity, which was evaluated by the CV of expected cell number for each OTU. When multiple peaks were present in a histogram, within‐mode variation was calculated for each peak, and the average of these within‐mode variation scores was used as the metric (Supporting Information and Tables S3, S4). Prior to the linear regression analysis of multimodality, raw relative abundance data were subjected to a centered log‐ratio (CLR) transformation to avoid potential artefacts in the calculation of the multimodality index.

### 2.6 Community‐Scale Differentiation Among Replicates

**FIGURE 3:**
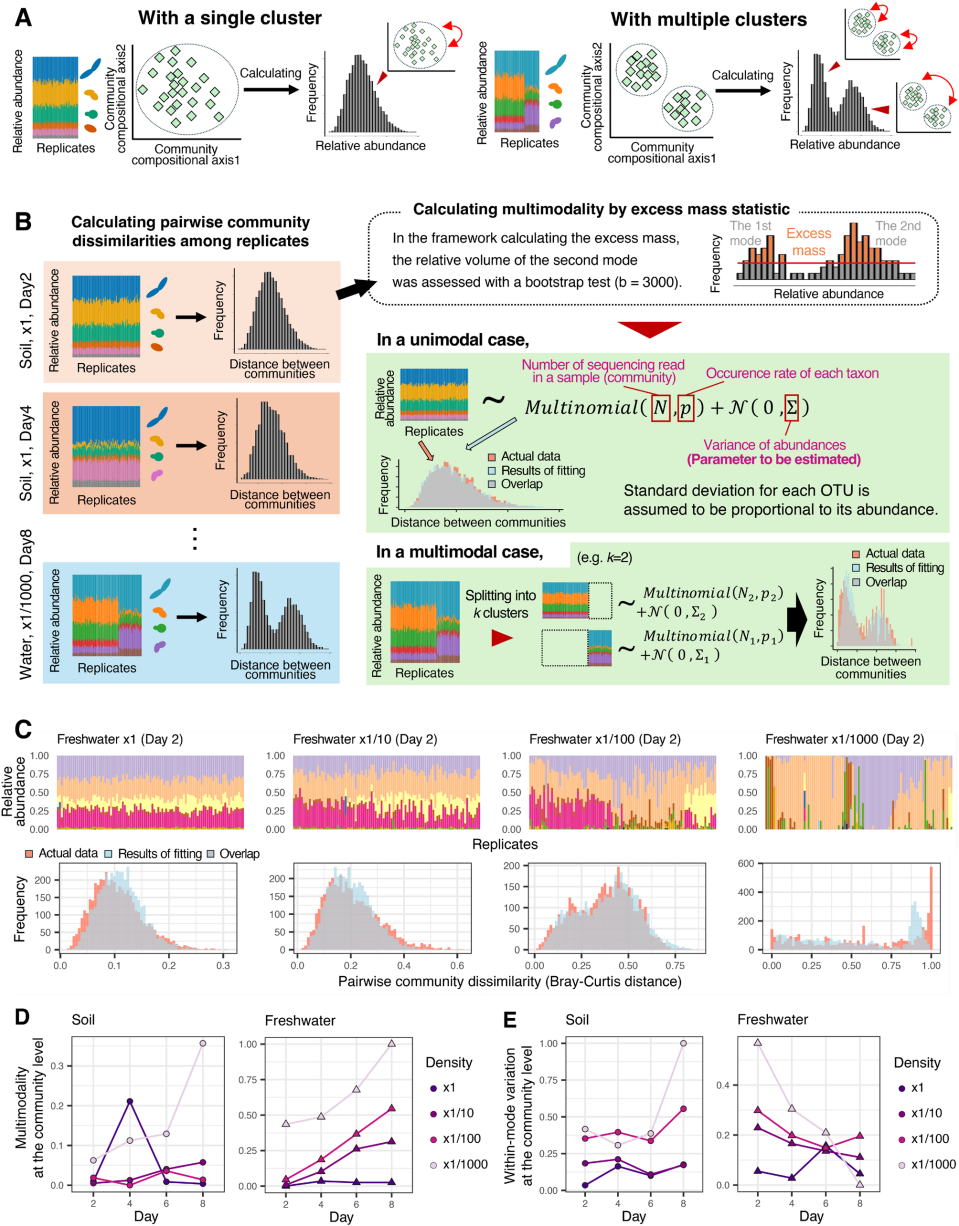
Among‐replicate divergence in community compositions. (A) Patterns in pairwise community dissimilarity and underlying community structure. If multiple peaks are present within a histogram of among‐replicate community dissimilarity, replicate communities would be divided into clusters according to their compositions. (B) Multimodality and within‐mode variation in community dissimilarity histograms. The degree to which a histogram of pairwise community dissimilarity exhibited multiple peaks was assessed as a community‐scale measure of multimodality. For each peak identified in the multimodality analysis, among‐replicate variation was assumed to follow multinomial and standard normal distributions. Within‐mode variation is then evaluated as the standard deviation of the standard normal distribution. (C) Examples of community dissimilarity histograms. For freshwater‐derived experimental microbiomes, the histograms of community dissimilarity among replicates are overlaid with predictions from the mixture model (panel B). The data of Day 2 at each inoculum dilution rate are shown (see Figure S17 for full results). (D) Temporal changes in multimodality. For each combination of source microbiome type (soil or freshwater) and inoculum dilution rates, the temporal trends of community‐scale multimodality are shown. Multimodality estimates are scaled from 0 to 1 across the panels. (E) Temporal changes in within‐mode variation. The community‐scale estimates of within‐mode variation are scaled from 0 to 1 across the panels.

By extending the statistical approach applied at the OTU level analysis, we next developed a method for quantifying community‐level differentiation. Instead of the distribution of each OTU's abundance across samples, we focused on the distribution of pairwise dissimilarities (Bray–Curtis *β*‐diversity) between replicate samples (Figures 3A,B and S7). The multimodality of pairwise community dissimilarity distributions was calculated for each inoculum setting at each time point to evaluate the extent to which replicate communities diverged into multiple basins of community structure. Likewise, variation in community structure within each basin of the stability landscape was inferred by quantifying within‐mode variation in these distributions. For each inoculum setting at each time point, we checked whether multiple peaks existed within the histogram of pairwise community dissimilarity. In general, the presence of multiple peaks within the distribution of pairwise dissimilarities indicates that the focal community dataset includes multiple clusters of data points (Figure 3A; Supporting Information) (Hayashi et al. 2024).

### 2.7 Stability Landscapes and Dynamic Regimes

In the analysis of multimodality and within‐mode variation, we assumed the presence of a fixed stability landscape for each of the two experimental communities (Framework 1). An alternative approach to interpret the community data is to assume that initial stochasticity determines the topography of stability landscapes itself (Framework 2). To gain complementary insights into community assembly, we statistically inferred stability landscape architecture and the trajectories of ecological dynamics based respectively on the two conceptual frameworks.

To infer stability landscape architecture, we performed a statistical‐physics‐based approach for statistically modelling the relationship between systems' states and their probabilities (Suzuki et al. 2021; Masuda et al. 2025). In the platform of “energy landscape analysis” (Suzuki et al. 2021; Fujita et al. 2023a, 2025), the probability of observing a given community state (e.g., OTU membership) is inferred using an Ising model under a maximum‐entropy criterion. The “energy” of a community state is then defined as the negative logarithm of its modelled probability of observation (Supporting Information). For Framework 1, we inferred the “energy landscape” architecture for each source community (soil or freshwater) using the combined dataset including all the four dilution settings. For Framework 2, which assumes the dependence of stability landscape topography on initial stochasticity level (Figure 1B), the energy landscape architecture was inferred for each of the eight inoculum settings. The energy landscapes were visualised on a two‐dimensional community state space defined by metric multidimensional scaling (mMDS) based on Bray–Curtis dissimilarity.

We next examined how the trajectories of ecological dynamics diverged among the experimental replicates by applying “ecological dynamic regime” analysis (Sánchez‐Pinillos et al. 2023). In the statistical platform, a “dynamic regime” was defined as a group of ecological trajectories representing temporal community shifts within a multidimensional space of community states. Within each regime, a “representative trajectory” passing through the densest regions within the state space was used to summarise the dominant assembly pattern. The ecological dynamic regime analysis was applied, in parallel, to the Framework 1 (all the four dilution rates) and Framework 2 (each dilution rate) data structures. To test whether the ecological trajectories, which reflected the underlying stability landscape topography, diverged more greatly at higher initial stochasticity (i.e., hypothesis addressed in Framework 2), we quantified the dispersion of ecological trajectories in state space for each inoculum dilution rate using the following three complementary metrics as implemented with the R ecoregime 0.2.0 package (Sánchez‐Pinillos et al. 2023).

The dynamic dispersion (dDis) quantifies the average dissimilarity of each trajectory from the representative trajectory. The dynamic dispersion index is calculated as follows:

$$\text{dDis}=\frac{\sum_{i=1}^{m}d_{\mathrm{i\alpha}}}{m},$$

where $d_{\mathrm{i\alpha}}$ represents the directed segment path dissimilarity (De Cáceres et al. 2019) between a trajectory i and a reference trajectory α, and m is the number of observed trajectories (i.e., the number of replicate time series). The criteria for selecting the reference trajectories are detailed in Supporting Information.

The dynamic beta‐diversity (dBD) quantifies the average dissimilarity between community trajectories as follows:

$$\mathrm{dBD}=\frac{\sum_{i=1}^{m-1}\sum_{j=i+1}^{m}{d_{\mathrm{ij}}}^{2}}{m\left(m-1\right)},$$

where $d_{\mathrm{ij}}$ denotes the directed segment path dissimilarity between trajectories i and j. These two metrices vary from 0 (completely identical dynamics among replicates) and 1 (complete differentiation of temporal community dynamics).

The dynamic evenness (dEve) measures the continuity of trajectory variation within the dynamic regime as follows:

$$\text{dEve}=\frac{\sum_{c=1}^{m-1}\min\left(\frac{d_{\mathrm{ij}}}{\sum_{c=1}^{m-1}d_{\mathrm{ij}}},\frac{1}{m-1}\right)-\frac{1}{m-1}}{1-\frac{1}{m-1}},$$

where c is the edges of a minimum spanning tree constructed from the set of trajectories forming a dynamic regime. In contrast to the dynamic dispersion and dynamic beta‐diversity, a higher value of dynamic evenness indicates a lower level of divergence among the time series of replicate communities. It ranges from 0 (when many subclusters of trajectories exist) and 1 (when all trajectories are evenly distributed).

## 3 Results

### 3.1 Inoculum Communities

The estimated number of prokaryotic cells contained in ×1 inocula were 2.59 × 10^6^ for the soil source community and 2.30 × 10^5^ for the freshwater source community (Table S5). The soil source community consisted of diverse taxa of bacteria, such as *Pseudomonas*, *Stenotrophomonas*, *Burkholderia*, *Flavobacterium*, and *Klebsiella*, whereas the freshwater source community was dominated by *Pseudomonas* (Figure S8). In total, 102 OTUs (50 genera) and 62 OTUs (41 genera) were observed in the soil and freshwater source communities, respectively. The Shannon's diversity of the OTUs were 3.53 and 2.03 (Shannon effective number: 34.1 and 7.61) for the soil and freshwater source communities, respectively.

### 3.2 OTU‐Level Differentiation Among Replicates

For both soil and freshwater source communities (Figures S9–S13), OTU‐level multimodality significantly increased with increasing estimated CV of cell numbers upon inoculation, except for Day 8 of the soil‐inoculum experiment (Figures 2C,D, S14–S16). A supplementary analysis suggested that multimodality increased after the establishment of the experimental communities (Figure S14).

Within‐mode variation in the OTU abundance distributions significantly increased with increasing estimated CV of cell numbers upon inoculation at Days 2 and 4 for both inoculum source communities (Figure 2E; Table S7). This relationship between within‐mode variation and initial stochasticity decayed through time in the experiment.

### 3.3 Community‐Scale Differentiation Among Replicates

Multimodality in the distribution of dissimilarity among replicate communities (Figure S17; Table S8) was basically greater at higher dilution rates upon inoculation (Figure 3C,D). Likewise, within‐mode variation in the distribution of community dissimilarity was higher at higher dilution rates on both the soil and freshwater inoculum experiments (Figure 3E).

In terms of temporal dynamics, the community‐level multimodality values increased over time, although an abrupt increase in multimodality was observed in the ×1 soil‐inoculum setting at Day 4 (Figure 3D). This exceptional pattern at Day 4 was attributed to transient occurrences of replicate communities with abrupt structural changes: such peculiar community compositions disappeared at Day 6 (Figure S9A). Notably, the multimodality values calculated from simulated inoculum communities were lower than that observed at Day 2 and at subsequent time points (Figure S18).

In contrast to the overall pattern observed in the multimodality analysis, within‐mode variation in community dissimilarity decreased over time in the freshwater‐inoculum experiment (Figure 3E). Within‐mode variation in the soil‐inoculum experiment exhibited no clear temporal trends.

### 3.4 Stability Landscapes and Dynamic Regimes

**FIGURE 4:**
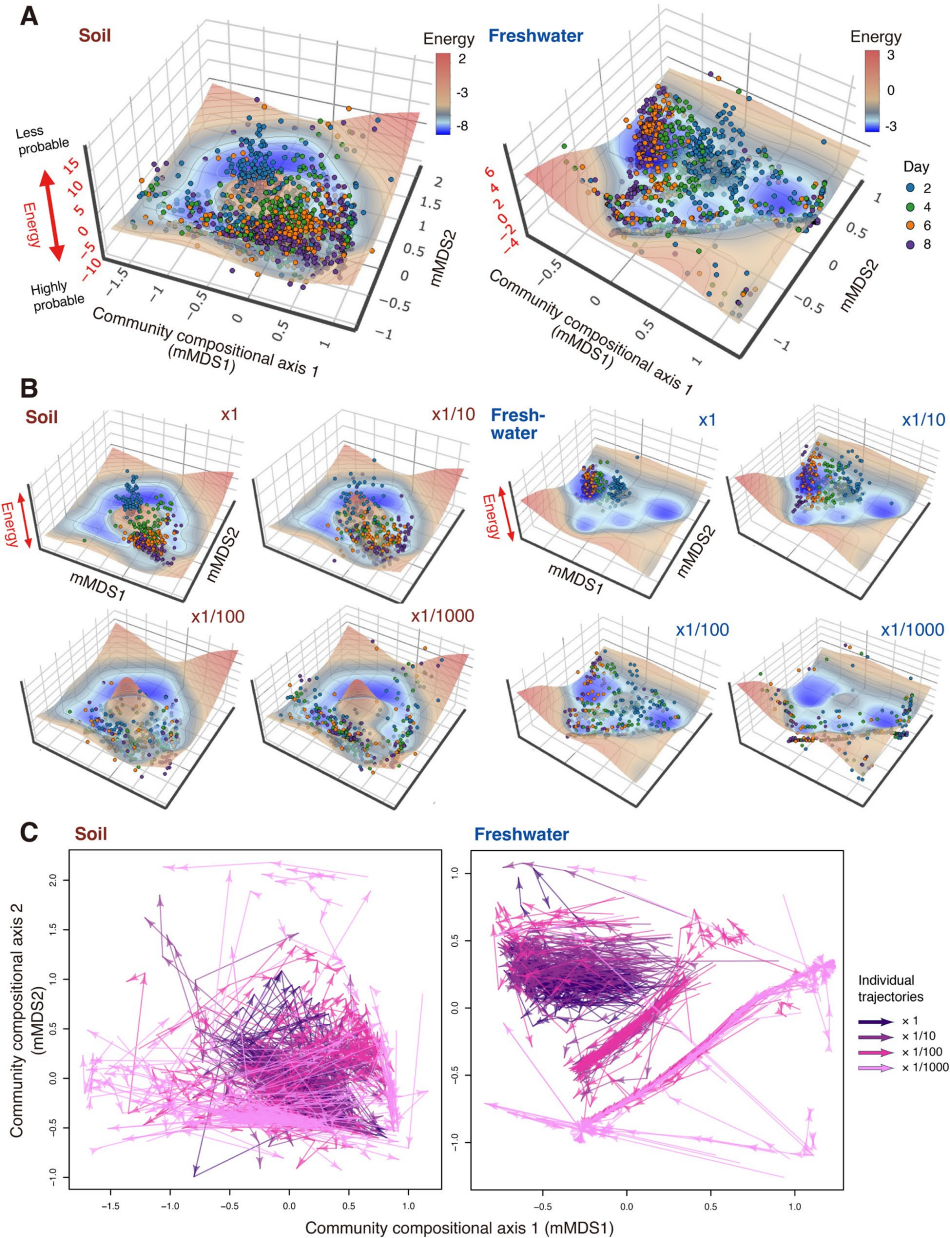
Distributions of community states on the inferred stability landscape. (A) Energy landscape analysis. For each source microbiome type (left, soil; right, freshwater), energy landscape analysis was performed to infer the topography of a stability landscape. In each graph, community states are shown on the two‐dimensional space defined by a metric multidimensional scaling (mMDS), while the vertical axis shows the “energy” metric, which represents the “improbability” of observing a specific community state. On the “energy landscape”, a lower value indicates a more probable community composition. The analysis was conducted by assuming that stability landscape architecture was unchanged across inoculum dilution rates (Framework 1). (B) Dependence of community‐state distributions on initial stochasticity. The data points corresponding to different inoculum dilution rates are shown separately on the inferred stability (energy) landscapes (panel A). See Figure S19 for views of the landscapes from another angle. (C) Ecological dynamic regime analysis. On each two‐dimensional surface of mMDS, the time series of community dynamics are shown respectively for replicate communities (arrows). Arrow colours correspond to inoculum dilution rates.

When the stability (energy) landscape topography was inferred using Framework 1, replicate communities were distributed differently across dilution‐rate settings (Figures 4A,B and S19). In both the soil‐ and freshwater‐inoculum experiments, community states were confined to a narrow region on the energy landscape at the ×1 dilution rate. In contrast, at higher dilution rates, community states became increasingly dispersed toward the margins of the basins, with the strongest scattering observed at the ×1/1000 dilution rate (Figures 4A,B and S19). Alongside the energy landscape patterns, the ecological dynamic regime analysis revealed diverse ecological trajectories, characterised by convergence toward basin centers as well as transitions between basins (Figure 4C).

**FIGURE 5:**
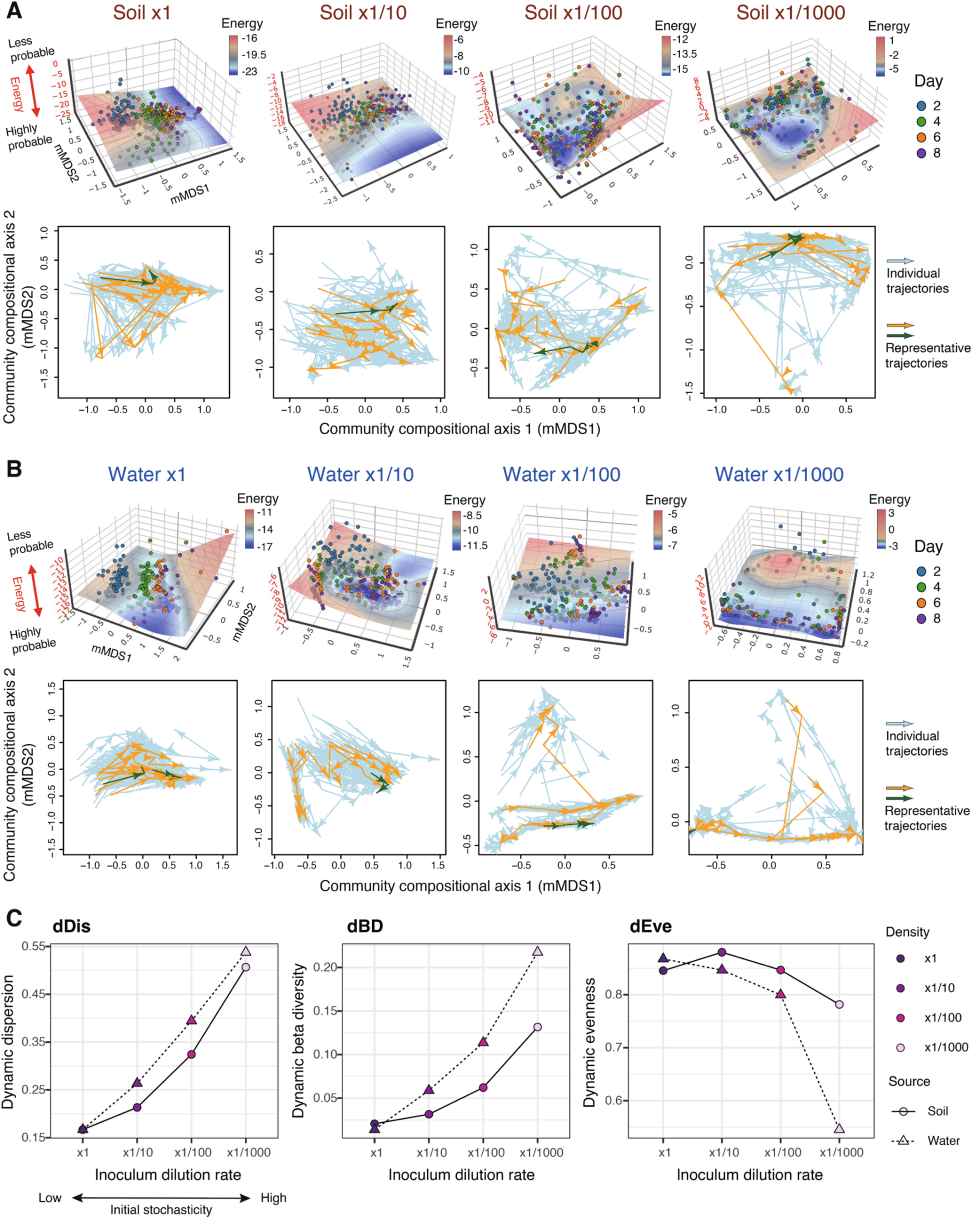
Initial stochasticity and the divergence of ecological trajectories. (A, B) Changes in stability landscape topography and ecological trajectories across initial stochasticity levels. By assuming that stability landscape topography could change depending on initial stochasticity level (Framework 2), energy landscape analysis (upper panels) and ecological dynamic regime analysis (lower panels) were applied to each combination of source microbiome type (soil or freshwater) and inoculum dilution rates. (C) Quantifying the divergence of ecological trajectories. Based on ecological dynamic regime analysis, we quantitatively evaluated the dispersion of ecological trajectories for each inoculum setting using the following three indices. A larger value of the dynamic dispersion (dDis) or dynamic beta‐diversity (dBD) metrics indicated greater differentiation in community‐dynamics trajectories among experimental replicates. In contrast, a smaller dynamic evenness (dEve) value indicates greater divergence of ecological trajectories. For each of the eight inoculum settings, the representative trajectory shown with green arrows was used as a reference to calculate the dynamic dispersion index.

Energy landscape analysis based on Framework 2 illustrated how energy landscape architecture could change depending on inoculum dilution rates (Figures 5A,B, S20–S22). The community compositions of the same time points clustered on the inferred energy landscape under the ×1 inoculum condition (Figure 5A,B). In contrast, such time‐point‐specific clusters decayed with increasing inoculum dilution rates.

Ecological dynamic regime analysis further indicated that the directions of temporal community dynamics varied among replicate samples more conspicuously at higher inoculum dilution rates (Figure 5C). In fact, dynamic dispersion and dynamic beta‐diversity, which represented the extent to which the trajectories of community assembly varied among replicates, increased with increasing inoculum dilution rates (Figures 5C and S23). As expected by the trends, dynamic evenness, which represented the continuity of variation within the trajectory space of community assembly, decreased with increasing inoculum dilution rates.

## 4 Discussion

By developing a highly parallelised system for tracking temporal community dynamics, we experimentally evaluated the roles of stochasticity at the foundation of communities. In the context of stochastic ecological processes, a number of studies have been designed to demonstrate how the order of species' arrival may influence community assembly [i.e., priority effects (Fukami 2015; Debray et al. 2022; Szabo et al. 2022)]. Alongside arrival order, the size of arriving communities critically impacts early assembly, eventually determining the level of community structural differentiation into alternative transient/stable states. With the multi‐replicated experimental design, we directly examined the extent to which slight stochastic differences could generate diverged community transitions. Such quantitative evaluation of stochasticity provides a platform for understanding community assembly through the lens of statistical and physical approaches to complex systems (Azaele et al. 2016).

In our experimental setting, processes generating compositional differences among replicate communities can be conceptually divided into stochasticity at community foundation (initial stochasticity) and fluctuations throughout post‐inoculation periods (perpetual ecological drift). Initial stochasticity is defined as an instantaneous property, and it can be quantified using simulations based on multinomial distributions (Figure 2D,E). In contrast, ecological drift operates continuously during community assembly, with its relative strength changing over time (Beisner et al. 2003; Chase and Myers 2011; Vellend et al. 2014; Shoemaker et al. 2020), making it challenging to quantify. Rigorous disentanglement of the effects of initial stochasticity and perpetual ecological drift requires comparative analyses using experimental systems in which founding community compositions are fully equalised across replicate communities (Gilbert and Levine 2017). In the following discussion, we assume the combined influence of initial stochasticity and subsequent ecological drift.

The multimodality analysis showed that the higher dilution rates (higher initial stochasticity) of founding communities resulted in greater levels of divergence into discrete OTU‐ and community‐level patterns among replicate microbiomes (Figures 2D, 3D, S9). This result suggests that increased stochastic variation in community states at early community assembly facilitated splitting into alternative basins of attraction on a stability landscape. In other words, the reproducibility of community dynamics per se is controllable if we can limit or augment stochasticity at early assembly by adjusting the size of arriving communities (Scheffer and Carpenter 2003; Siqueira et al. 2020; Lopes et al. 2024).

The quantitative approach of this study provided an opportunity for exploring the critical community size beyond which the reproducibility of community dynamics could drastically change. Basically, differentiation into discrete community compositions was observable even at higher initial densities (Figure S9). However, the divergence of community compositions became striking when the total cell number of founding prokaryotic communities was less than the order of 10^4^ (Figures 2D, 3D, S9). Such splitting phenomena may stem from slight differences among replicates in the balance between dominant species (Goldford et al. 2018). Alternatively, among‐replicate variation in the presence/absence of rare species might have disproportionate influence on the divergence of ecological dynamics (Leitão et al. 2016; Jousset et al. 2017). Indeed, “extinctions” of OTUs in founding communities may have occurred more frequently at higher dilution rates (Figure S18A,B), yet such variation did not translate into distinct alternative states, indicating that extinction alone is insufficient to generate multimodality (Figure S18C,D). In both scenarios, the strength of combined stochastic effects would depend not only on the ratio of founding communities to their source communities (i.e., inoculum dilution rates in this study), but also on the diversity and heterogeneity of the species pools (Santillan and Wuertz 2022; Le Moigne et al. 2023). Specifically, when a species pool includes only a few species with identical abundance, highly consistent assembly patterns will be observed across replicate communities even with small founding community size (e.g., 10^3^). As the level of initial stochasticity can be simulated based on species pool data (Figure 2A), comparative analyses of source communities with varying alpha‐diversity will yield further quantitative insights into how founding community size relates to the reproducibility of community dynamics.

Along with the multimodality index for inferring divergent ecological consequences, we applied a statistical platform for quantifying the level of fluctuations within each basin of attraction. The approach integrating binomial distributions with normal distributions is broadly applicable when evaluating the extent to which combined stochastic effects cause fluctuations in each species' population dynamics (Figure 2B), and the analysis extends to the community level by combining multinomial and normal distributions to estimate fluctuations around community state attractors (Figure 3A,B). By calculating the indices, we confirmed that greater initial stochasticity could lead to larger within‐basin fluctuations, consistent with stronger combined stochastic effects (Figures 2E and 3E). However, a relatively low level of “within‐basin” variation was observed for the freshwater‐derived microbiomes with the highest dilution rate at Day 8. Considering that the freshwater source community exhibited lower alpha‐diversity (lower OTU richness) than the soil source community (Figures S3 and S4), the high initial stochasticity setting would have caused rapid divergence into alternative basins (Figures 2 and 3). Within each basin, ecological drift and deterministic processes, such as competition among species with similar niches, may have caused extinctions of constituent species, thereby limiting the range of variation across replicate communities representing the same basin (Chase and Myers 2011). The community structure of the ×1/1000 freshwater inoculum communities at Day 8 was characterised by the monodominance of alternative *Pseudomonas* OTUs (Figure S9B), suggesting that competitive exclusion had occurred between *Pseudomonas* species. It remains an important challenge to explore the presence of any positive feedback mechanisms that could reinforce such monodominant states (e.g., antibiotics or pollutants inhibiting the growth of competitors) (Khare and Tavazoie 2015; Stubbendieck and Straight 2016).

Beyond compositional divergence among replicate communities, ecosystem functions may also systematically vary with initial stochasticity. In the freshwater‐inoculum experiment, the dominance of a single or a few *Pseudomonas* OTUs was observed at the highest dilution rate (×1/1000) as discussed above. In contrast, at the lowest dilution rate (×1), consistent patterns characterised by the coexistence of *Pseudomonas*, *Yersinia*, and *Raoultella* were observed across replicate communities (Figure S9B). Since these genera differ in their functional profiles (Peng et al. 2024), the community‐scale functioning of the three‐genus coexistence state is expected to differ from the monodominance state of *Pseudomonas*. Thus, qualitative changes in ecosystem functions could result from quantitative changes in initial stochasticity. In other words, the repertoires of alternative transient/stable states, which may differ in functional profiles, could drastically change depending on stochasticity at community foundation (Abbott and Nolting 2017).

The possibility that the repertoire of basins or dynamic regimes depends on initial stochasticity level was further examined with emerging statistical platforms. Under the assumption of a fixed stability‐landscape topography (Chase 2003; Vellend et al. 2014) (Framework 1; Figure 1A), the distribution of community states within the inferred energy landscapes varied markedly across dilution rates of the founding communities (Figure 4A,B). This result suggests that initial stochasticity level could determine the sets of “available” basins and dynamic regimes (Figure 4C).

An alternative interpretation arises when stability landscape topology itself is allowed to vary along an initial stochasticity gradient (Framework 2; Figure 1B). In this view, initial stochasticity level can be treated as a hyperparameter that determines community assembly rules (Chase 2003; Vellend et al. 2014). In our analysis, at the lowest level of initial stochasticity, community compositions at the same time points clustered tightly and moved from hilltops toward valleys on the inferred landscapes, indicating that strong deterministic processes governed community assembly (Figure 5A,B). In contrast, at the highest initial stochasticity level, community compositions did not form clear time‐point‐specific clusters, suggesting that elevated stochasticity drove divergent community dynamics among replicate samples. Consistently, we found that ecological trajectories diverged more strongly with increasing initial stochasticity (Figure 5C). Together, these results highlight the need to extend the concept of dynamically changing stability landscapes (Scheffer and Carpenter 2003; Suzuki et al. 2021) and ecological dynamic regimes (Sánchez‐Pinillos et al. 2023, 2024) in addition to classic conceptual frameworks that assume fixed landscape architecture.

Although we developed a high‐throughput way for systematically evaluating the ecological outcomes of stochasticity, the limitations of the present experimental design need to be acknowledged. First, our experiments with deep‐well plates would have restricted the number of coexisting species in each replicate community. In small habitats, species extinctions would often occur via ecological drift (Hubbell 2005), and limited refuges for inferior competitors would promote the dominance of superior species (Tilman 1994). Therefore, the experimental design should be extended to reveal how the relationship between initial stochasticity and the diversity of ecological consequences can vary along the axis of culture volumes (Kram and Finkel 2014). Second, despite the use of a mechanised experimental system, uncontrolled environmental heterogeneity (e.g., temperature) may have introduced unintended deterministic effects. However, we detected no systematic spatial patterns in community outcomes within culture plates (Figures S24 and S25). Third, the observation period may have been insufficient for observing the alternative stable states of community compositions (Figure S26). Even if the monodominant states observed at Day 8 in some inoculum settings (e.g., ×1/1000 freshwater) seemingly represent the destinations of community states, the resurgence of other bacteria enduring at very low densities may occur if the sampling period is extended. It is generally difficult to distinguish alternative transient states from alternative stable states (Fukami and Nakajima 2011; Hayashi et al. 2024). Therefore, we did not use the term “alternative stable states” when interpreting the experimental results. In this sense, the terms “alternative trajectories” or “alternative dynamic regimes” can be broadly used without making controversial definitions of transience and steadiness in community dynamics (Scheffer and Carpenter 2003; Mayer and Rietkerk 2004; Angeler and Allen 2016). Ecological dynamic regime analysis provides a practical approach for quantitatively evaluating how community dynamics are differentiated based on combined stochastic effects. Fourth, although we used fresh media in the experiment to examine the influence of initial stochasticity, such assembly in vacant environments rarely occurs in the wild. Consequently, it would be intriguing to redesign the experimental approach to evaluate how the size and arrival timing of immigrant communities can influence the divergence of resident communities' dynamics.

Further insights into the interplay of deterministic and stochastic processes will not only enhance our basic understanding of community assembly but also advance applied sciences for controlling community‐scale structure and functions. For example, in the use of plant‐associated microbes in agriculture (Carlström et al. 2019), determining the founding community size of biostimulant microbiomes is essential for yielding stable functional benefits. Likewise, in the management of human gut microbiomes, reproducibility in the outcomes of microbiome transplantation (Green et al. 2020; Mingaila et al. 2023) would be quantitatively evaluated along the axis of introduced bacterial community size. Alongside deepening the knowledge of stochastic processes, it is crucial to build a systematic understanding of deterministic processes when we develop frameworks for predicting and controlling community dynamics. If genomic information is available for the species constituting a community, the level of resource competition or metabolic exchange could be inferred at the community level (Zelezniak et al. 2015; Dukovski et al. 2021). Thus, synthetic community experiments using genome‐sequenced bacterial species or strains (Carlström et al. 2019; Vrancken et al. 2019), as well as shotgun metagenomic analyses of field‐collected source microbiomes (Frioux et al. 2020; Fujita et al. 2023b), will help us evaluate the extent to which community‐scale consequences can be predicted based on our understanding of deterministic processes. Systematic tests in microbial model systems will accelerate feedback between theoretical and empirical studies on temporal community dynamics, shedding new light on the assembly rules of macro‐organisms.

## Author Contributions

I.H. and H.T. designed the work. I.H. performed the experiments. I.H. analysed the data with H.T. and M.S.‐P., I.H. and H.T. wrote the paper with M.S.‐P.

## Funding

This work was supported by Kyoto University CeLiSIS Program (23CeLiSIS‐02), Core Research for Evolutional Science and Technology (JPMJCR23N5), Japan Society for the Promotion of Science (24KJ1454), New Energy and Industrial Technology Development Organization (202302), Fusion Oriented REsearch for disruptive Science and Technology (JPMJFR2048).

## Conflicts of Interest

H.T. is the founder and a director of Sunlit Seedlings Ltd. The other authors declare that the research was conducted in the absence of any commercial or financial relationships that could be construed as a potential conflicts of interest.

## Supporting information


**Figure S1:** Rarefaction curve of culture samples and inoculum samples. Relationships between the number of sequencing reads and the number of detected prokaryote ASVs are shown. (A) Rarefaction curves of 50 samples randomly selected from the pool of the samples with 3000 or more sequencing reads are shown in each panel. The data of Day 2 and Day 8 are shown. (B) Rarefaction curves of the soil and freshwater source (inoculum) microbiomes.
**Figure S2:** Estimated number of cells for each OTU in the founding communities (soil inoculum). Based on simulations with multinomial distribution model, the number of cells upon inoculation was estimated for each OTU, and the variability was derived from 96 independent simulation replicates (see Materials and Methods for details). Results for the four inoculum dilution rates are separately shown.
**Figure S3:** Estimated number of cells for each OTU in the founding communities (freshwater inoculum). Based on simulations with multinomial distribution model, the number of cells upon inoculation was estimated for each OTU (see Materials and Methods for details). Results for the four inoculum dilution rates are separately shown.
**Figure S4:** Estimated CV of cell numbers upon inoculation. (A) Coefficients of variation (CVs) in the estimated number of cells for each OTU in the founding communities among replicate communities. For each panel representing a inoculum setting (4 source community type × 4 dilution rates), data points represent individual OTUs. OTUs with higher relative abundance in the inoculum exhibited lower CVs across replicates. (B) Log10‐transformed CVs.
**Figure S5:** OTU‐level multimodality calculated using an alternative metric. To the validate multimodality values calculated with the ACR method (Ameijeiras‐Alonso et al. 2019) (Figure 2D), we performed a supplementary analysis using the HH method (Hartigan and Hartigan 1985). (A) Relationship between multimodality estimates using the two alternative methods. (B) Relationship between the *p*‐values of multimodality tests obtained from the two alternative methods. (C) Relationship between initial stochasticity and multimodality (results based on HH method). By targeting the OTUs commonly observed across experimental replicates (the section “Multimodality and within‐mode variation in OTU abundance” in Supporting Information), relationship between the estimated CV of cell numbers upon inoculation and multimodality was examined for each source microbiome type (soil or freshwater) at each time point. The multimodality estimates are scaled from 0 to 1 across the panels. Lines represent significant linear regressions (FDR < 0.05). (D) Relationship between estimated CV of cell numbers upon inoculation and within‐mode variation. Note that multimodality test performed prior to the calculation of within‐mode variation was more stringent in the HH method than the ACR method (panel B).
**Figure S6:** Multimodality at the community level calculated using an alternative metric. To the validate multimodality values calculated with the ACR method (Ameijeiras‐Alonso et al. 2019) (Figure 3D), we performed a supplementary analysis using the HH method (Hartigan and Hartigan 1985). (A) Relationship between the *p*‐values of multimodality tests obtained from the two alternative methods. (B) Relationship between the *p*‐values of multimodality tests obtained from the two alternative methods. (C) Temporal changes in multimodality (results based on HH method). For each combination of source microbiome type (soil or freshwater) and inoculum dilution rates, the temporal trends of community‐scale multimodality are shown. Multimodality estimates are scaled from 0 to 1 across the panels. (D) Temporal changes in within‐mode variation. The community‐scale estimates of within‐mode variation are scaled from 0 to 1 across the panels. Note that multimodality test performed prior to the calculation of within‐mode variation was more stringent in the HH method than the ACR method (panel B).
**Figure S7:** Community‐scale multimodality and within‐mode variation calculated with alternative community dissimilarity metrices. To confirm the results based on Bray–Curtis dissimilarity (Figure 3), we calculated multimodality and within‐mode variation based, respectively, on Jensen–Shannon distance and Hellinger distance. (A) Multimodality based on Jensen‐Shannon distance. (B) Within‐mode variation based on Jensen‐Shannon distance. (C) Multimodality based on Hellinger distance. (D) Within‐mode variation based on Hellinger distance.
**Figure S8:** Taxonomic compositions of soil and freshwater source (inoculum) microbiomes. For each of the soil (A) and freshwater (B) source microbiomes, community compositions are respectively shown at the family, genus, and ASV levels.
**Figure S9:** Overview of among‐replicate variation in community structure. (A) Experiment with the soil inoculum microbiome. Temporal changes in the 99% OTU‐level community compositions (relative abundance) are shown. For each inoculum dilution rate, replicate communities were ordered based on the results of unweighted pair group method with arithmetic mean (UPGMA) analyses performed at Day 8. The founding community size estimated based on quantitative amplicon sequencing is shown for each inoculum setting. (B) Experiment with the freshwater inoculum microbiome. The experimental microbiomes deriving from the soil source community were constituted mainly by the five genera, *Klebsiella*, Pseudomonas, Raoultella, Stenotrophomonas, and Serratia, while those deriving from the freshwater source community were dominated by the three genera, *Pseudomonas*, Yersinia, and Aeromonas. For both types of inoculum communities, the OTU‐level compositions of experimental communities varied more conspicuously among replicate communities at higher dilution rates. Such elevation of among‐sample variation in community compositions with increasing dilution rate was observed as well at the ASV‐ and genus‐level analyses (Figures S10 and S11).
**Figure S10:** Overview of among‐replicate variation in community structure (ASV‐level results). (A) Experiment with soil inoculum microbiome. Temporal changes in the ASV‐level community compositions (relative abundance) are shown. For each inoculum dilution rate, replicate communities were ordered based on the results of unweighted pair group method with arithmetic mean (UPGMA) analyses performed on Day 8. (B) Experiment with freshwater inoculum microbiome.
**Figure S11:** Overview of among‐replicate variation in community structure (genus‐level results). (A) Experiment with soil inoculum microbiome. Temporal changes in the genus‐level community compositions (relative abundance) are shown. For each inoculum dilution rate, replicate communities were ordered based on the results of unweighted pair group method with arithmetic mean (UPGMA) analyses performed on Day 8. (B) Experiment with freshwater inoculum microbiome.
**Figure S12:** Alpha diversity of the experimental microbiomes. Temporal changes in the Shannon's diversity index (left) and richness (right) of 99% OTUs are shown for the soil (top) and freshwater (bottom) experimental microbiomes. The results of Student's t‐tests comparing each pair of time points are presented in Table S6. In each combination of source microbiomes and inoculum dilution rates, alpha‐diversity decreased through time in the microbiome experiment, and the average of Shannon's diversity of the OTUs was 0.902 (Shannon effective number = 2.54) for the soil‐ and 0.743 (Shannon effective number = 2.25) for the freshwater‐derived communities at Day 8.
**Figure S13:** Alpha diversity of the experimental microbiomes. Temporal changes in the Shannon evenness (left) and Shannon effective number (right) of 99% OTUs are shown for the soil (top) and freshwater (bottom) experimental microbiome.
**Figure S14:** Temporal changes in OTU‐level multimodality. Temporal changes in multimodality for each OTU from Day 0 to Day 8 are shown. Multimodality values at Day 0 were obtained based on the multinomial‐distribution simulation of founding communities (Figures S2 and S3), whereas those from Day 2 to Day 8 were derived from experimental observations (Figure 2D,E). Each panel shows results for a single OTU at a given inoculum dilution rate. The red dashed line indicates the multimodality value at Day 0. (A) Multimodality in the soil‐derived inoculum experiment. (B) Multimodality in the freshwater‐derived inoculum experiment.
**Figure S15:** Histograms of OTU abundance across replicate communities. Each histogram is overlaid with predictions from the mixture model (see Materials & Methods). The red curve in each panel represents the model's prediction. Each panel shows the abundance distribution for a specific combination of inoculum source, sampling date, dilution rate, and OTU. The combination is indicated at the top of each panel (e.g., in “Soil_2_1_X_001”, the data represent a non‐diluted soil‐derived inoculum sampled on Day 2, with a focus on OTU X_001).
**Figure S16:** Supplementary results on OTU‐level multimodality and within‐mode variation. (A) Temporal changes in multimodality and within‐mode variation for each OTU. Only OTUs observed under at least three different initial density conditions on at least one sampling day are shown. The inoculum source and the focal OTU are indicated at the top of each panel (e.g., “Soil_X_001” denotes a soil‐derived inoculum with a focus on OTU X_001). (B) Relationship between multimodality and within‐mode variation. No linear relationship between the two indices was observed. All data points are shown in the left panel, whereas inoculum dilution rates are indicated in the day‐specific subpanels on the right.
**Figure S17:** Community dissimilarity histograms. Community dissimilarity histograms overlaid with predictions from the mixture model (see Materials and Methods) are shown (these panels present the full results corresponding to Figure 3C). In each panel, the red histogram represents the empirical data, whereas the blue histogram shows the distribution predicted by the mixture model combining multinomial and normal distributions. The overlap between the empirical and simulated distributions is indicated in grey. Each panel displays the community dissimilarity distribution for a specific combination of inoculum source, sampling date, and dilution rate, as indicated at the top of each panel (e.g., “Soil_2_1” denotes a non‐diluted soil‐derived inoculum sampled on Day 2).
**Figure S18:** Quantifying community‐level divergence upon inoculation. (A) Estimated numbers of OTUs lost at inoculation. Given the number of cells introduced and the relative abundance of each OTU in the source community, the numbers of OTUs lost upon inoculation were simulated. Based on 96 draws in the multinomial simulations, boxplots of the numbers of “extinct” OTUs are shown at each dilution rate for each source community. (B) Distributions of pairwise Jaccard dissimilarity among communities. The distribution of pairwise Jaccard dissimilarities among 96 simulated replicate communities at inoculation is shown as purple histograms. Green histograms show the distributions of pairwise Jaccard distances among experimentally observed communities at Day 2. (C) Temporal changes in multimodality calculated using Bray–Curtis dissimilarity. This panel extends Figure 3D by adding estimated multimodality values on Day 0 derived from simulated data. Multimodality values are based on Bray–Curtis dissimilarity, and all other legend details follow Figure 3D. Data on Day 0 were obtained from simulations, whereas data from Day 2 to Day 8 were obtained from experimental observations. (D) Temporal changes in multimodality calculated using Jaccard dissimilarity.
**Figure S19:** Top‐down view of the energy landscapes (Framework 1). The legend follows that of Figure 4A,B.
**Figure S20:** Ecological dynamic regime plots with aligned axis scales. Identical scales are used for the mMDS axes. The legend follows that of Figure 5.
**Figure S21:** Criteria for selecting the representative trajectories of ecological dynamic regimes (soil‐derived microbiomes). The representative trajectories (green arrows) selected with each of the five criteria are shown in the mMDS plot of soil‐derived communities were used to calculate the dynamic dispersion (dDis) of each dynamic regime. The result of the method using the average depth is also shown in Figure 5. The method used to select the representative trajectory is indicated at the top of each panel. Panels are arranged horizontally by dilution rate.
**Figure S22:** Criteria for selecting representative trajectories of ecological dynamic regimes (freshwater‐derived microbiomes). The representative trajectories (green arrows) selected with each of the five criteria are shown in the mMDS plot of freshwater‐derived communities were used to calculate the dynamic dispersion (dDis) of each dynamic regime. The result of the method using the average depth is also shown in Figure 5. The method used to select the representative trajectory is indicated at the top of each panel. Panels are arranged horizontally by dilution rate.
**Figure S23:** Dynamic dispersion metrics based on alternative criteria. In each panel, a representative trajectory selected by each method was used in the calculation of the dynamic dispersion. Except when using the size as the selection criterion, consistent patterns of increasing dynamic dispersion values with increasing dilution rates was observed.
**Figure S24:** Spatial distributions of community structure within the culture plate (soil‐derived communities). To assess whether the spatial arrangement of wells within the plate affects community structure, we conducted a clustering analysis of replicate communities. For each dilution rate of the soil‐derived microbiome, we applied *k*‐means clustering followed by silhouette coefficient analysis to determine the optimal number of clusters. After selecting the number of clusters *k*, we used principal coordinate analysis (PCoA) to reduce the dimensionality of community composition data and visualised the results, with colours representing different clusters. The spatial positions of replicate communities assigned to each cluster are shown on the right panels indicating the spatial organisation within the culture plate.
**Figure S25:** Spatial distributions of community structure within the culture plate (freshwater‐derived communities). To assess whether the spatial arrangement of wells within the plate affects community structure, we conducted a clustering analysis of replicate communities. For each dilution rate of the freshwater‐derived microbiome, we applied *k*‐means clustering followed by silhouette coefficient analysis to determine the optimal number of clusters. After selecting the number of clusters k, we used principal coordinate analysis (PCoA) to reduce the dimensionality of community composition data and visualised the results, with colours representing different clusters. The spatial positions of replicate communities assigned to each cluster are shown on the right panels indicating the spatial organisation within the culture plate.
**Figure S26:** Temporal changes in community composition. Community compositional dissimilarity between consecutive time points is shown for each source community at each dilution rate. Results based on Bray–Curtis and Jaccard dissimilarities are shown in the upper and lower panels, respectively.

**Table S1:** Solutions used in the experiment.
**Table S2:** Coefficient of variation (CV) of expected cell number for each OTU and diltuon rates.
**Table S3:** Outliers in the process quantifying the within‐mode variation of each OTU.
**Table S4:** Raw data of the multimodality and within‐mode variation of each OTU in each inoculum.
**Table S5:** DNA concentration of each inoculum community and predicted DNA copy number.
**Table S6:** Student's *t*‐test of alpha‐diversity and temporal variance.
**Table S7:** Corrlation analysis for Figure 2D,E.
**Table S8:** Detailed information of the multimodality and within‐mode variation at the community level.

## Data Availability

The 16S rRNA gene sequence data are available from the DNA Data Bank of Japan (DDBJ; accession number, Bioproject PRJDB35809). The microbial community data and all the R scripts used for the statistical analyses have been uploaded on Zenodo (DOI: https://doi.org/10.5281/zenodo.19396153).
