# Supplementary material for: Stochastic Forces in Microbial Community Assembly: Founding Community Size Governs Divergent Ecological Trajectories
Source: Ecol Lett. 2026 May 3;29:e70388. doi: 10.1111/ele.70388 (PMC13136789; doi:10.1111/ele.70388)
Supplement: Supplementary file 1 — Figure S1: Rarefaction curve of culture samples and inoculum samples. Relationships between the number of sequencing reads and the number of detected prokaryote ASVs are shown. (A) Rarefaction curves of 50 samples randomly selected from the pool of the samples with 3000 or more sequencing reads are shown in each panel. The data of Day 2 and Day 8 are shown. (B) Rarefaction curves of the soil and freshwater source (inoculum) microbiomes. Figure S2: Estimated number of cells for each OTU in the founding communities (soil inoculum). Based on simulations with multinomial distribution model, the number of cells upon inoculation was estimated for each OTU, and the variability was derived from 96 independent simulation replicates (see Materials and Methods for details). Results for the four inoculum dilution rates are separately shown. Figure S3: Estimated number of cells for each OTU in the founding communities (freshwater inoculum). Based on simulations with multinomial distribution model, the number of cells upon inoculation was estimated for each OTU (see Materials and Methods for details). Results for the four inoculum dilution rates are separately shown. Figure S4: Estimated CV of cell numbers upon inoculation. (A) Coefficients of variation (CVs) in the estimated number of cells for each OTU in the founding communities among replicate communities. For each panel representing a inoculum setting (4 source community type × 4 dilution rates), data points represent individual OTUs. OTUs with higher relative abundance in the inoculum exhibited lower CVs across replicates. (B) Log10‐transformed CVs. Figure S5: OTU‐level multimodality calculated using an alternative metric. To the validate multimodality values calculated with the ACR method (Ameijeiras‐Alonso et al. 2019) (Figure 2D), we performed a supplementary analysis using the HH method (Hartigan and Hartigan 1985). (A) Relationship between multimodality estimates using the two alternative methods. (B) Relationship betw [file ELE-29-0-s001.pdf]

Supporting Information for

Stochastic Forces in Microbial Community Assembly: Founding Community Size Governs Divergent Ecological Trajectories

Ibuki Hayashi[1†], Martina Sánchez-Pinillos[2] and Hirokazu Toju[1, 3†]

[1] Laboratory of Ecosystems and Coevolution, Graduate School of Biostudies, Kyoto University, Kyoto 606-8501, Japan

[2] Centre for Forest Research, Université du Québec à Montréal, H3C 3P8 Montreal, Canada

[2] Center for Living Systems Information Science (CeLiSIS), Graduate School of Biostudies, Kyoto University, Kyoto 606-8501, Japan

[†]**Correspondence:** Ibuki Hayashi (hayashi.ibuki.62z@st.kyoto-u.ac.jp) **or** Hirokazu Toju (toju.hirokazu.4c@kyoto-u.ac.jp).

This PDF file includes:

Supplementary Methods
Supplementary Figures S1–26

Supplementary Information included in a separate file:
Supplementary Tables S1–8

Supplementary Methods

Continuous culture of microbiomes

Whereas "synthetic community" approaches using explicitly defined sets of microbial species or strains have been commonly applied, experiments with field-collected assemblages of diverse taxa are expected to provide more realistic insights into prokaryotic community assembly. We collected two types of field-collected microbiomes as source communities of the experiment. One is sampled from the soil of the A layer (0–10 cm in depth) in the research forest of Center for Ecological Research, Kyoto University, Shiga, Japan (34.972 ºN; 135.958 ºE) on January 30, 2023. The other source microbiome derived from the surface water of a freshwater pond near the Center for Ecological Research (34.974 ºN, 135.967 ºE). After sampling, the soil was sieved with a 4-mm stainless mesh and then 5 g of the sieved soil was mixed in 40 mL sterilized PBS buffer (the detail is shown in Table S1). The freshwater sample was sieved with 20 μm filter and then 20 mL of the filtered water was mixed in 20 mL sterilized 2 × PBS buffer. In the preparation procedures, we added cycloheximide at a concentration of 200 μg/mL to exclude eukaryotes from the source microbiome. The source prokaryote microbiome was cultured at 22 ºC for 48 hours and diluted 10 times to make inoculum solutions for the subsequent experiment.

We then quantified the number of prokaryotic cells in the source communities (inoculum prokaryotic cell suspensions) based on a quantitative DNA sequencing platform as detailed below (see the subsections "DNA metabarcoding" and "Bioinformatics"). To make a gradient of initial community size, the base inoculum sample (×1) was serially diluted for three steps, making ×1/10, ×1/100, and ×1/1000 inocula for each of the soil and freshwater sources (Fig. 1C). We introduced each of the eight inoculum microbiomes (2 sources × 4 dilution rates) into a complete artificial medium with 96 replicates (in total, 8 inoculum settings × 96 replicates = 768 experimental communities). To make the compositions of the media as simple as possible, we used M9 medium with minimal inorganic additives and three types of carbon resources (glucose, leucine, and citrate as detailed in Table S1). In each well of a 240 μL deep-well plate, 10 μL of the inoculum microbiome solution and 190 μL of medium were installed. The 384 deep-well plate was kept shaken at 200 rpm using a plate thermo-shaker BSR-MB100-4A (Bio Medical Sciences Co. Ltd., Tokyo) at 30 ºC for two days. After two-days incubation, 190 μL out of the 200-μL culture medium was sampled from each of the 96 wells and then we pipetted every two days for 8 days. All pipetting manipulations were performed with high precision using a 384-channel automatic pipetting

machine (EDR-384SR, BIOTEC Co. Ltd., Tokyo) placed in a sterilized environment within a laminar flow cabinet. In each sampling event, 190 μL of fresh medium was added to each well so that the total culture volume was kept constant. In total, 3,072 samples (768 communities/day × 4 time points) were collected.

DNA metabarcoding

To extract DNA from each culture sample, 5 μL of the collected aliquot was mixed with 1 μL lysozyme solution [50 mg/ml lysozyme (Sigma), 20 mM Tris-HCl (pH 8.0), 2 mM EDTA] and the mixed solution was incubated at 37 ºC for 2 hours. After adding proteinase K solution [1/30 (v/v) Proteinase K (Takara), 20 mM Tris-HCl (pH 8.0), 2 mM EDTA], the aliquot was incubated at 55 ºC for 3 hours and 95 ºC for 10 min. The solution was then vortexed for 10 minutes to increase DNA yield.

For the samples of the experimental microbiomes, prokaryote 16S rRNA V4 region was PCR-amplified with the forward primer 515f fused with 3–6-mer Ns for improved Illumina sequencing quality (Lundberg *et al.* 2013) and the forward Illumina sequencing primer (5’- TCG TCG GCA GCG TCA GAT GTG TAT AAG AGA CAG- [3–6-mer Ns] – [515f] -3’) and the reverse primer 806rB fused with 3–6-mer Ns and the reverse sequencing primer (5’- GTC TCG TGG GCT CGG AGA TGT GTA TAA GAG ACA G [3–6-mer Ns] - [806rB] -3’) (0.2 μM each). The buffer and polymerase system of KOD One (Toyobo) was used with the temperature profile of 35 cycles at 98 ºC for 10 s, 55 ºC for 5 s, 68 ºC for 1 s. To prevent generation of chimeric sequences, the ramp rate through the thermal cycles was set to 1 ºC/sec (Stevens *et al.* 2013). Illumina sequencing adaptors were then added to respective samples in the supplemental PCR using the forward fusion primers consisting of the P5 Illumina adaptor, 8-mer indexes for sample identification (Hamady *et al.* 2008) and a partial sequence of the sequencing primer (5’- AAT GAT ACG GCG ACC ACC GAG ATC TAC AC - [8-mer index] - TCG TCG GCA GCG TC -3’) and the reverse fusion primers consisting of the P7 adaptor, 8-mer indexes, and a partial sequence of the sequencing primer (5’- CAA GCA GAA GAC GGC ATA CGA GAT - [8-mer index] - GTC TCG TGG GCT CGG -3’). KOD One was used with a temperature profile of 8 cycles at 98 ºC for 10 s, 55 ºC for 5 s, 68 ºC for 5 s (ramp rate = 1 ºC/s). The PCR amplicons of the samples were then pooled after a purification/equalization process with the AMPureXP Kit (Beckman Coulter). Primer dimers, which were shorter than 200 bp, were removed from the pooled library by supplemental

purification with AMPureXP: the ratio of AMPureXP reagent to the pooled library was set to 1 (v/v) in this process. This library was further purified with E-gel SizeSelect 2 (Invitrogen) and then ca. 440-bp DNA fragments was selectively obtained. The sequencing libraries were processed in an Illumina MiSeq sequencer [271 forward (R1) and 31 reverse (R4) cycles; 20% PhiX spike-in].

For the source microbiome sample, the prokaryote 16S rRNA V4 region was also amplified. To estimate concentrations of 16S rRNA genes included in the inoculum, a quantitative amplicon sequencing platform was applied by introducing five "standard DNA" fragments with controlled concentrations to the PCR master mix solution of the first PCR process as detailed elsewhere (Fujita *et al.* 2023). The standard DNAs were used for the *in-silico* calibration of 16S rRNA gene concentrations in the target sample after sequencing as detailed in the previous study (Fujita *et al.* 2023).

Bioinformatics

In total, 66,302,381 sequencing reads were obtained with the Illumina sequencing. The raw sequencing data obtained in the Illumina sequencing were converted into FASTQ files using the program bcl2fastq 1.8.4 distributed by Illumina. The output FASTQ files were then demultiplexed with the program Claident v0.9. 2022.01.26. The sequencing reads were subsequently processed with the program DADA2 (Callahan *et al.* 2016) v.1.18.0 of R 4.2.2 to remove low-quality data. The molecular identification of the obtained amplicon sequence variants (ASVs) was performed based on the naive Bayesian classifier method (Wang *et al.* 2007) with the SILVA v.138.1 database (Quast *et al.* 2013).

In the analysis of the experimental culture samples, the diversity of microbial ASVs saturated at the sequencing depth of 3,000 reads per sample (Fig. S1). Therefore, the sequencing data of the culture samples was rarefied to 3,000 reads per sample with the "rrarefy" function of the R vegan 2.6.6.1 package (Oksanen *et al.* 2025). Of the 3,072 samples (8 inoculum settings × 96 replicates × 4 time points), 3,063 samples with more than 3,000 reads were used in the following pipeline. After screening for the replicate communities for which sequencing data were available for all the four time points, 3,040 samples were subjected to the following statistical analyses. In total, 490 prokaryote ASVs belonging to two kingdoms, 26 classes, 64 orders, 88 families, and 147 genera were detected (Fig. S9). The ASVs were re-clustered into operational taxonomic units (OTUs) with 99% thresholds using

the program VSEARCH v2.15.2 (Rognes *et al.* 2016), yielding 337 OTUs.

The rarefaction curves indicating relationship between the number of sequencing reads and the number of ASVs were drawn using the vegan 2.6.11 package (Oksanen *et al.* 2025) of R 4.5.2. In the sequencing of source (inoculum) microbiome samples, the diversity of microbial ASVs reached plateaus along the axis of the number of sequencing reads (Fig. S1). The data of sequencing read counts were converted to those of DNA concentrations based on the calibration procedure detailed in the "Bioinformatics" and "Estimating initial variation upon inoculation" subsections of the main text.

In the sequencing of experimental culture samples, the diversity of microbial ASVs also reached plateaus along the axis of sequencing read counts (Fig. S1). Given the rarefaction curves, the dataset of experimental culture samples was rarefied to 3,000 reads per sample with the "rrarefy" function of the R vegan package. Of the 3,072 samples (8 inoculum conditions × 96 replicates × 4 time points), 3,063 samples with more than 3,000 reads were used in the following pipeline. After screening for the replicate communities for which sequencing data were available for all the four time points, 3,040 samples were subjected to the following statistical analyses. In total, 490 prokaryote ASVs belonging to two kingdoms, 26 classes, 64 orders, 88 families, and 147 genera were detected. Then, the ASVs were re-clustered into operational taxonomic units (OTUs) with a threshold similarity of 99% using the program VSEARCH v2.15.2 (Rognes *et al.* 2016), yielding, in total, 337 OTUs.

For a simple evaluation of the temporal fluctuation of the community, α-diversity and temporal variability of each community were quantified. Two types of α-diversity indices, OTU richness and Shannon-Wiener diversity, were calculated for each sample. Then, two types of indices, Jaccard dissimilarity and Bray-Curtis dissimilarity between adjacent days, were used to evaluate temporal variability. All metrics were calculated with the vegan package.

Multimodality and within-mode variation in OTU abundance

For each combination of source microbiomes and inoculum dilution rates (inoculum setting), we examined the degree to which the abundance of each prokaryotic OTU varied among replicate community samples at each time point after the inoculation event. Based on the stability landscape concept of community assembly, the among-sample variation in OTU

abundance was quantified sequentially with two types of indices (Fig. 1A). For the first step, the presence of multiple peaks in the histogram of each OTU's abundance across the replicate samples was examined with a "multimodality" index, which give insights into the presence of multiple basins of attraction in the microbiome assembly (Fig. 2B). Next, for each peak identified in the histogram, "within-mode" variation among replicate samples was calculated by assuming fluctuations within each basin of a stability landscape (Fig. 2B). We then examined whether each type of among-replicate variation could increase with increasing initial variation upon inoculation (i.e., stochasticity at the foundation of the replicate communities).

In the calculation of the multimodality, OTUs that occurred in more than ten communities and accounted for at least 0.5% of the total sequencing reads were targeted. In total, a total of 174 OTU frequency data collected across all time points were analyzed. A multimodality test (Ameijeiras-Alonso *et al.* 2019) was then performed to calculate excess mass statistic and p-values for each OTU in each inoculum setting at each time point. This scaled excess mass statistic obtained in the multimodality test was defined as the multimodality of each OTU's abundance. As multimodality calculations with two alternative approaches (Hartigan & Hartigan 1985; Ameijeiras-Alonso *et al.* 2019) yielded qualitatively similar results (Figs. S5–6), we focused on the results based on one of them (Ameijeiras-Alonso *et al.* 2019). The multimodality test was conducted with the R package multimode 1.5 (Ameijeiras-Alonso *et al.* 2019) by setting the number of replicates to 3,000. The p-values indicating the presence/absence of multiple peaks (modes) were adjusted for multiple comparisons at each time point based on false discovery rate (FDR) to obtain q-values.

We next calculated "within-mode" variation, which is expected to reflect the level of fluctuation within each basin of attraction in the post-inoculation processes (Fig. 1A). To calculate the additional measure of among-sample variation, we identified the number of peaks in the histograms of OTU abundance. Specifically, we classified OTUs with unimodal and multimodal distributions by applying the abovementioned multimodality test with a threshold q-value (significance level) of 0.05 in each inoculum setting at each time point. The frequency of OTU abundance within each peak (mode) was then assumed to follow a mixture model of a binomial distribution and a standard normal distribution (Fig 2B). In principle, the parameters of the binomial distribution were automatically obtained as the number of trials (i.e., the rarefied number of sequencing reads per sample = 3,000) and the probability of observations (i.e., the relative abundance of a target OTU in a target community sample)

based on the assumption of Bernoulli trial. Therefore, the standard deviation within the normal distribution part in the mixture model was used as a measure of among-sample variation caused after the inoculation event.

The fitting to the mixture model was conducted by optimizing following likelihood function:

$$L\left(\sigma_i^{\Omega_{I,c,t}}; X_i^{\Omega_{I,c,t}} = \left\{x_{i,1}^{\Omega_{I,c,t}}, \cdots, x_{i,m}^{\Omega_{I,c,t}}, \cdots, x_{i,M}^{\Omega_{I,c,t}}\right\}\right) = \sum_{m=1}^{M} \log\left(\sum_{x_i^{\Omega_{I,c,t}}=0}^{R} \binom{R}{x_i^{\Omega_{I,c,t}}} p_i^{x_i^{\Omega_{I,c,t}}} (1 - p_i)^{R - x_i^{\Omega_{I,c,t}}} \mathcal{N}\left(0, \sigma_i^{\Omega_{I,c,t}}\right)\right),$$

where $\sigma_i^{\Omega_{I,c,t}}$ was the standard deviation of a standard normal distribution, $X_i^{\Omega_{I,c,t}}$ indicated the sequencing read numbers of OTU i in each subset data Ω [subscripts I, c, and t denoted the type of inoculum communities (soil or freshwater source communities), the dilution rate of inoculum community, and time point, respectively], and M was the number of replicate communities. In the right hand of the equation, R denoted the threshold number of sequencing reads (3,000 reads/sample in the rarefaction step of this study), p_i was the relative abundance of OTU i of each subset Ω, and $\mathcal{N}(0, \sigma)$ indicated a standard normal distribution whose standard deviation was σ. The standard deviation ($\sigma_i^{\Omega_{I,c,t}}$) was estimated by minimizing the likelihood function using the "optimize" function in R. To gain reliable estimates, we conducted the model fitting ten times and then used the mean value of the estimates as "within-mode" variation for each peak of a target OTU in each inoculum setting at each time point. For each of the OTUs with multiple peaks, a mean value of the estimates was calculated. The within-mode variation estimate was then scaled by dividing it by the mean abundance of each OTU. Since the estimates of standard deviations were highly influenced by the presence of outliers, five of the largest and five of the smallest outliers were removed (see Table S3 for the information of the removed samples).

For the analysis of the OTUs with multimodal distributions, the data were divided into an optimal number of clusters. Specifically, after applying k-means clustering, the optimal number of clusters was inferred based on the silhouette coefficients calculated with the cluster 2.1.6 package (Maechler *et al.* 2024) of R. The clusters that contained six or more replicate samples were used in the fitting to the mixture model: outliers were not removed in the analysis for the multimodal cases.

Community-scale differentiation among replicates

By extending the statistical approach applied at the OTU-level analysis, we next developed a method for quantifying community-level differentiation of community structure. Instead of the distribution of each OTU's abundance across samples (Fig. 2B), we focused on the distribution of pairwise dissimilarities (Bray-Curtis β-diversity) between replicate samples (Fig. 3A–B). The multimodality of pairwise community dissimilarity distributions was calculated for each inoculum setting at each time point to evaluate the extent to which replicate communities diverged into multiple basins of community structure. We performed additional analyses with Jensen–Shannon divergence and Hellinger distance: qualitatively similar results with those based on Bray–Curtis dissimilarity were obtained (Fig. S7).

Likewise, variation in community structure within each basin of the stability landscape (Fig. 1A) was inferred by quantifying within-mode variation in the distribution of pairwise community dissimilarity. For each inoculum setting at each time point, we checked whether multiple peaks existed within the histogram of pairwise community dissimilarity. In general, the presence of multiple peaks within the distribution of pairwise dissimilarities indicates that the focal community dataset includes multiple clusters (groups) of data points (Hayashi *et al.* 2024). In our analysis, if the presence of multiple groups was supported in a multimodality test (threshold q-value = 0.05; Fig. 3B), we performed a k-means clustering analysis followed by a silhouette coefficient analysis to split the replicate communities into groups.

For each split dataset, the community compositions of replicate samples were assumed to follow the following mixture model of a multinomial distribution and a standard normal distribution:

$$C_{\mathrm{sim}} \sim \left(\frac{R!}{x_1! \cdots x_n!} {p_1}^{x_1} \cdots {p_n}^{x_n}\right) + \mathcal{N}(0, \Sigma),$$

where C_{sim} was the community matrix derived from the mixture model. In the multinomial part [$\left(\frac{R!}{x_1! \cdots x_n!} {p_1}^{x_1} \cdots {p_n}^{x_n}\right)$], R was the threshold number of sequencing reads (= 3,000 reads/sample), x_i is the random variable of multinomial distribution corresponding to the read count of OTU i (hence, $\sum_1^n x_i$ = 3,000), and p_i was a parameter of multinomial distribution corresponding to the occurrence probability of OTU i. In the part of normal distribution [$\mathcal{N}(0, \Sigma)$], standard deviation of each OTU was assumed to be proportional to its occurrence probability (p_i) as follows:

$$\Sigma = \mathrm{diag}((sp_1)^2, \cdots, (sp_n)^2),$$

where s is a constant representing the variability of community structure among replicate samples. In the simulation with the mixture model, the occurrence probability of each OTU (p_i) was estimated from the abundance information of each OTU within the original data matrix by assuming a Dirichlet distribution with the "rdirichlet" function in the R package MCMCpack 1.7.1 (Martin *et al.* 2024). The most likely variability constant (s) in the normal distribution part were determined by minimizing the difference between the distribution derived from the actual data and the distribution derived from the mixture model as follows:

$$F(C_{\mathrm{sim}} | C_{\mathrm{obs}}) = \left(E\left(D(C_{\mathrm{obs}})\right) - E\left(D(C_{\mathrm{sim}})\right)\right)^2,$$

where C_{sim} was the community matrix generated by the mixture model and C_{obs} was the community matrix of the observed data. In the right hand of the equation, $E\left(D(C_{\mathrm{obs}})\right)$ represented an empirical cumulative distribution function (eCDF) of Bray-Curtis dissimilarity among replicate samples in the observed data matrix C_{obs}, while $E\left(D(C_{\mathrm{sim}})\right)$ was an eCDF of Bray-Curtis dissimilarity in the simulated data matrix C_{sim}. This solving process was conducted one hundred times, among which the run with the smallest $F(C_{\mathrm{sim}} | C_{\mathrm{obs}})$ was used for gaining a reliable estimate of the constant (s) representing community variability within a basin of community structure.

Energy landscape analysis

To infer the stability landscape architecture of the experimental microbiomes, we applied the statistical framework of energy landscape analysis, which captures complex systems' behavior based on the Ising model of statistical physics (Suzuki *et al.* 2021; Masuda *et al.* 2025). The statistical framework has been applied to neuroscience (Watanabe *et al.* 2014) and ecology (Suzuki *et al.* 2021; Fujita *et al.* 2023; Kadoya *et al.* 2025), giving insights into the multi-stable states of systems. When applied to ecological community data (tutorials and R codes of energy landscape analyses are available at https://github.com/kecosz/rELA), the probability of observing a specific community state [$P\left(\vec{\sigma}^{(k)}\right)$] is expressed as:

$$P\left(\vec{\sigma}^{(k)}\right) = e^{-E\left(\vec{\sigma}^{(k)}\right)} / Z,$$

$$Z = \sum_{i=1}^{2^S} e^{-E\left(\vec{\sigma}^{(k)}\right)},$$

where $\vec{\sigma}^{(k)} = \left(\sigma_1{}^{(k)}, \sigma_2{}^{(k)}, \dots, \sigma_S{}^{(k)}\right)$ is a community state vector of *k*-th sample and S is the total number of the taxa examined (e.g., the number of OTUs, species, genera, or families in the input data). Within the community state vector, $\sigma_i{}^{(k)}$ is a binary variable that indicates presence (1) or absence (0) of taxon *i*: i.e., there are a total of 2^S community states. When input community matrix is defined, the $E\left(\vec{\sigma}^{(k)}\right)$ part of the equation is fitted based on an extended pairwise maximum entropy model defined as follows:

$$E\left(\vec{\sigma}^{(k)}\right) = -\sum_{i=1}^{S} h_i \, \vec{\sigma}^{(k)}{}_i - \sum_{i=1}^{S} \sum_{j=1, i \neq j}^{S} J_{ij} \, \vec{\sigma}^{(k)}{}_i \, \vec{\sigma}^{(k)}{}_j / 2,$$

where h_i represents the net effect of implicit abiotic factors, by which i-th taxon is more likely to present ($h_i > 0$) or not ($h_i < 0$), and J_{ij} represents the pattern of co-occurrence between *i*-th and *j*-th taxa. Since the logarithm of the probability of a community state is inversely proportional to $E\left(\vec{\sigma}^{(k)}\right)$, a community state having lower *E* is observed more frequently. Note that the "energy" metric (*E*) does not correspond in any way to the physical form of energy: it is specifically defined with the above equation in energy landscape analysis (Suzuki *et al.* 2021). Based on the statistical model, community states that show lower $E\left(\vec{\sigma}^{(k)}\right)$ values than all adjacent community states within an assembly graph are explored, inferred as attractors of community dynamics (Suzuki *et al.* 2021).

In applying energy landscape analysis to our dataset, the rELA 0.81 library (https://github.com/kecosz/rELA) of R was used. The original community data were converted into binary input data using the following read count threshold: OTUs that accounted for at least 4.32×10^{-4} % of the total reads ($=$ 5 reads / (384 samples $\times$ 3000 reads)) and appeared in at least 5 out of 384 samples started with the same inoculum condition were used as input data.

Ecological dynamic regimes

The dynamic dispersion (dDis) quantifies the average dissimilarity of each trajectory from the representative trajectory. The dynamic dispersion index is calculated as follows:

$$\mathrm{dDis} = \frac{\sum_{i=1}^{m} d_{i\alpha}}{m},$$

where $d_{i\alpha}$ represents dissimilarity between a trajectory i and a reference trajectory α, and m is the number of observed trajectories (i.e., the number of replicate time series). When

quantifying the dispersion of community dynamics from the overall direction of assembly, we selected the representative trajectory with the highest resolution of state space, based on the average depth of representative trajectories, as the reference trajectory. Alternatively, a reference trajectory could be selected based on other criteria, such as the one with the largest number of segments (selection by size), the one with the smallest average link dissimilarity connecting segments within the trajectory (selection by average link), or the one whose constituent segments best represent all segments in the EDR on average (selection by average density). To evaluate these alternatives, we conducted calculations using (i) the representative trajectory that performed best according to each of the size, average link dissimilarities, and average density metrics, and (ii) the trajectory with the lowest total rank across all four metrics: average depth, size, average link, and average density. The overall trends did not differ substantially from those obtained using average depth as the selection criterion. The dynamic dispersion score varies from 0 (when all trajectories are identical to the representative trajectory) to 1 (when replicate communities take completely different trajectories of community dynamics).

The dynamic beta-diversity (dBD) quantifies the average dissimilarity between community trajectories as follows:

$$\mathrm{dBD} = \frac{\sum_{i=1}^{m-1}\sum_{j=i+1}^{m} {d_{ij}}^2}{m(m-1)},$$

where d_{ij} denotes dissimilarity between trajectories i and j. Like the dynamic dispersion, the dynamic beta-diversity varies from 0 (completely identical dynamic among replicates) and 1 (complete differentiation of temporal community dynamics).

The dynamic evenness (dEve) measures the continuity of trajectory variation within the dynamic regime as follows:

$$\mathrm{dEve} = \frac{\sum_{c=1}^{m-1} \min\left(\frac{d_{ij}}{\sum_{c=1}^{m-1} d_{ij}}, \frac{1}{m-1}\right) - \frac{1}{m-1}}{1 - \frac{1}{m-1}},$$

where c is the edges of a minimum spanning tree constructed from the set of trajectories forming a dynamic regime. In contrast to the dynamic dispersion and dynamic beta-diversity, a higher value of dynamic evenness indicates a lower level of divergence among the time series of replicate communities. It ranges from 0 (when many subclusters of trajectories exist) and 1 (when all trajectories are evenly distributed).

The three EDR metrics were calculated for each of the eight inoculum settings (2 source communities × 4 dilution rates) defining the dynamic regimes using the R ecoregime 0.2.0 package (Sánchez-Pinillos *et al.* 2023). In addition, the representative trajectories of temporal community dynamics were inferred with the ecoregime package (the minSegs parameter in the 'retra_edr' function was set as 5). For each inoculum setting, the representative trajectories are shown on a two-dimensional surface of community compositions, which was defined based on metric multidimensional scaling (mMDS) using Bray-Curtis dissimilarities (Fig. 5).

References for the Supplementary Methods

Ameijeiras-Alonso, J., Crujeiras, R.M. & Rodríguez-Casal, A. (2019). Mode testing, critical bandwidth and excess mass. *TEST*, 28, 900–919.

Callahan, B.J., McMurdie, P.J., Rosen, M.J., Han, A.W., Johnson, A.J.A. & Holmes, S.P. (2016). DADA2: High-resolution sample inference from Illumina amplicon data. *Nat Methods*, 13, 581–583.

Fujita, H., Ushio, M., Suzuki, K., Abe, M.S., Yamamichi, M., Iwayama, K., *et al.* (2023). Alternative stable states, nonlinear behavior, and predictability of microbiome dynamics. *Microbiome*, 11, 63.

Hamady, M., Walker, J.J., Harris, J.K., Gold, N.J. & Knight, R. (2008). Error-correcting barcoded primers for pyrosequencing hundreds of samples in multiplex. *Nat Methods*, 5, 235–237.

Hartigan, J.A. & Hartigan, P.M. (1985). The Dip Test of Unimodality. *The Annals of Statistics*.

Hayashi, I., Fujita, H. & Toju, H. (2024). Deterministic and stochastic processes generating alternative states of microbiomes. *ISME Communications*, 4, ycae007.

Kadoya, T., Suzuki, K. & Terui, A. (2025). Linking energetic instability to compositional changes in biological communities. *Proceedings of the National Academy of Sciences*, 122, e2422701122.

Lundberg, D.S., Yourstone, S., Mieczkowski, P., Jones, C.D. & Dangl, J.L. (2013). Practical

innovations for high-throughput amplicon sequencing. *Nat Methods*, 10, 999–1002.

Maechler, M., original), P.R. (Fortran, original), A.S. (S, original), M.H. (S, Hornik [trl, K., maintenance(1999-2000)), ctb] (port to R., *et al.* (2024). cluster: “Finding Groups in Data”: Cluster Analysis Extended Rousseeuw et al.

Martin, A.D., Quinn, K.M., Park, J.H., Vieilledent, G., Malecki, M., Blackwell, M., *et al.* (2024). MCMCpack: Markov Chain Monte Carlo (MCMC) Package.

Masuda, N., Islam, S., Aung, S.T. & Watanabe, T. (2025). Energy landscape analysis based on the Ising model: Tutorial review. *PLOS Complex Systems*, 2, e0000039.

Oksanen, J., Simpson, G.L., Blanchet, F.G., Kindt, R., Legendre, P., Minchin, P.R., *et al.* (2025). vegan: Community Ecology Package.

Quast, C., Pruesse, E., Yilmaz, P., Gerken, J., Schweer, T., Yarza, P., *et al.* (2013). The SILVA ribosomal RNA gene database project: improved data processing and web-based tools. *Nucleic Acids Research*, 41, D590–D596.

Rognes, T., Flouri, T., Nichols, B., Quince, C. & Mahé, F. (2016). VSEARCH: a versatile open source tool for metagenomics. *PeerJ*, 4, e2584.

Sánchez-Pinillos, M., Kéfi, S., De Cáceres, M. & Dakos, V. (2023). Ecological dynamic regimes: Identification, characterization, and comparison. *Ecological Monographs*, 93, e1589.

Stevens, J.L., Jackson, R.L. & Olson, J.B. (2013). Slowing PCR ramp speed reduces chimera formation from environmental samples. *Journal of Microbiological Methods*, 93, 203–205.

Suzuki, K., Nakaoka, S., Fukuda, S. & Masuya, H. (2021). Energy landscape analysis elucidates the multistability of ecological communities across environmental gradients. *Ecological Monographs*, 91, e01469.

Wang, Q., Garrity, G.M., Tiedje, J.M. & Cole, J.R. (2007). Naïve Bayesian Classifier for Rapid Assignment of rRNA Sequences into the New Bacterial Taxonomy. *Applied and Environmental Microbiology*, 73, 5261–5267.

Watanabe, T., Masuda, N., Megumi, F., Kanai, R. & Rees, G. (2014). Energy landscape and dynamics of brain activity during human bistable perception. *Nat Commun*, 5, 4765.

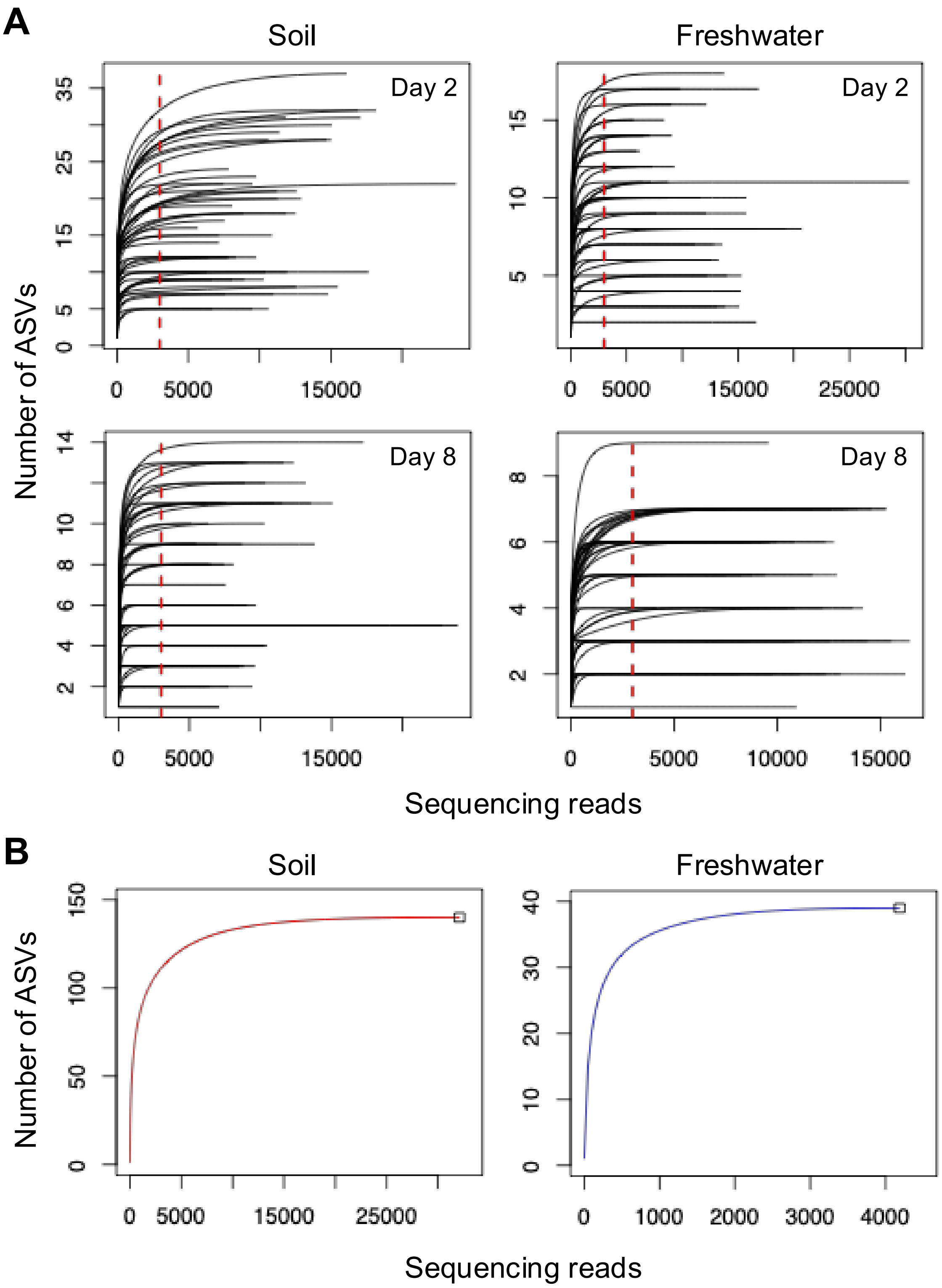

Figure S1 | Rarefaction curve of culture samples and inoculum samples. Relationships between the number of sequencing reads and the number of detected prokaryote ASVs are shown. (A) Rarefaction curves of 50 samples randomly selected from the pool of the samples

with 3,000 or more sequencing reads are shown in each panel. The data of Day 2 and Day 8 are shown. (B) Rarefaction curves of the soil and freshwater source (inoculum) microbiomes.

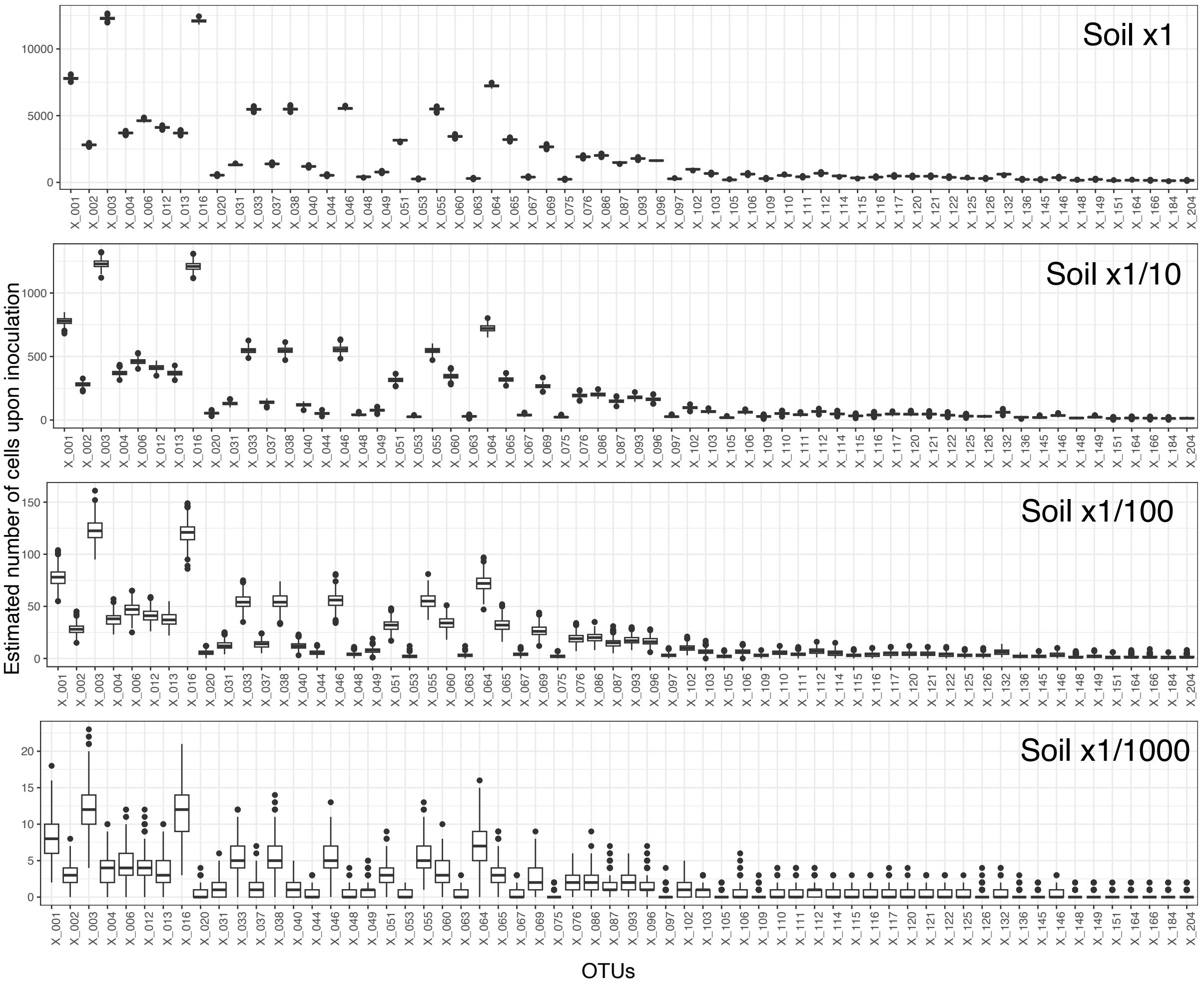

Figure S2 | Estimated number of cells for each OTU in the founding communities (soil inoculum). Based on simulations with multinomial distribution model, the number of cells upon inoculation was estimated for each OTU, and the variability was derived from 96 independent simulation replicates (see Materials and Methods for details). Results for the four inoculum dilution rates are separately shown.

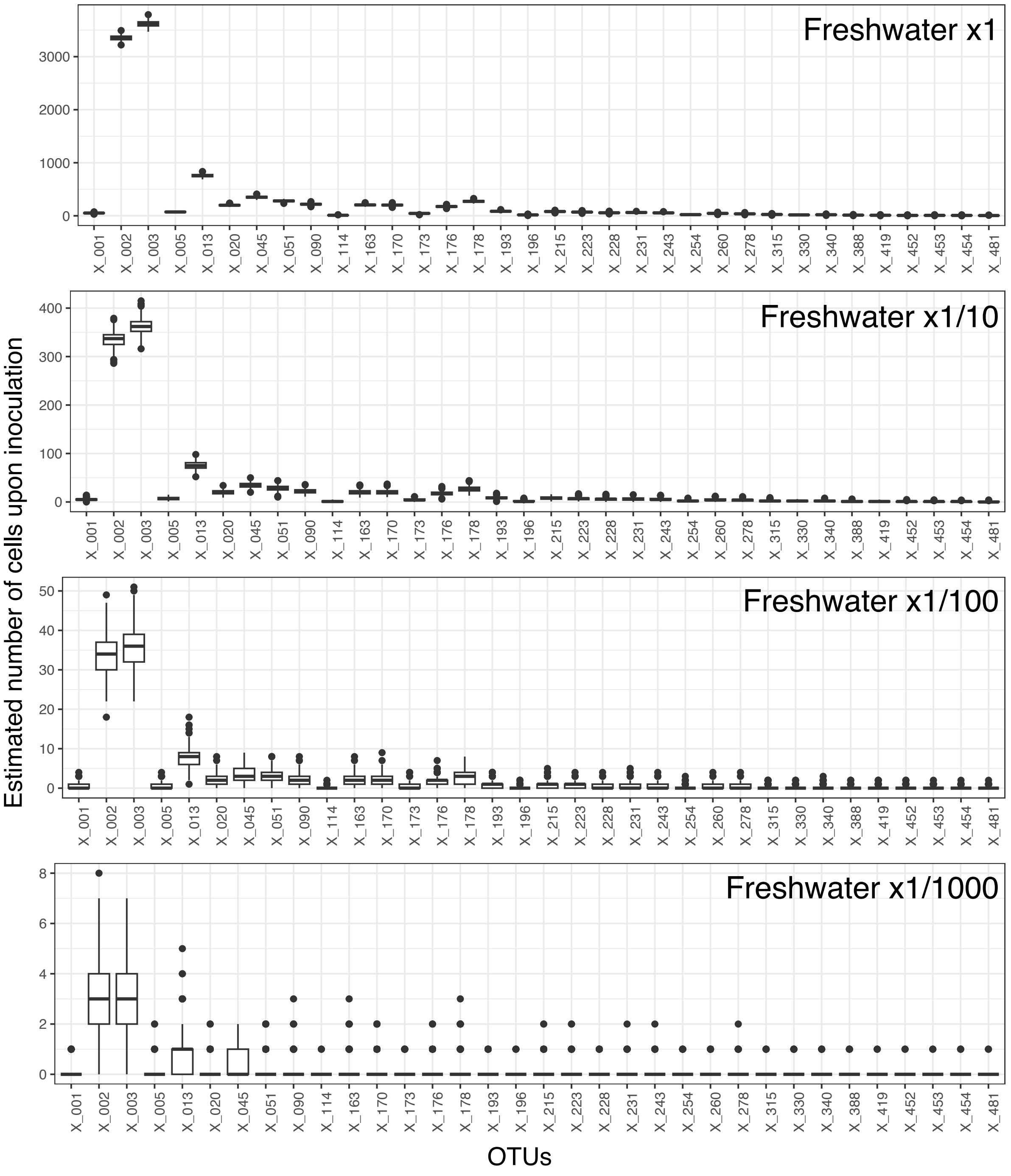

Figure S3 | Estimated number of cells for each OTU in the founding communities (freshwater inoculum). Based on simulations with multinomial distribution model, the number of cells upon inoculation was estimated for each OTU (see Materials and Methods for details). Results for the four inoculum dilution rates are separately shown.

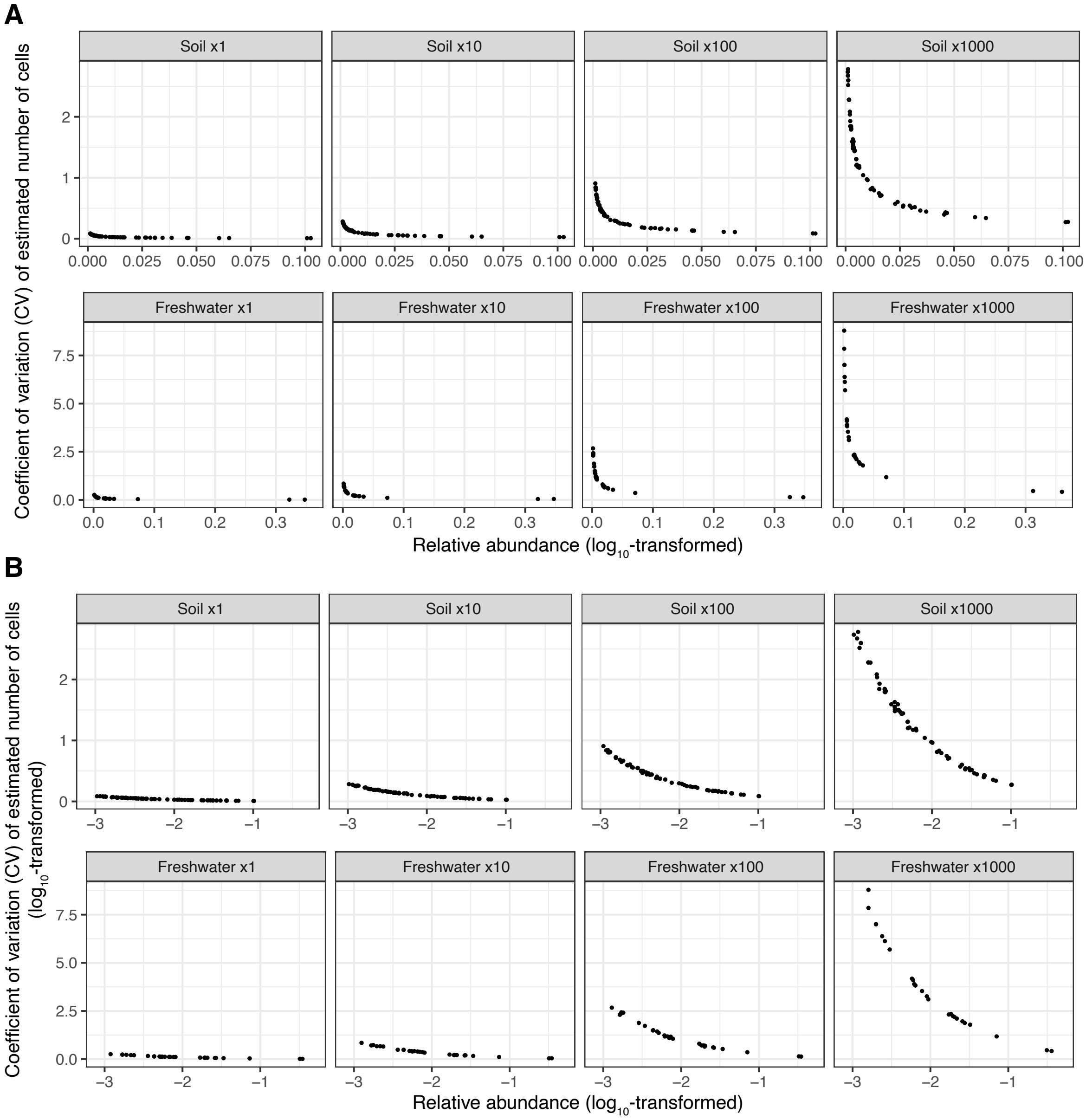

Figure S4 | Estimated CV of cell numbers upon inoculation. (A) Coefficients of variation (CVs) in the estimated number of cells for each OTU in the founding communities among replicate communities. For each panel representing a inoculum setting (4 source community type × 4 dilution rates), data points represent individual OTUs. OTUs with higher relative abundance in the inoculum exhibited lower CVs across replicates. (B) Log_{10}-transformed CVs.

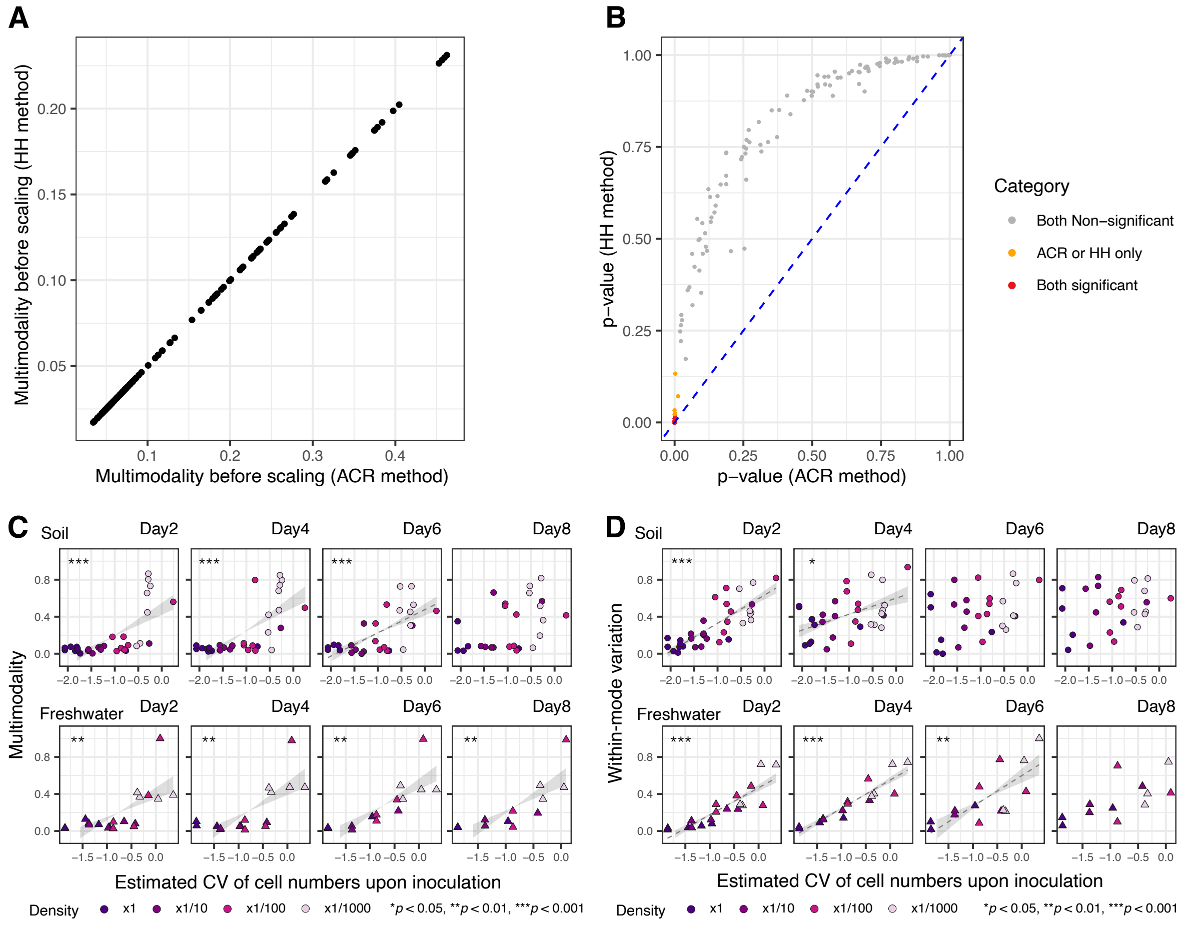

Figure S5 | OTU-level multimodality calculated using an alternative metric. To the validate multimodality values calculated with the ACR method (Ameijeiras-Alonso *et al.* 2019) (Fig. 2D), we performed a supplementary analysis using the HH method (Hartigan & Hartigan 1985). (A) Relationship between multimodality estimates using the two alternative methods. (B) Relationship between the *p*-values of multimodality tests obtained from the two alternative methods. (C) Relationship between initial stochasticity and multimodality (results based on HH method). By targeting the OTUs commonly observed across experimental replicates (the section "Multimodality and within-mode variation in OTU abundance" in Supplementary Methods), relationship between the estimated CV of cell numbers upon inoculation and multimodality was examined for each source microbiome type (soil or freshwater) at each time point. The multimodality estimates are scaled from 0 to 1 across the panels. Lines represent significant linear regressions (FDR < 0.05). (D) Relationship between estimated CV of cell numbers upon inoculation and within-mode variation. Note that multimodality test performed prior to the calculation of within-mode variation was more stringent in the HH method than the ACR method (panel B).

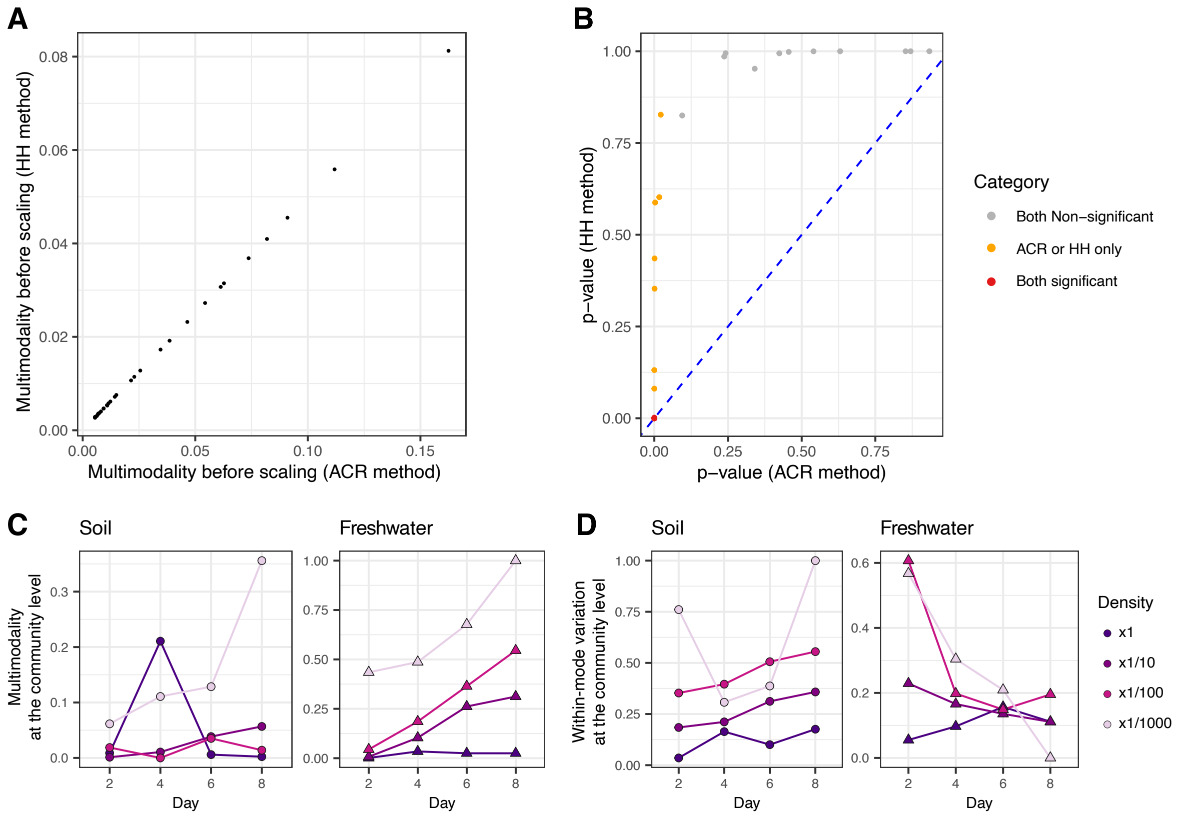

Figure S6 | Multimodality at the community level calculated using an alternative metric. To the validate multimodality values calculated with the ACR method (Ameijeiras-Alonso *et al.* 2019) (Fig. 3D), we performed a supplementary analysis using the HH method (Hartigan & Hartigan 1985). (A) Relationship between the *p*-values of multimodality tests obtained from the two alternative methods. (B) Relationship between the *p*-values of multimodality tests obtained from the two alternative methods. (C) Temporal changes in multimodality (results based on HH method). For each combination of source microbiome type (soil or freshwater) and inoculum dilution rates, the temporal trends of community-scale multimodality are shown. Multimodality estimates are scaled from 0 to 1 across the panels. (D) Temporal changes in within-mode variation. The community-scale estimates of within-mode variation are scaled from 0 to 1 across the panels. Note that multimodality test performed prior to the calculation of within-mode variation was more stringent in the HH method than the ACR method (panel B).

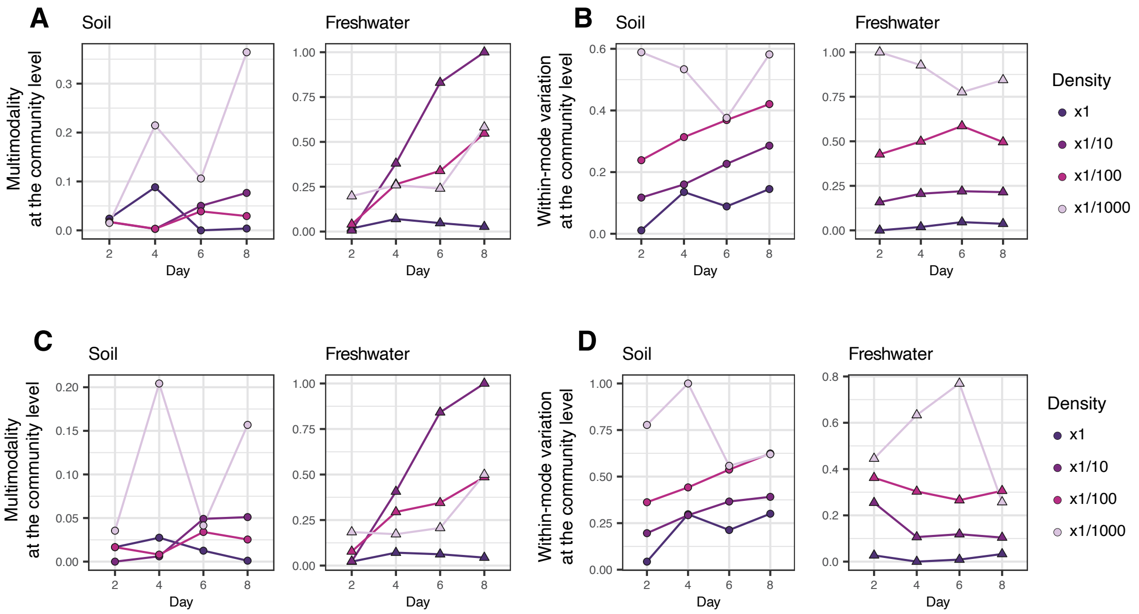

Figure S7 | Community-scale multimodality and within-mode variation calculated with alternative community dissimilarity metrices. To confirm the results based on Bray-Curtis dissimilarity (Figure 3), we calculated multimodality and within-mode variation based, respectively, on Jensen–Shannon distance and Hellinger distance. (A) Multimodality based on Jensen-Shannon distance. (B) Within-mode variation based on Jensen-Shannon distance. (C) Multimodality based on Hellinger distance. (D) Within-mode variation based on Hellinger distance.

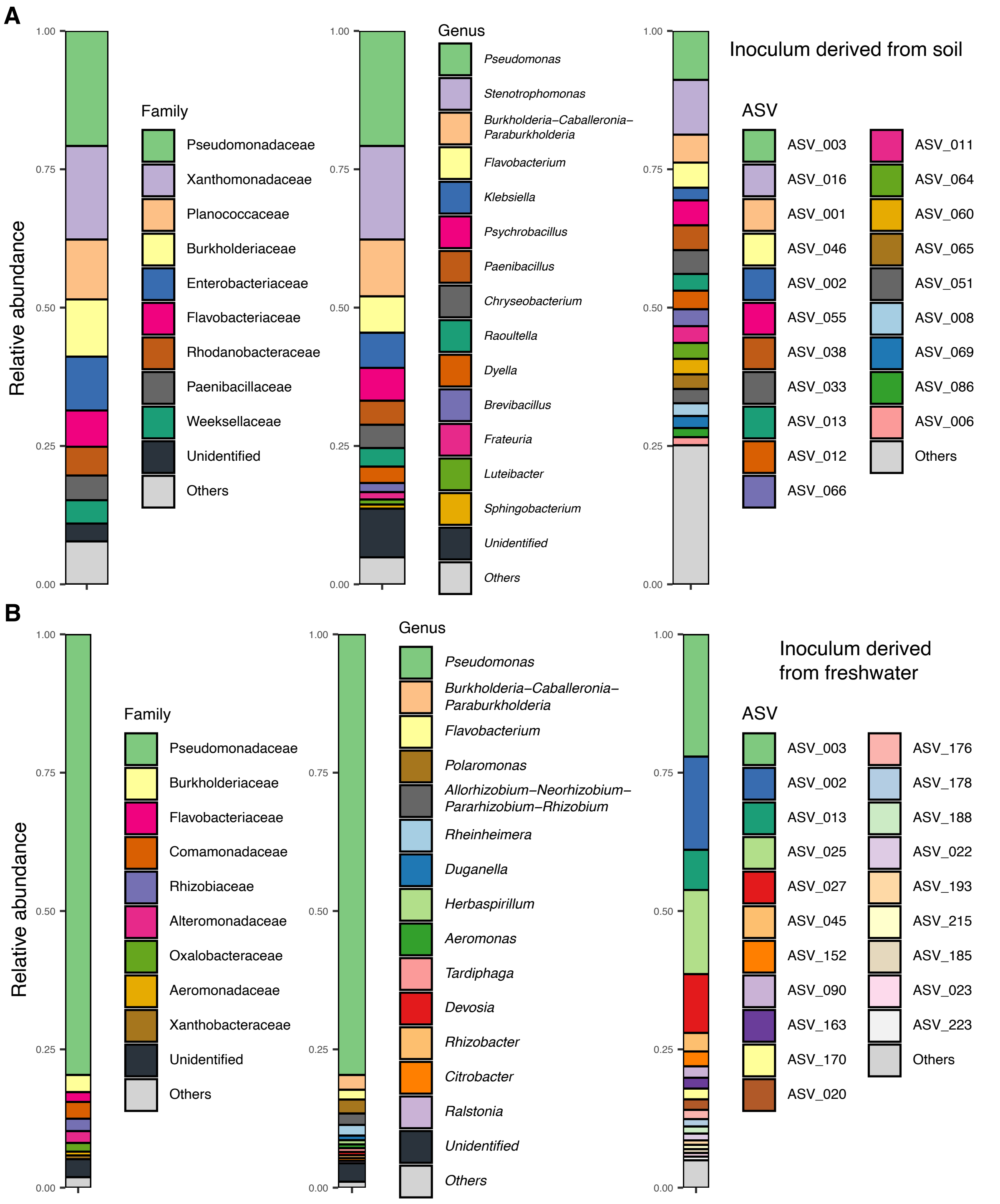

Figure S8 | Taxonomic compositions of soil and freshwater source (inoculum) microbiomes. For each of the soil (A) and freshwater (B) source microbiomes, community compositions are respectively shown at the family, genus, and ASV levels.

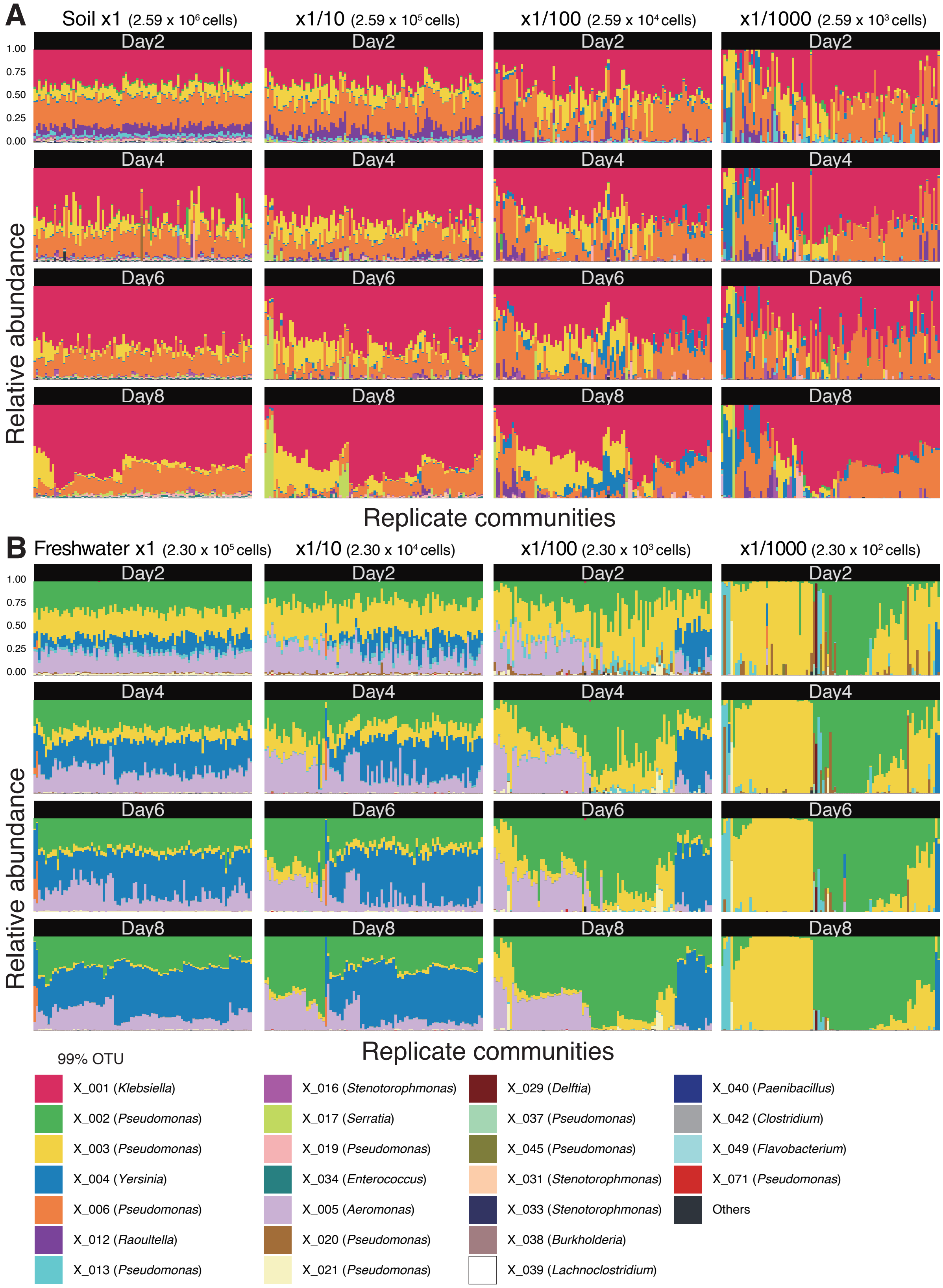

Figure S9 | Overview of among-replicate variation in community structure. (A) Experiment with the soil inoculum microbiome. Temporal changes in the 99% OTU-level community

compositions (relative abundance) are shown. For each inoculum dilution rate, replicate communities were ordered based on the results of unweighted pair group method with arithmetic mean (UPGMA) analyses performed at Day 8. The founding community size estimated based on quantitative amplicon sequencing is shown for each inoculum setting. (B) Experiment with the freshwater inoculum microbiome. The experimental microbiomes deriving from the soil source community were constituted mainly by the five genera, *Klebsiella*, *Pseudomonas*, *Raoultella*, *Stenotrophomonas*, and *Serratia*, while those deriving from the freshwater source community were dominated by the three genera, *Pseudomonas*, *Yersinia*, and *Aeromonas*. For both types of inoculum communities, the OTU-level compositions of experimental communities varied more conspicuously among replicate communities at higher dilution rates. Such elevation of among-sample variation in community compositions with increasing dilution rate was observed as well at the ASV- and genus-level analyses (Figs. S10–11).

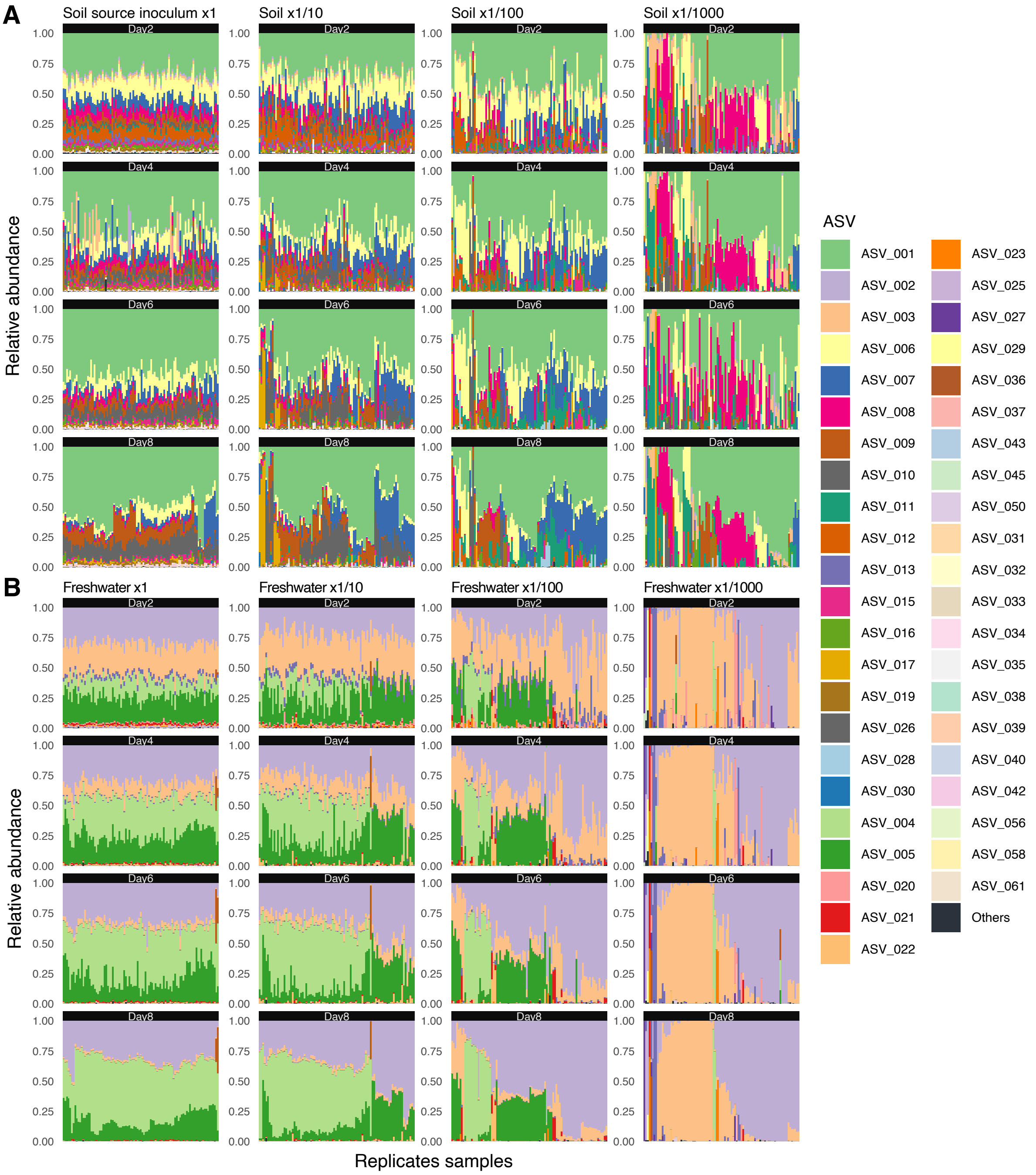

Figure S10 | Overview of among-replicate variation in community structure (ASV-level results). (A) Experiment with soil inoculum microbiome. Temporal changes in the ASV-level community compositions (relative abundance) are shown. For each inoculum dilution rate, replicate communities were ordered based on the results of unweighted pair group method with arithmetic mean (UPGMA) analyses performed on Day 8. (B) Experiment with freshwater inoculum microbiome.

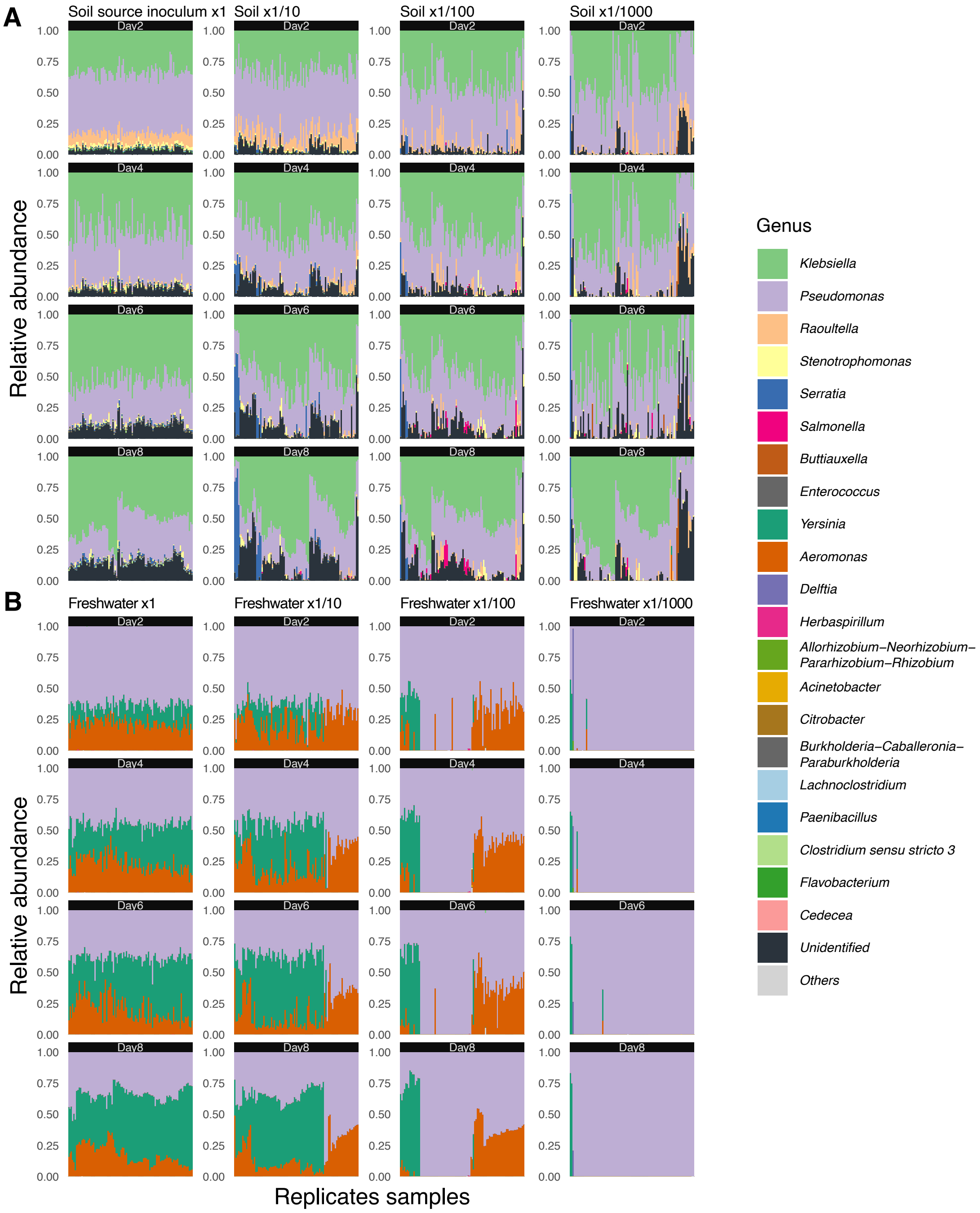

Figure S11 | Overview of among-replicate variation in community structure (genus-level results). (A) Experiment with soil inoculum microbiome. Temporal changes in the genus-level community compositions (relative abundance) are shown. For each inoculum dilution rate, replicate communities were ordered based on the results of unweighted pair group method with arithmetic mean (UPGMA) analyses performed on Day 8. (B) Experiment with freshwater inoculum microbiome.

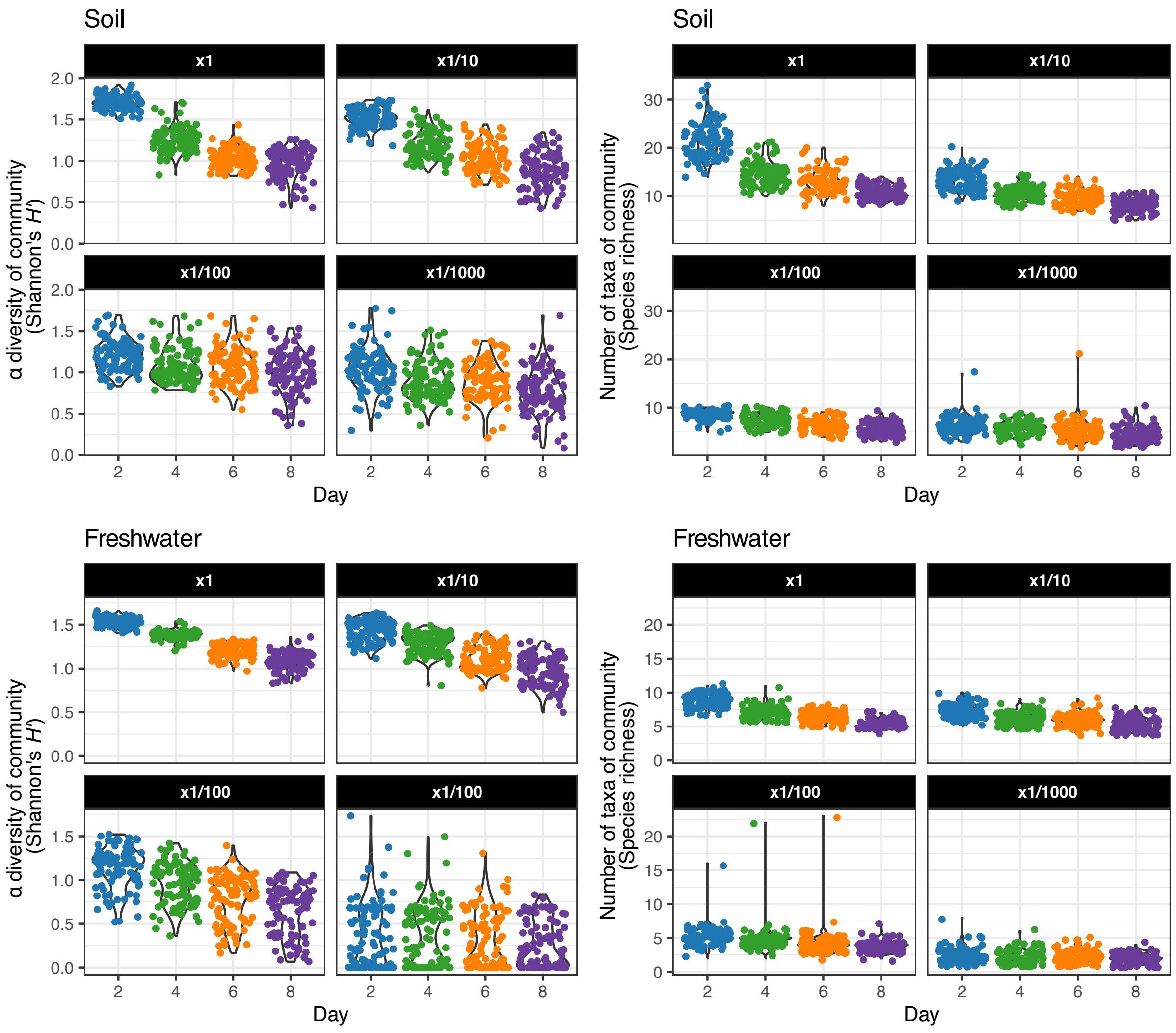

Figure S12 | Alpha diversity of the experimental microbiomes. Temporal changes in the Shannon's diversity index (left) and richness (right) of 99% OTUs are shown for the soil (top) and freshwater (bottom) experimental microbiomes. The results of Student's *t*-tests comparing each pair of time points are presented in Table S6. In each combination of source microbiomes and inoculum dilution rates, alpha-diversity decreased through time in the microbiome experiment, and the average of Shannon's diversity of the OTUs was 0.902 (Shannon effective number = 2.54) for the soil- and 0.743 (Shannon effective number = 2.25) for the freshwater-derived communities at Day 8.

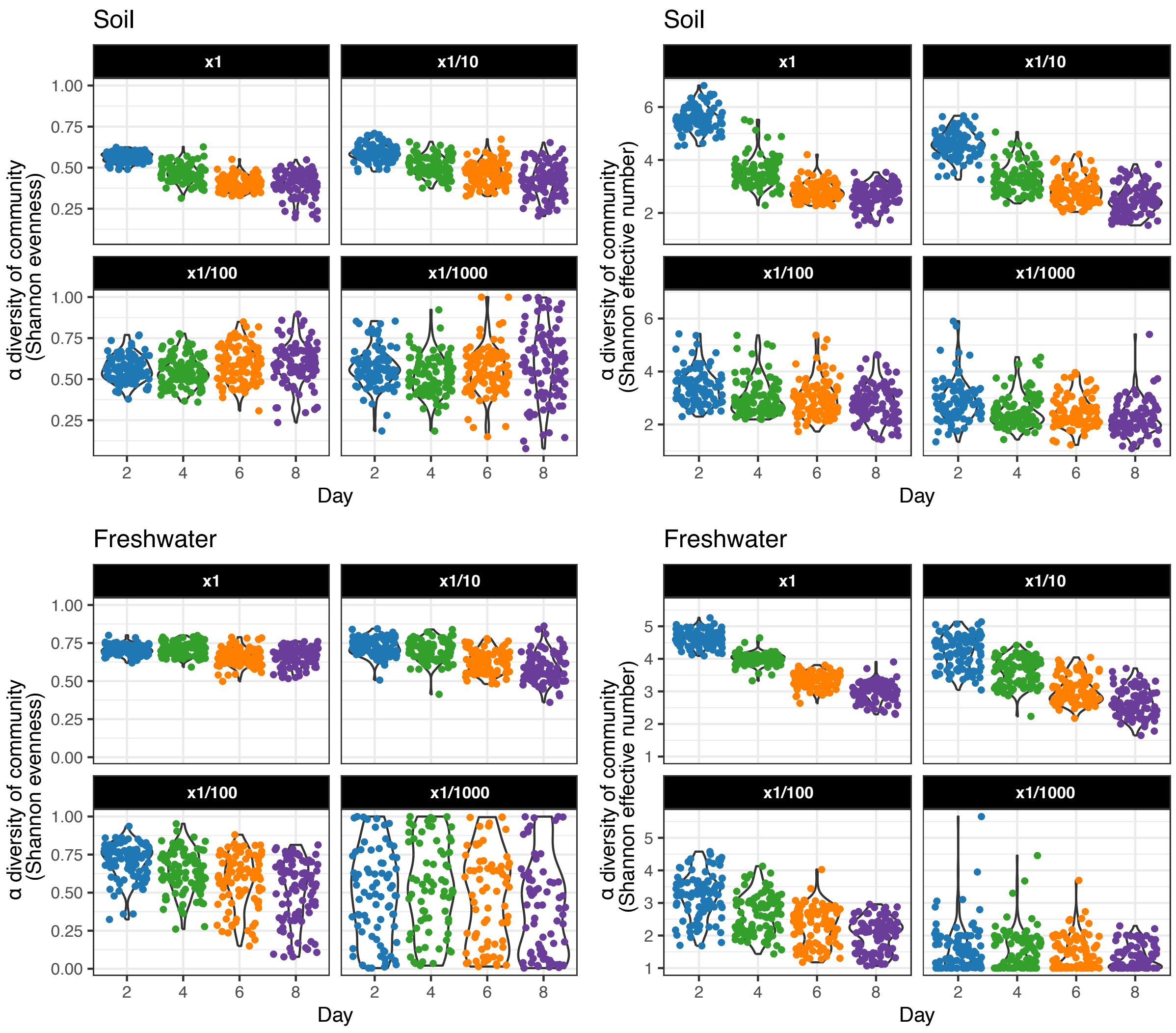

Figure S13 | Alpha diversity of the experimental microbiomes. Temporal changes in the Shannon evenness (left) and Shannon effective number (right) of 99% OTUs are shown for the soil (top) and freshwater (bottom) experimental microbiome.

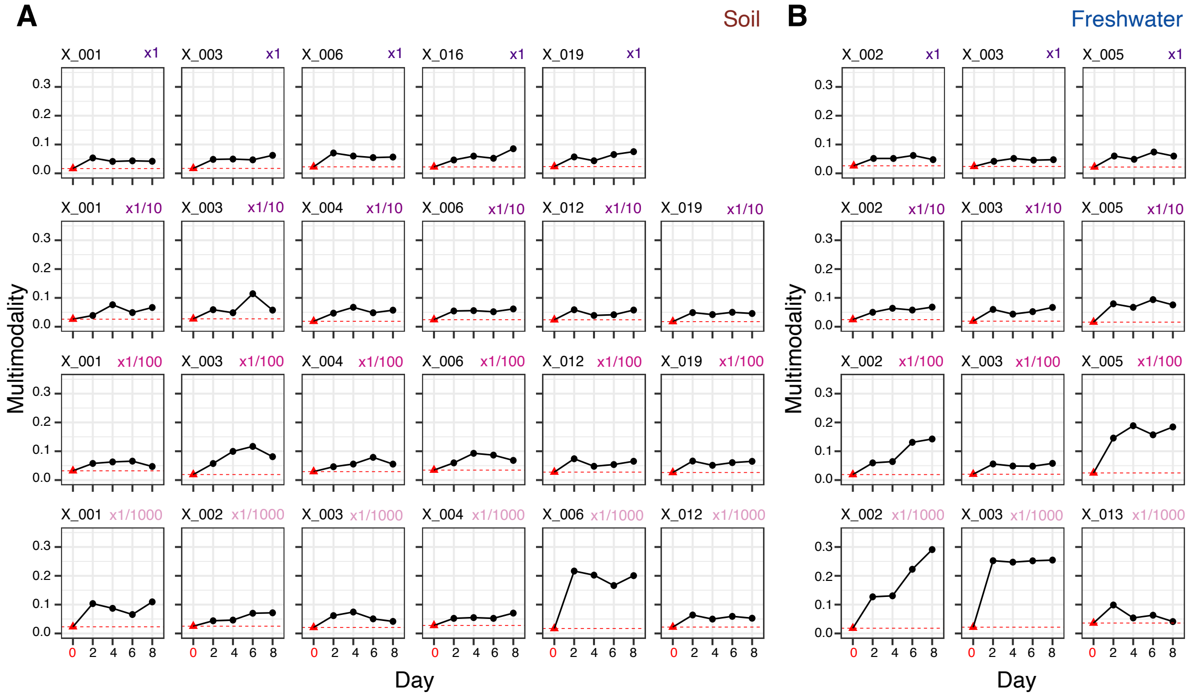

Figure S14 | Temporal changes in OTU-level multimodality. Temporal changes in multimodality for each OTU from Day 0 to Day 8 are shown. Multimodality values at Day 0 were obtained based on the multinomial-distribution simulation of founding communities (Figs. S2–3), whereas those from Day 2 to Day 8 were derived from experimental observations (Fig. 2D–E). Each panel shows results for a single OTU at a given inoculum dilution rate. The red dashed line indicates the multimodality value at Day 0. (A) Multimodality in the soil-derived inoculum experiment. (B) Multimodality in the freshwater-derived inoculum experiment.

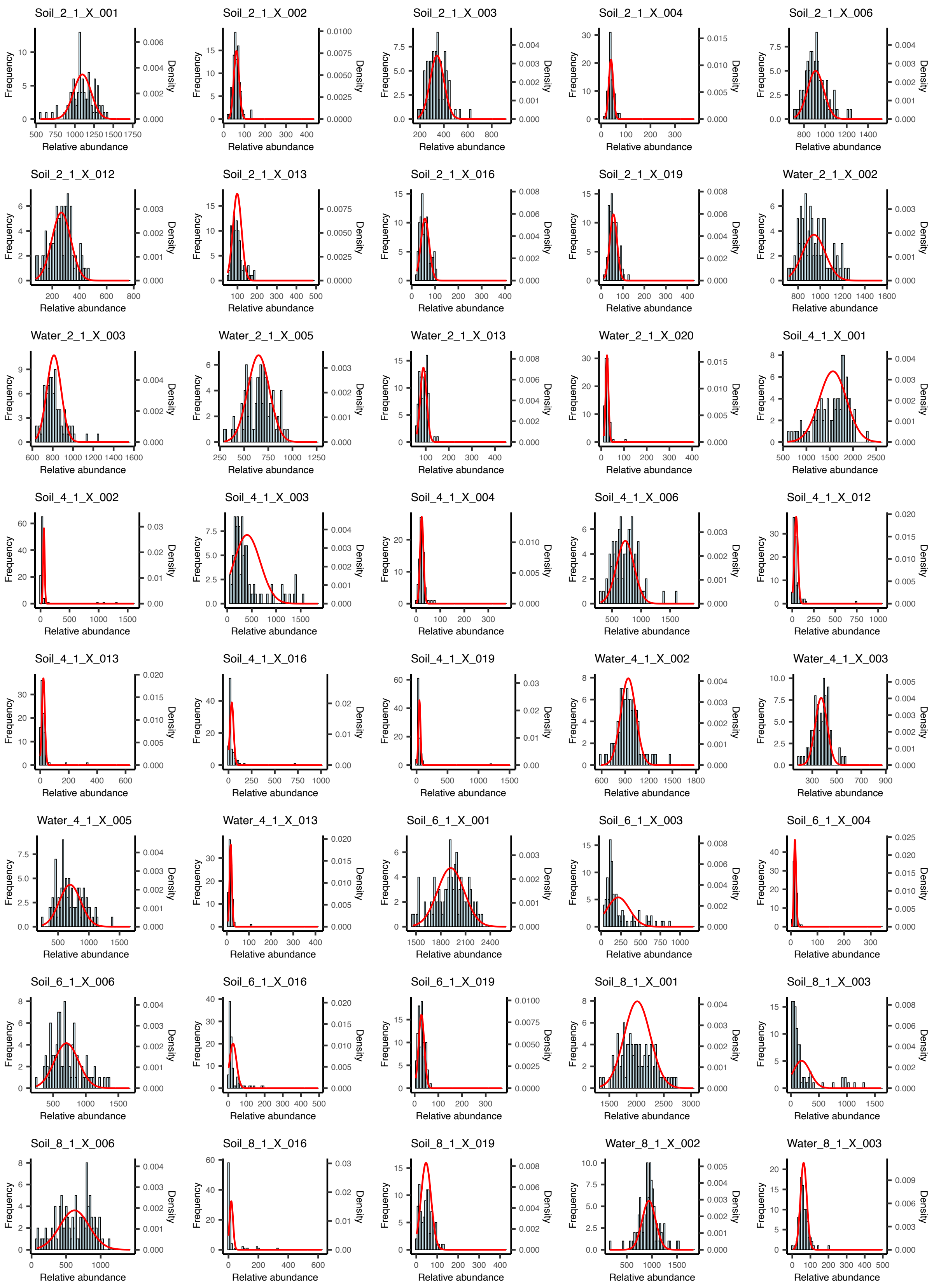
Soil_2_1_X_001
Soil_2_1_X_002
Soil_2_1_X_003
Soil_2_1_X_004
Soil_2_1_X_006
Soil_2_1_X_012
Soil_2_1_X_013
Soil_2_1_X_016
Soil_2_1_X_019
Water_2_1_X_002
Water_2_1_X_003
Water_2_1_X_005
Water_2_1_X_013
Water_2_1_X_020
Soil_4_1_X_001
Soil_4_1_X_002
Soil_4_1_X_003
Soil_4_1_X_004
Soil_4_1_X_006
Soil_4_1_X_012
Soil_4_1_X_013
Soil_4_1_X_016
Soil_4_1_X_019
Water_4_1_X_002
Water_4_1_X_003
Water_4_1_X_005
Water_4_1_X_013
Soil_6_1_X_001
Soil_6_1_X_003
Soil_6_1_X_004
Soil_6_1_X_006
Soil_6_1_X_016
Soil_6_1_X_019
Soil_8_1_X_001
Soil_8_1_X_003
Soil_8_1_X_006
Soil_8_1_X_016
Soil_8_1_X_019
Water_8_1_X_002
Water_8_1_X_003
Frequency
Density
Relative abundance

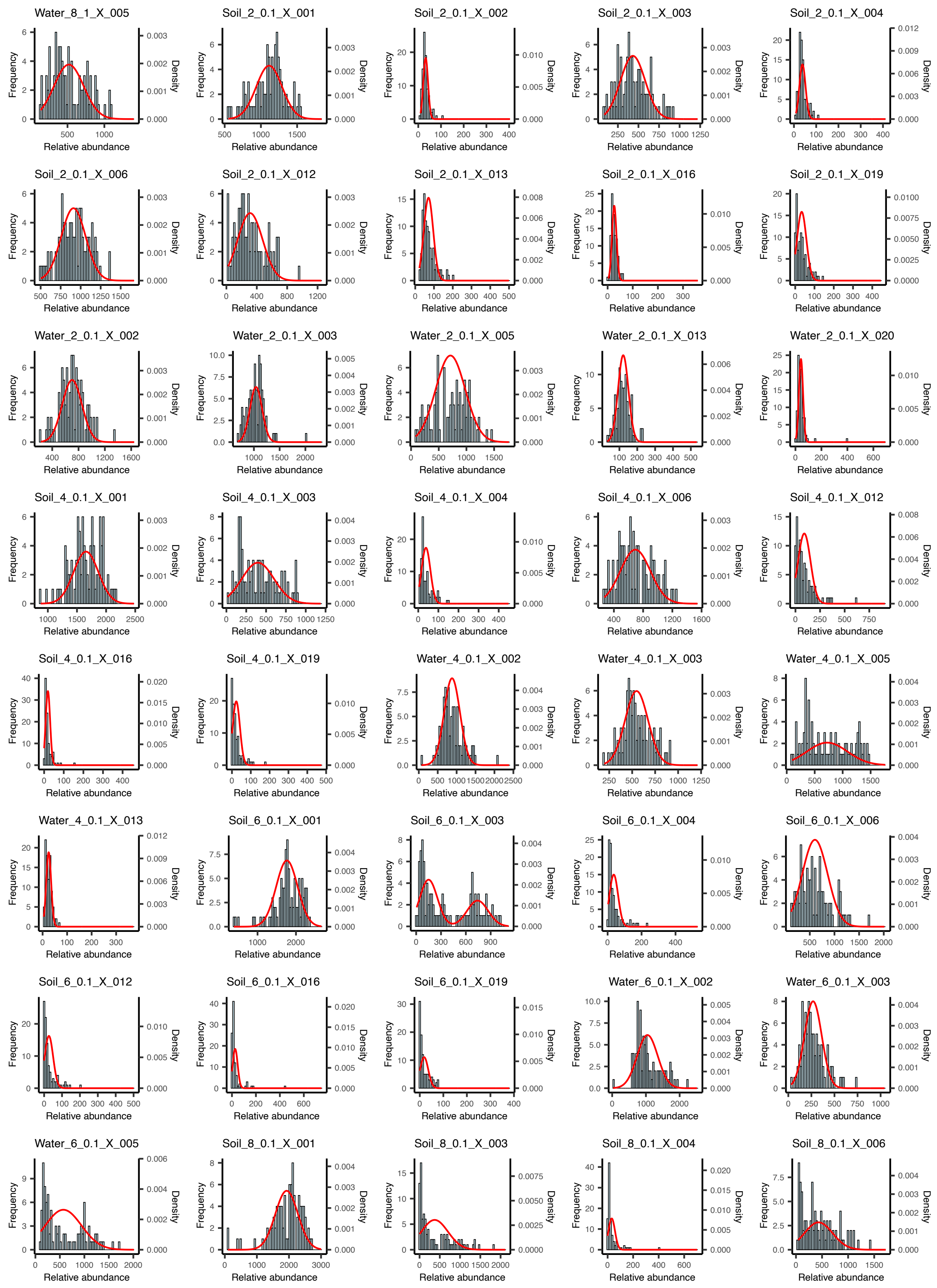
Water_8_1_X_005
Soil_2_0.1_X_001
Soil_2_0.1_X_002
Soil_2_0.1_X_003
Soil_2_0.1_X_004
Soil_2_0.1_X_006
Soil_2_0.1_X_012
Soil_2_0.1_X_013
Soil_2_0.1_X_016
Soil_2_0.1_X_019
Water_2_0.1_X_002
Water_2_0.1_X_003
Water_2_0.1_X_005
Water_2_0.1_X_013
Water_2_0.1_X_020
Soil_4_0.1_X_001
Soil_4_0.1_X_003
Soil_4_0.1_X_004
Soil_4_0.1_X_006
Soil_4_0.1_X_012
Soil_4_0.1_X_016
Soil_4_0.1_X_019
Water_4_0.1_X_002
Water_4_0.1_X_003
Water_4_0.1_X_005
Water_4_0.1_X_013
Soil_6_0.1_X_001
Soil_6_0.1_X_003
Soil_6_0.1_X_004
Soil_6_0.1_X_006
Soil_6_0.1_X_012
Soil_6_0.1_X_016
Soil_6_0.1_X_019
Water_6_0.1_X_002
Water_6_0.1_X_003
Water_6_0.1_X_005
Soil_8_0.1_X_001
Soil_8_0.1_X_003
Soil_8_0.1_X_004
Soil_8_0.1_X_006
Frequency
Density
Relative abundance

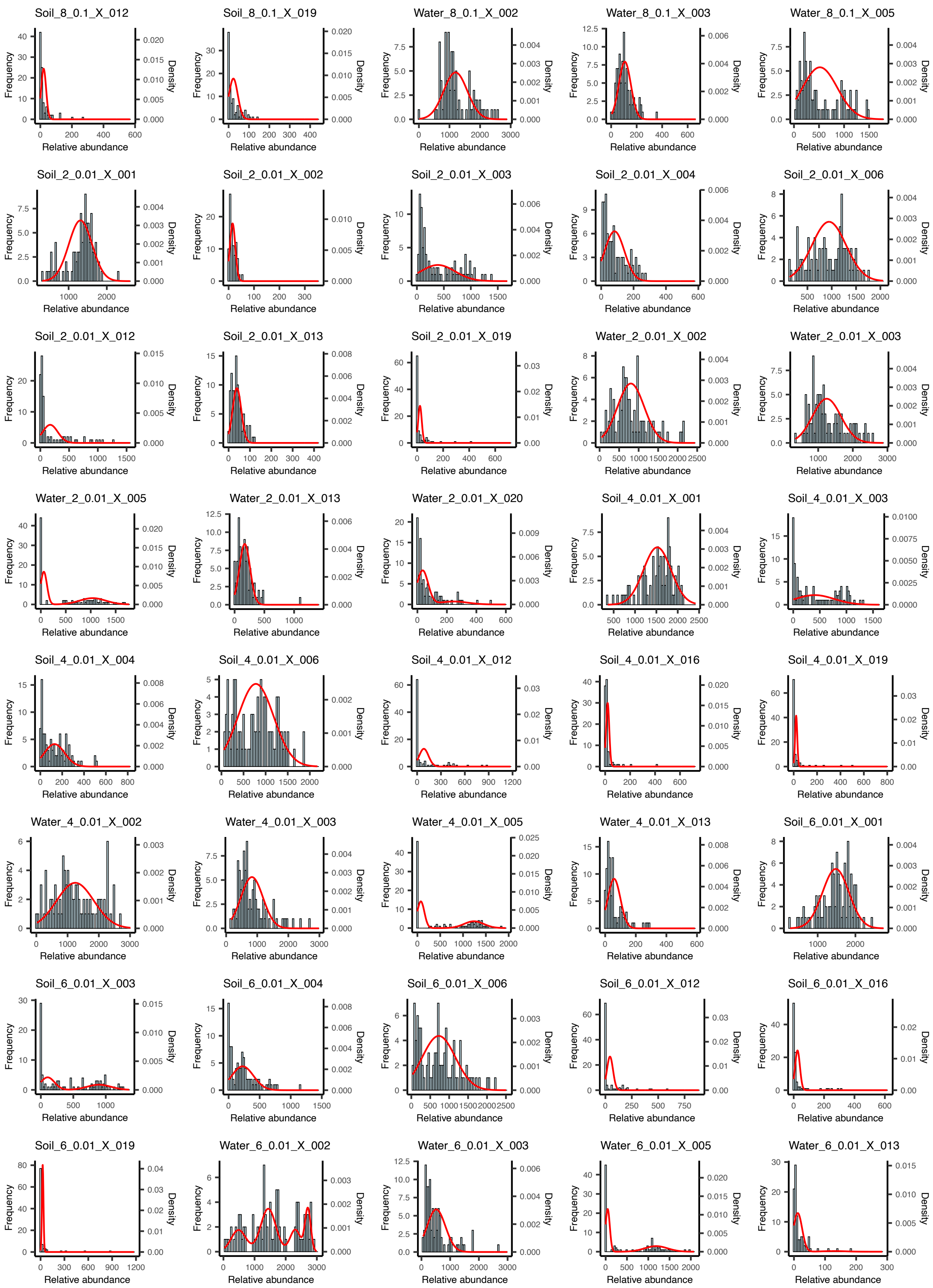
Soil_8_0.1_X_012
Soil_8_0.1_X_019
Water_8_0.1_X_002
Water_8_0.1_X_003
Water_8_0.1_X_005
Soil_2_0.01_X_001
Soil_2_0.01_X_002
Soil_2_0.01_X_003
Soil_2_0.01_X_004
Soil_2_0.01_X_006
Soil_2_0.01_X_012
Soil_2_0.01_X_013
Soil_2_0.01_X_019
Water_2_0.01_X_002
Water_2_0.01_X_003
Water_2_0.01_X_005
Water_2_0.01_X_013
Water_2_0.01_X_020
Soil_4_0.01_X_001
Soil_4_0.01_X_003
Soil_4_0.01_X_004
Soil_4_0.01_X_006
Soil_4_0.01_X_012
Soil_4_0.01_X_016
Soil_4_0.01_X_019
Water_4_0.01_X_002
Water_4_0.01_X_003
Water_4_0.01_X_005
Water_4_0.01_X_013
Soil_6_0.01_X_001
Soil_6_0.01_X_003
Soil_6_0.01_X_004
Soil_6_0.01_X_006
Soil_6_0.01_X_012
Soil_6_0.01_X_016
Soil_6_0.01_X_019
Water_6_0.01_X_002
Water_6_0.01_X_003
Water_6_0.01_X_005
Water_6_0.01_X_013
Frequency
Density
Relative abundance

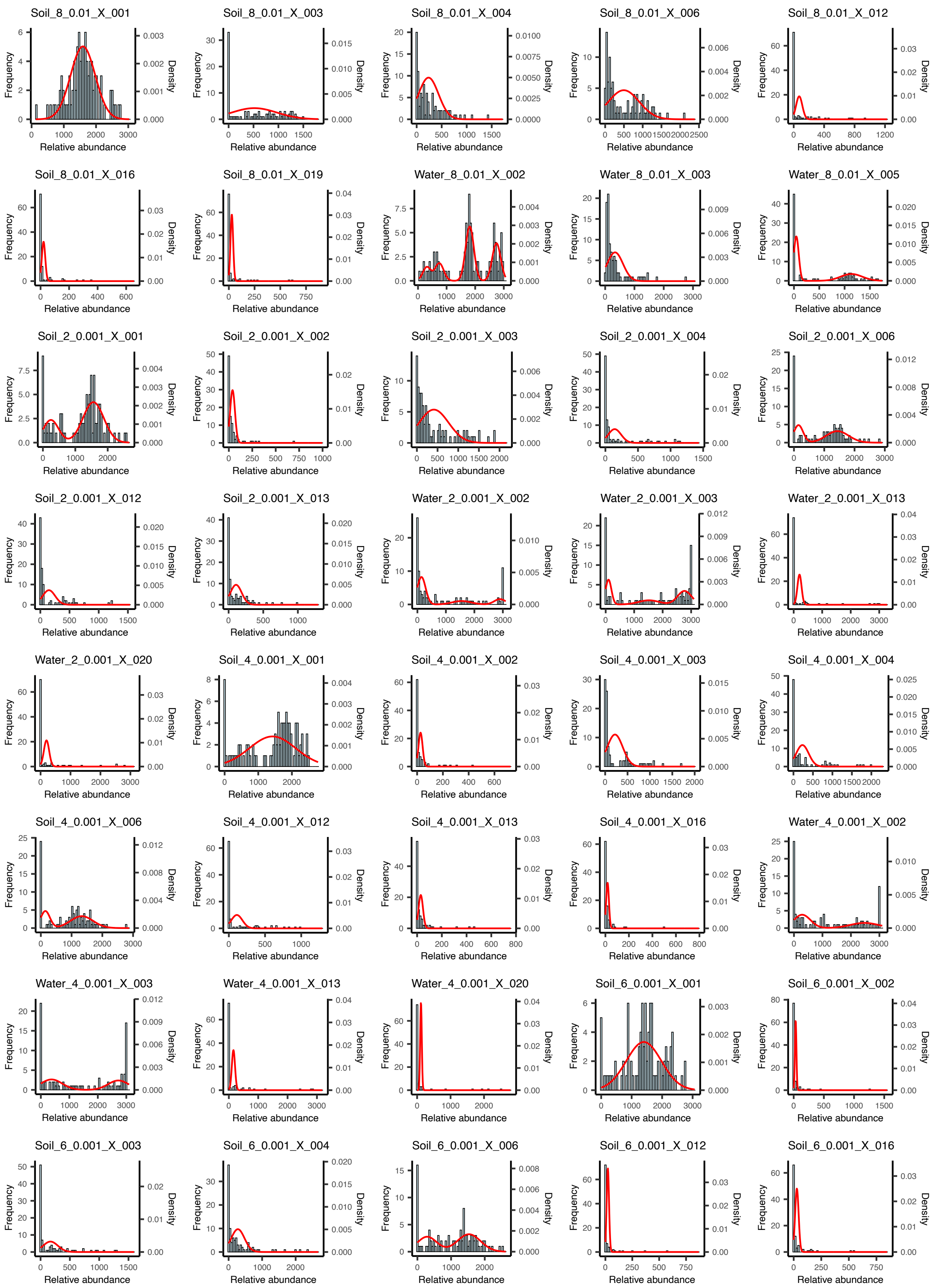
Soil_8_0.01_X_001
Soil_8_0.01_X_003
Soil_8_0.01_X_004
Soil_8_0.01_X_006
Soil_8_0.01_X_012
Soil_8_0.01_X_016
Soil_8_0.01_X_019
Water_8_0.01_X_002
Water_8_0.01_X_003
Water_8_0.01_X_005
Soil_2_0.001_X_001
Soil_2_0.001_X_002
Soil_2_0.001_X_003
Soil_2_0.001_X_004
Soil_2_0.001_X_006
Soil_2_0.001_X_012
Soil_2_0.001_X_013
Water_2_0.001_X_002
Water_2_0.001_X_003
Water_2_0.001_X_013
Water_2_0.001_X_020
Soil_4_0.001_X_001
Soil_4_0.001_X_002
Soil_4_0.001_X_003
Soil_4_0.001_X_004
Soil_4_0.001_X_006
Soil_4_0.001_X_012
Soil_4_0.001_X_013
Soil_4_0.001_X_016
Water_4_0.001_X_002
Water_4_0.001_X_003
Water_4_0.001_X_013
Water_4_0.001_X_020
Soil_6_0.001_X_001
Soil_6_0.001_X_002
Soil_6_0.001_X_003
Soil_6_0.001_X_004
Soil_6_0.001_X_006
Soil_6_0.001_X_012
Soil_6_0.001_X_016
Frequency
Density
Relative abundance

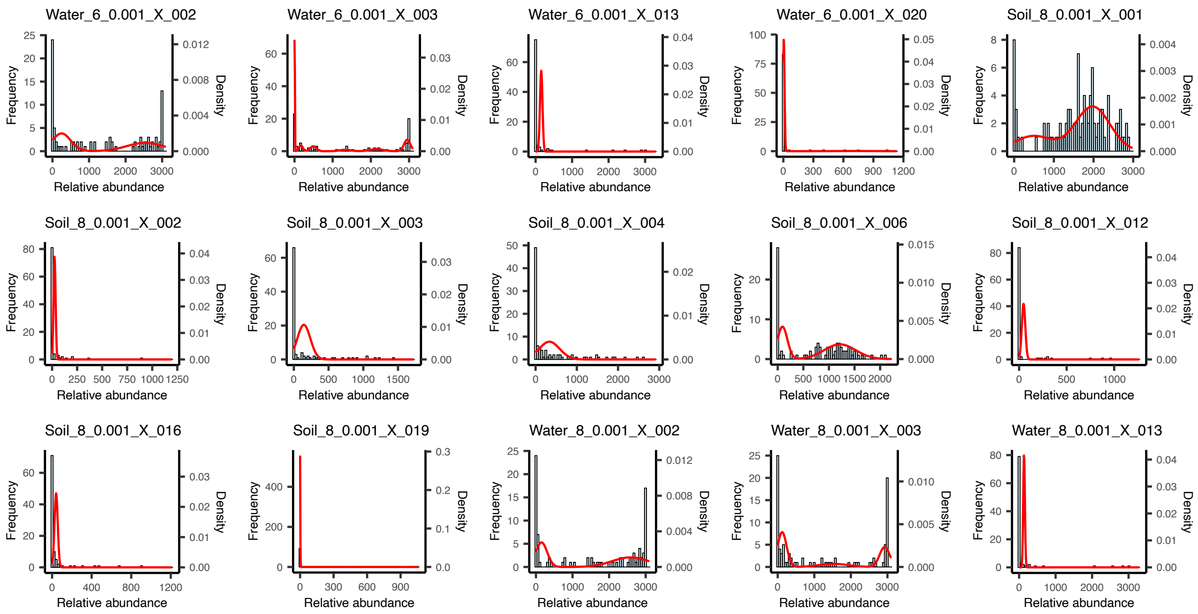

Figure S15 | Histograms of OTU abundance across replicate communities. Each histogram is overlaid with predictions from the mixture model (see Materials & Methods). The red curve in each panel represents the model's prediction. Each panel shows the abundance distribution for a specific combination of inoculum source, sampling date, dilution rate, and OTU. The combination is indicated at the top of each panel (e.g., in "Soil_2_1_X_001", the data represent a non-diluted soil-derived inoculum sampled on Day 2, with a focus on OTU X_001).

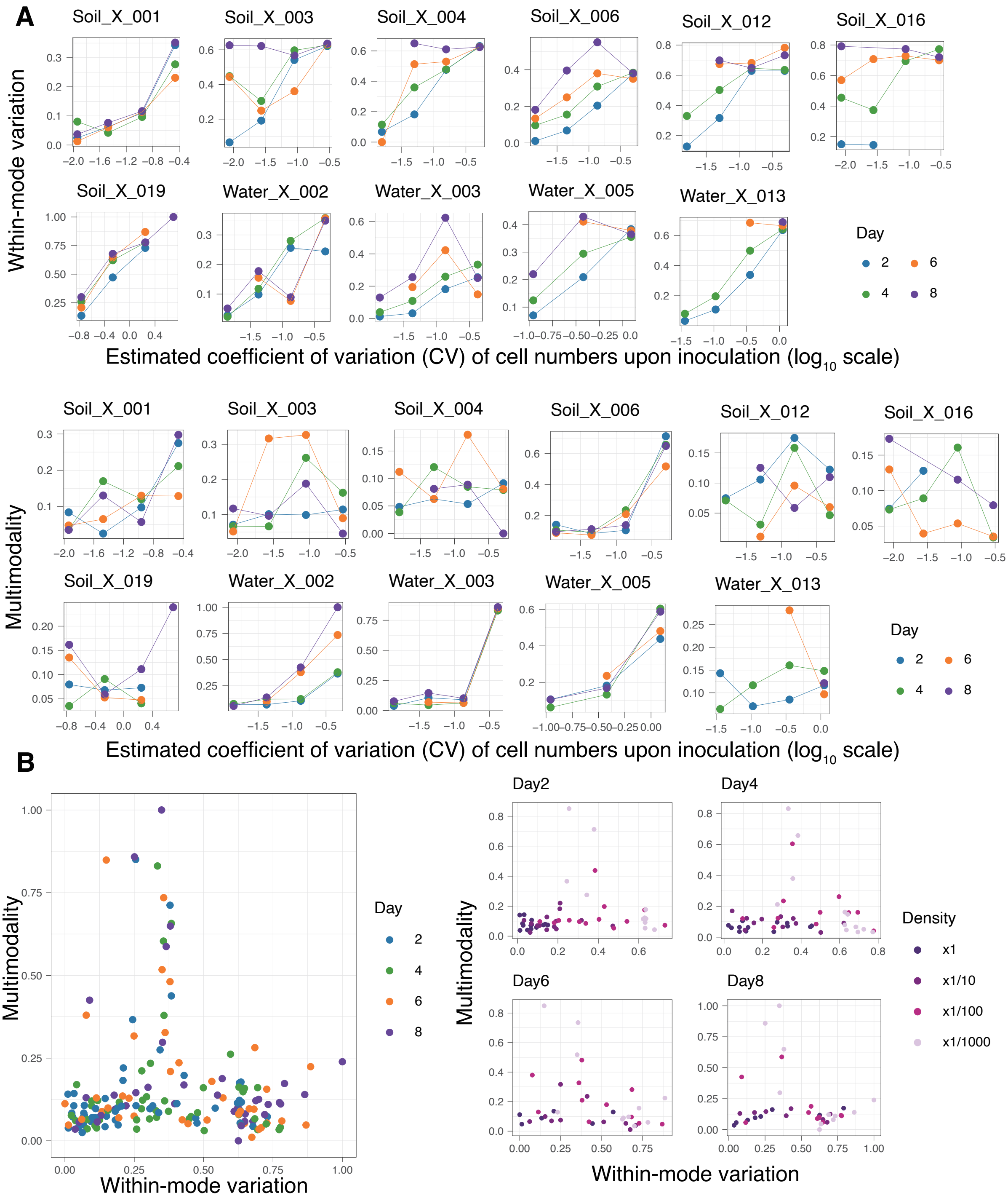

Figure S16 | Supplementary results on OTU-level multimodality and within-mode variation. (A) Temporal changes in multimodality and within-mode variation for each OTU. Only OTUs observed under at least three different initial density conditions on at least one sampling day are shown. The inoculum source and the focal OTU are indicated at the top of each panel (e.g., "Soil_X_001" denotes a soil-derived inoculum with a focus on OTU X_001). (B) Relationship between multimodality and within-mode variation. No linear relationship

between the two indices was observed. All data points are shown in the left panel, whereas inoculum dilution rates are indicated in the day-specific subpanels on the right.

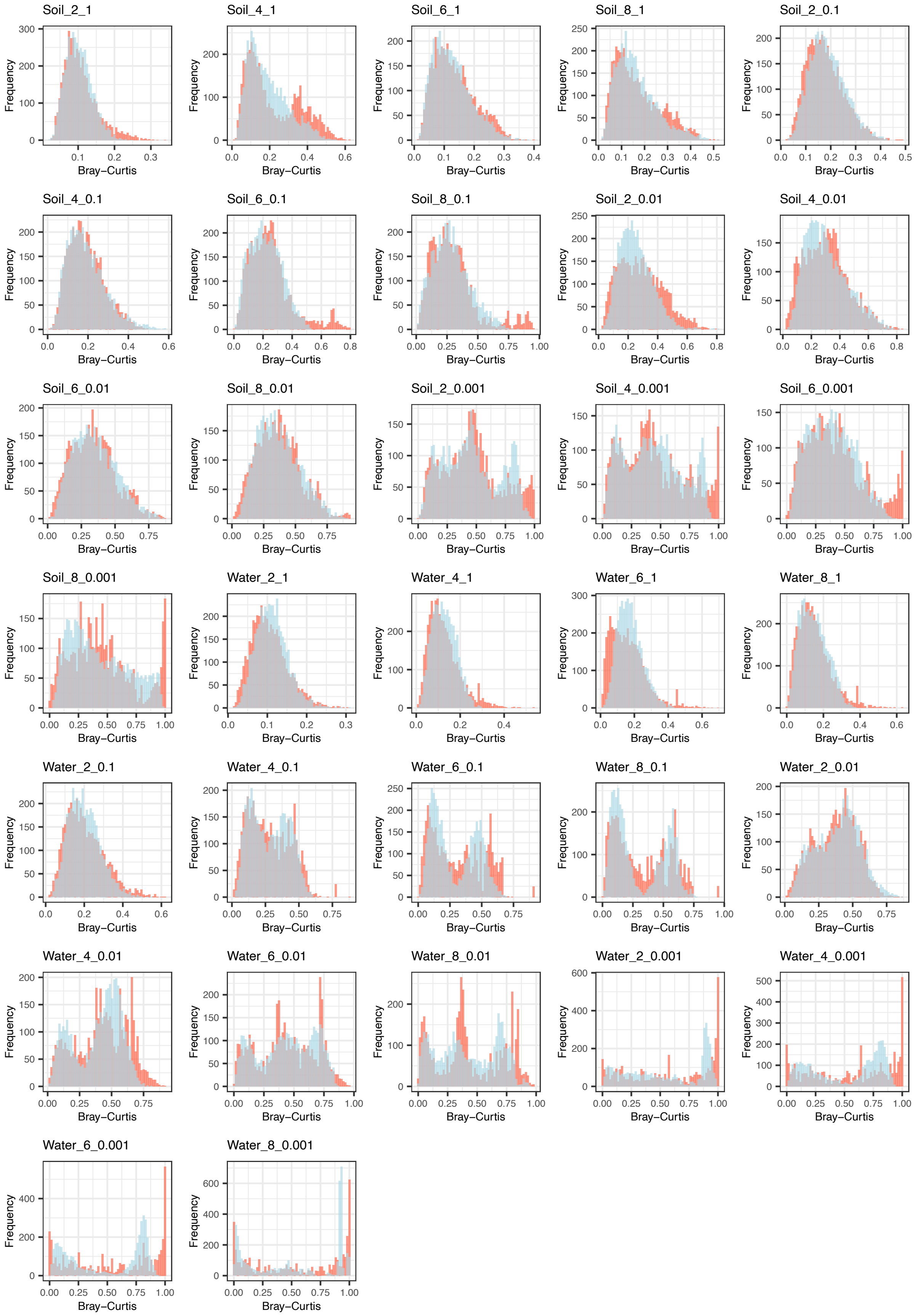

Figure S17 | Community dissimilarity histograms. Community dissimilarity histograms

overlaid with predictions from the mixture model (see Materials and Methods) are shown (these panels present the full results corresponding to Figure 3C). In each panel, the red histogram represents the empirical data, whereas the blue histogram shows the distribution predicted by the mixture model combining multinomial and normal distributions. The overlap between the empirical and simulated distributions is indicated in grey. Each panel displays the community dissimilarity distribution for a specific combination of inoculum source, sampling date, and dilution rate, as indicated at the top of each panel (e.g., “Soil_2_1” denotes a non-diluted soil-derived inoculum sampled on Day 2).

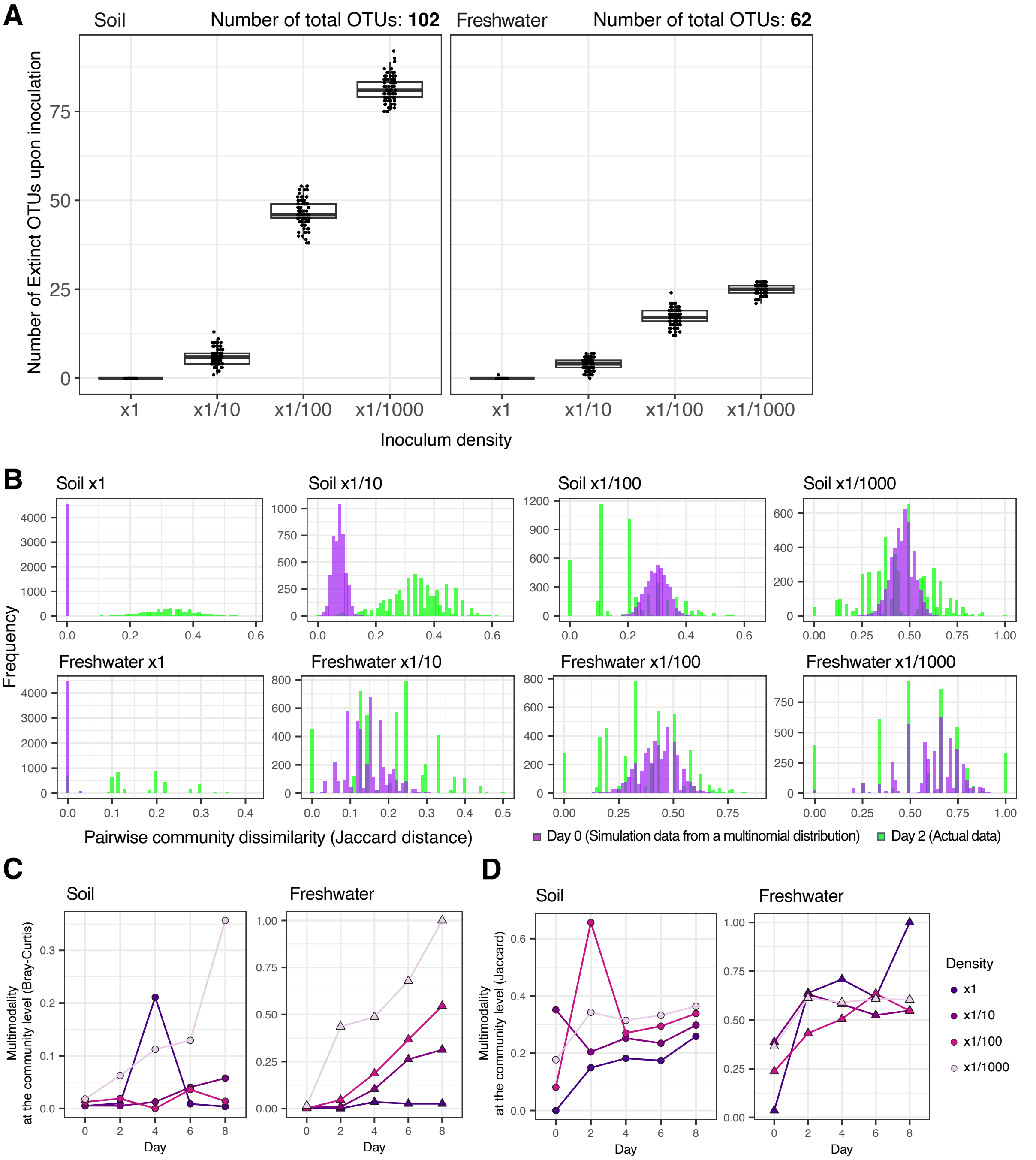

Figure S18 | Quantifying community-level divergence upon inoculation. (A) Estimated numbers of OTUs lost at inoculation. Given the number of cells introduced and the relative abundance of each OTU in the source community, the numbers of OTUs lost upon inoculation were simulated. Based on 96 draws in the multinomial simulations, boxplots of the numbers of "extinct" OTUs are shown at each dilution rate for each source community. (B) Distributions of pairwise Jaccard dissimilarity among communities. The distribution of pairwise Jaccard dissimilarities among 96 simulated replicate communities at inoculation is

shown as purple histograms. Green histograms show the distributions of pairwise Jaccard distances among experimentally observed communities at Day 2. (C) Temporal changes in multimodality calculated using Bray–Curtis dissimilarity. This panel extends Figure 3D by adding estimated multimodality values on Day 0 derived from simulated data. Multimodality values are based on Bray–Curtis dissimilarity, and all other legend details follow Figure 3D. Data on Day 0 were obtained from simulations, whereas data from Day 2 to Day 8 were obtained from experimental observations. (D) Temporal changes in multimodality calculated using Jaccard dissimilarity.

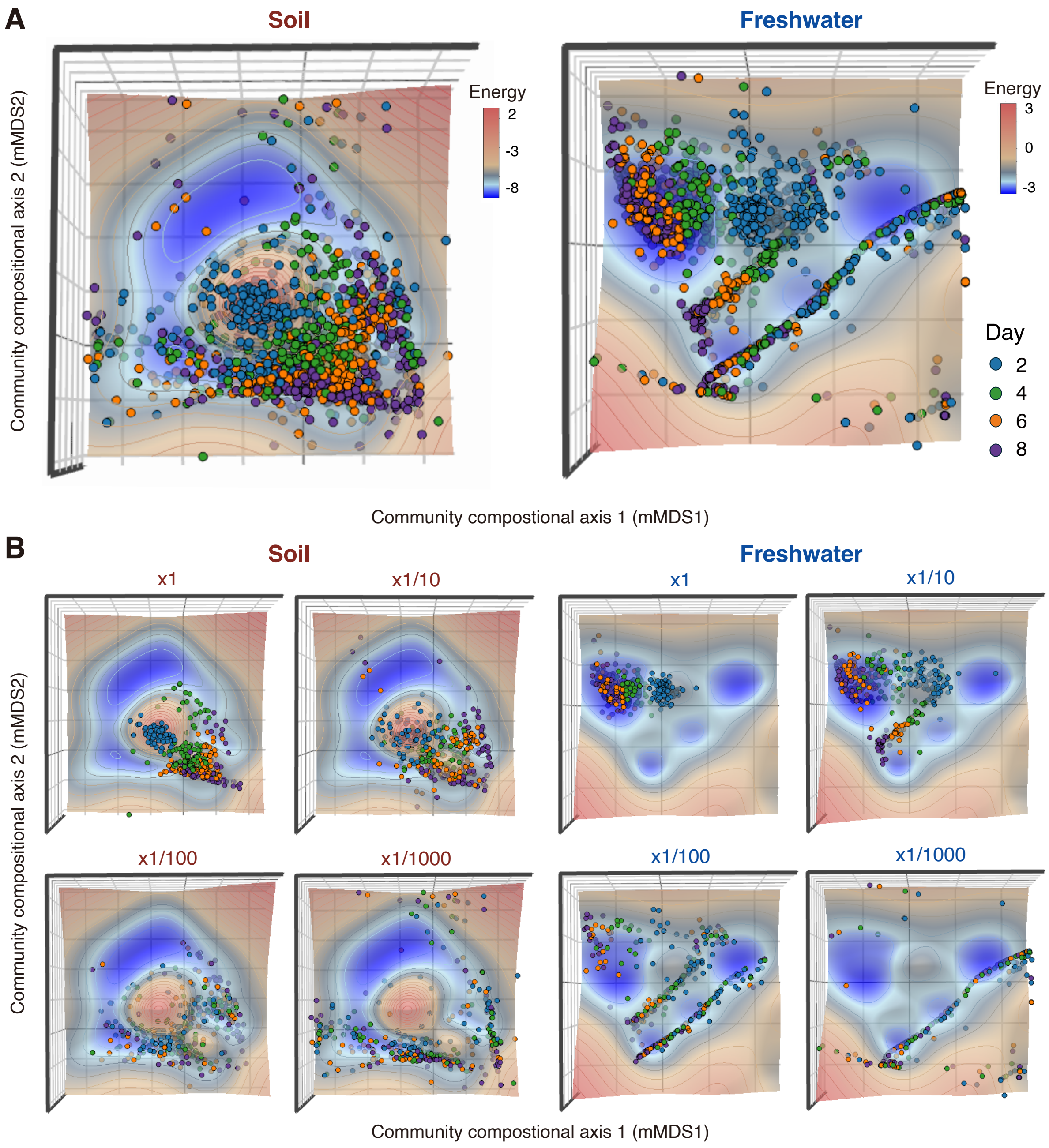

Figure S19 | Top-down view of the energy landscapes (Framework 1). The legend follows that of Figure 4A–B.

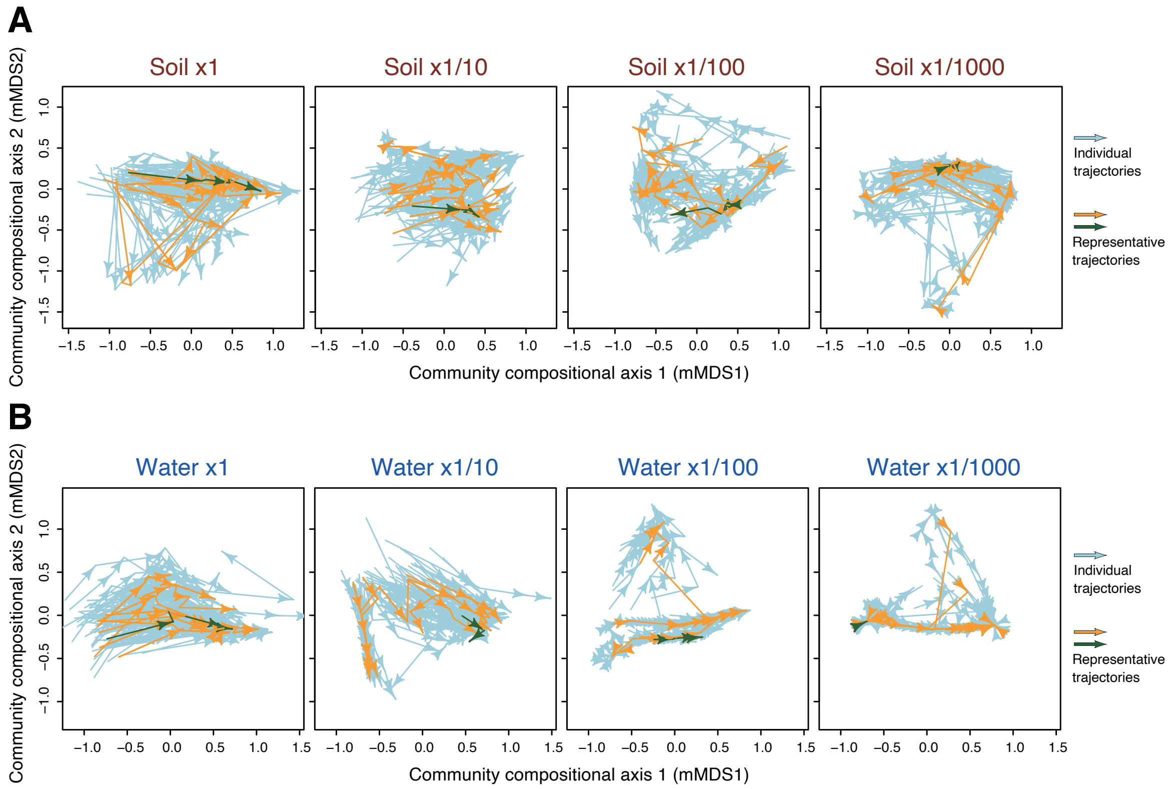

Figure S20 | Ecological dynamic regime plots with aligned axis scales. Identical scales are used for the mMDS axes. The legend follows that of Fig. 5.

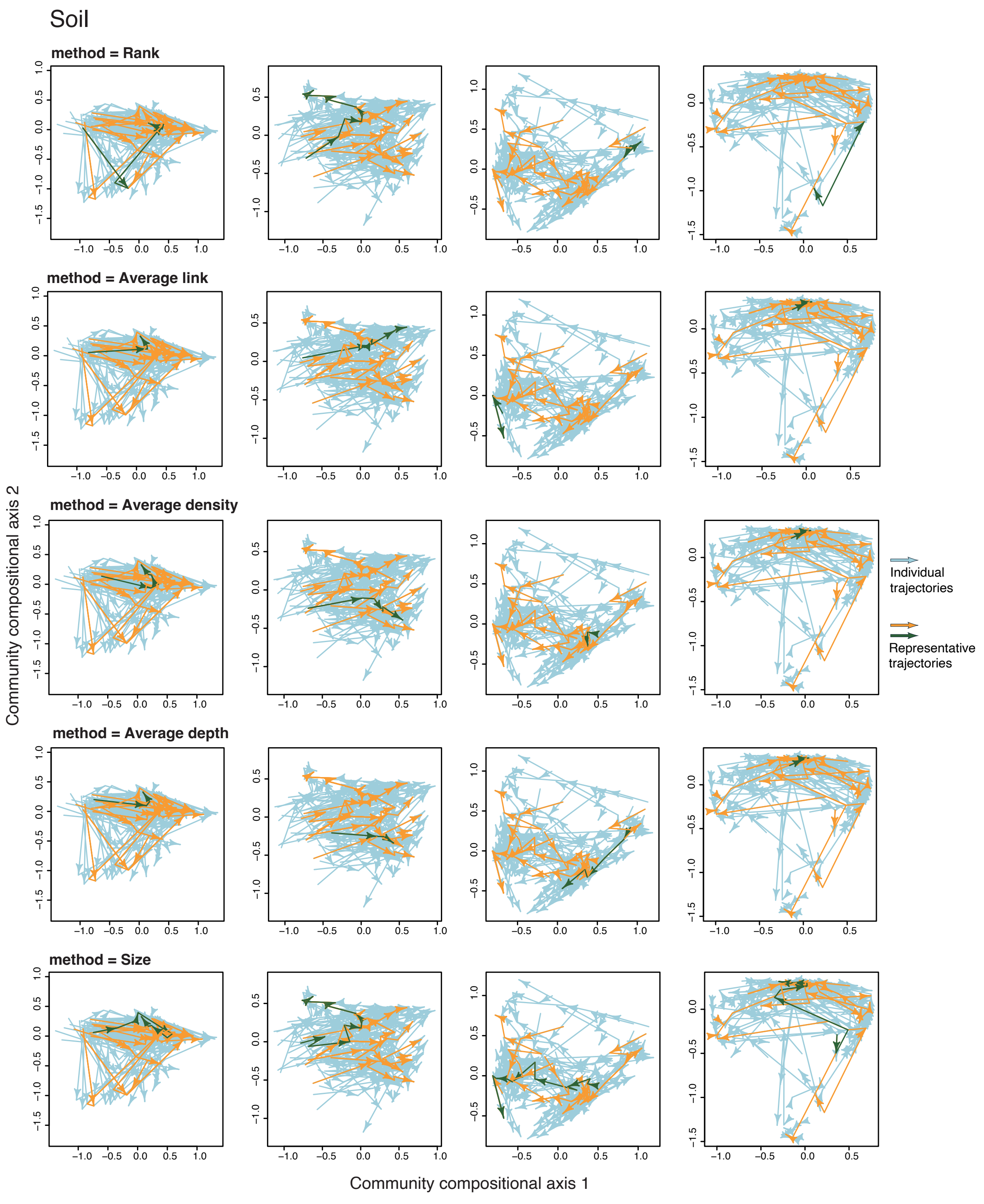

Figure S21 | Criteria for selecting the representative trajectories of ecological dynamic regimes (soil-derived microbiomes). The representative trajectories (green arrows) selected with each of the five criteria are shown in the mMDS plot of soil-derived communities were used to calculate the dynamic dispersion (dDis) of each dynamic regime. The result of the method using the average depth is also shown in Figure 5. The method used to select the representative trajectory is indicated at the top of each panel. Panels are arranged horizontally by dilution rate.

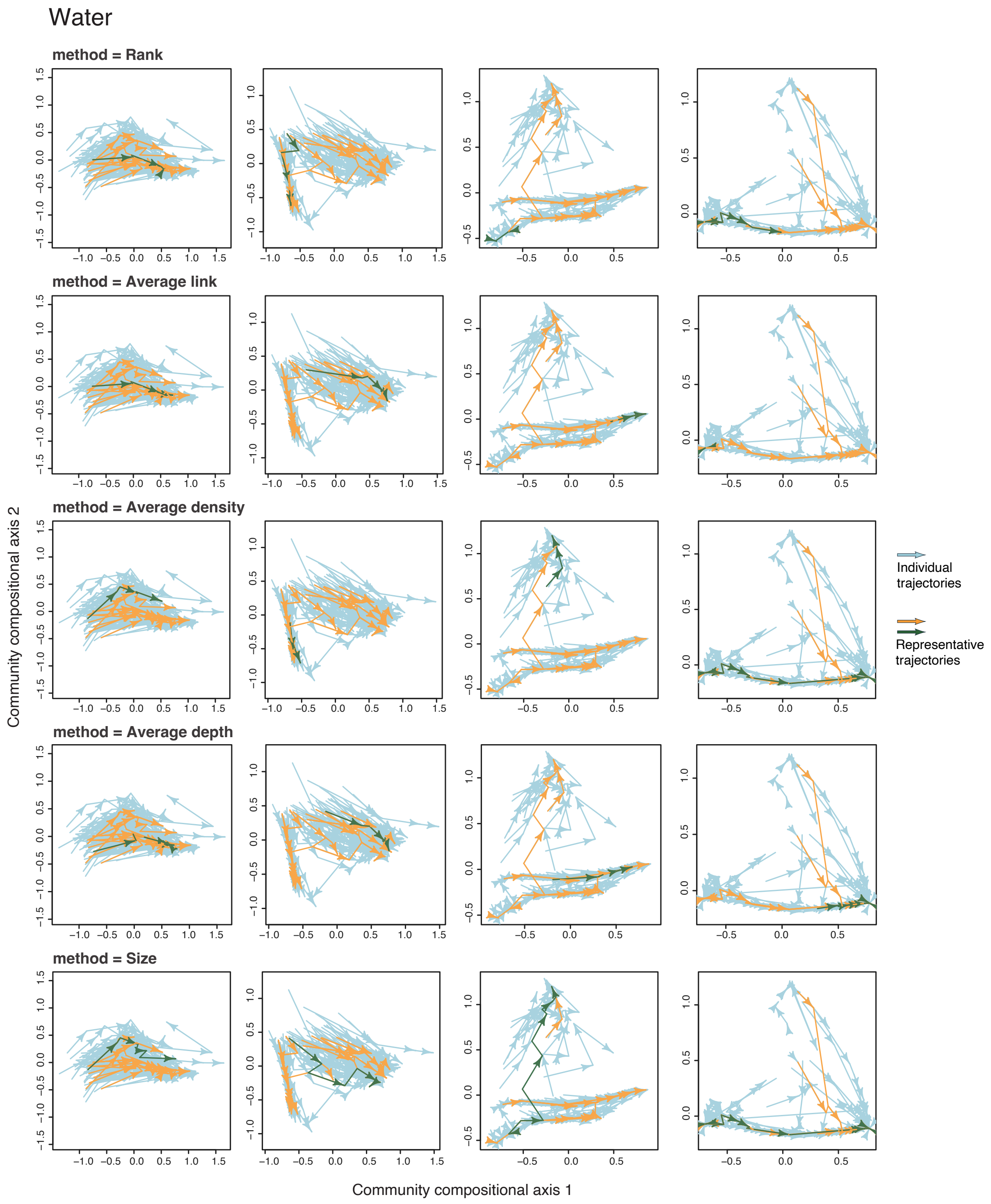

604

605 **Figure S22 |** Criteria for selecting representative trajectories of ecological dynamic regimes
606 (freshwater-derived microbiomes). The representative trajectories (green arrows) selected
607 with each of the five criteria are shown in the mMDS plot of freshwater-derived communities
608 were used to calculate the dynamic dispersion (dDis) of each dynamic regime. The result of
609 the method using the average depth is also shown in Figure 5. The method used to select the
610 representative trajectory is indicated at the top of each panel. Panels are arranged horizontally
611 by dilution rate.

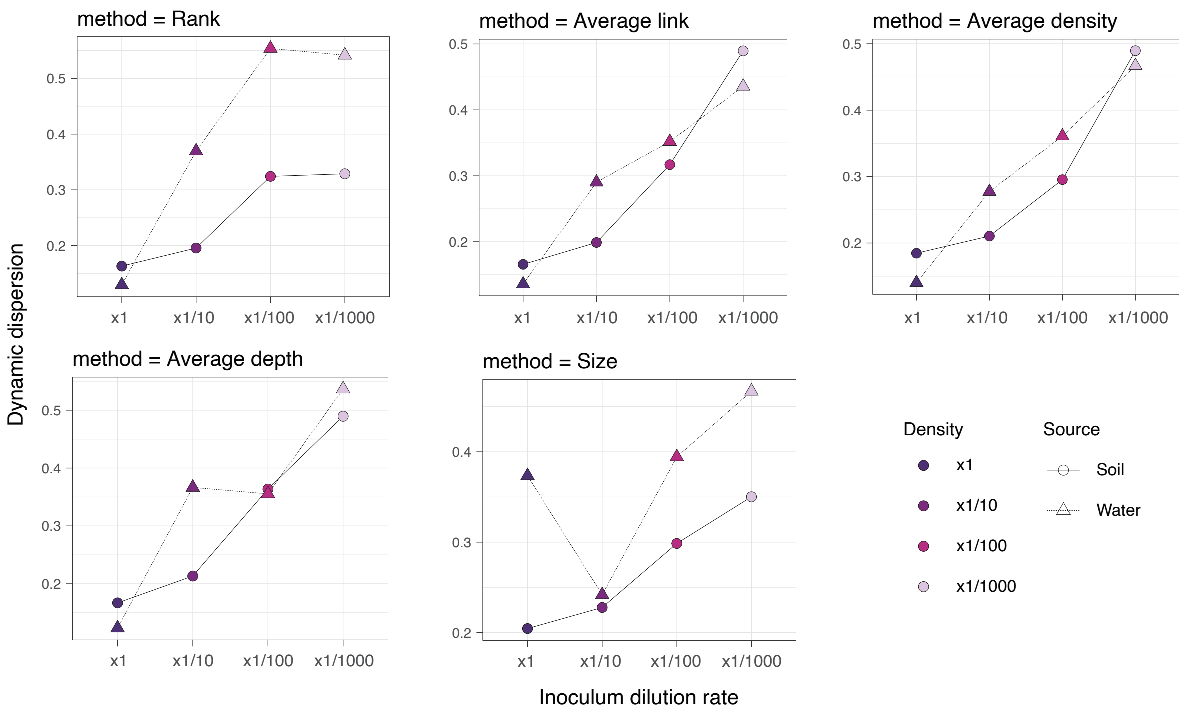

Figure S23 | Dynamic dispersion metrics based on alternative criteria. In each panel, a representative trajectory selected by each method was used in the calculation of the dynamic dispersion. Except when using the size as the selection criterion, consistent patterns of increasing dynamic dispersion values with increasing dilution rates was observed.

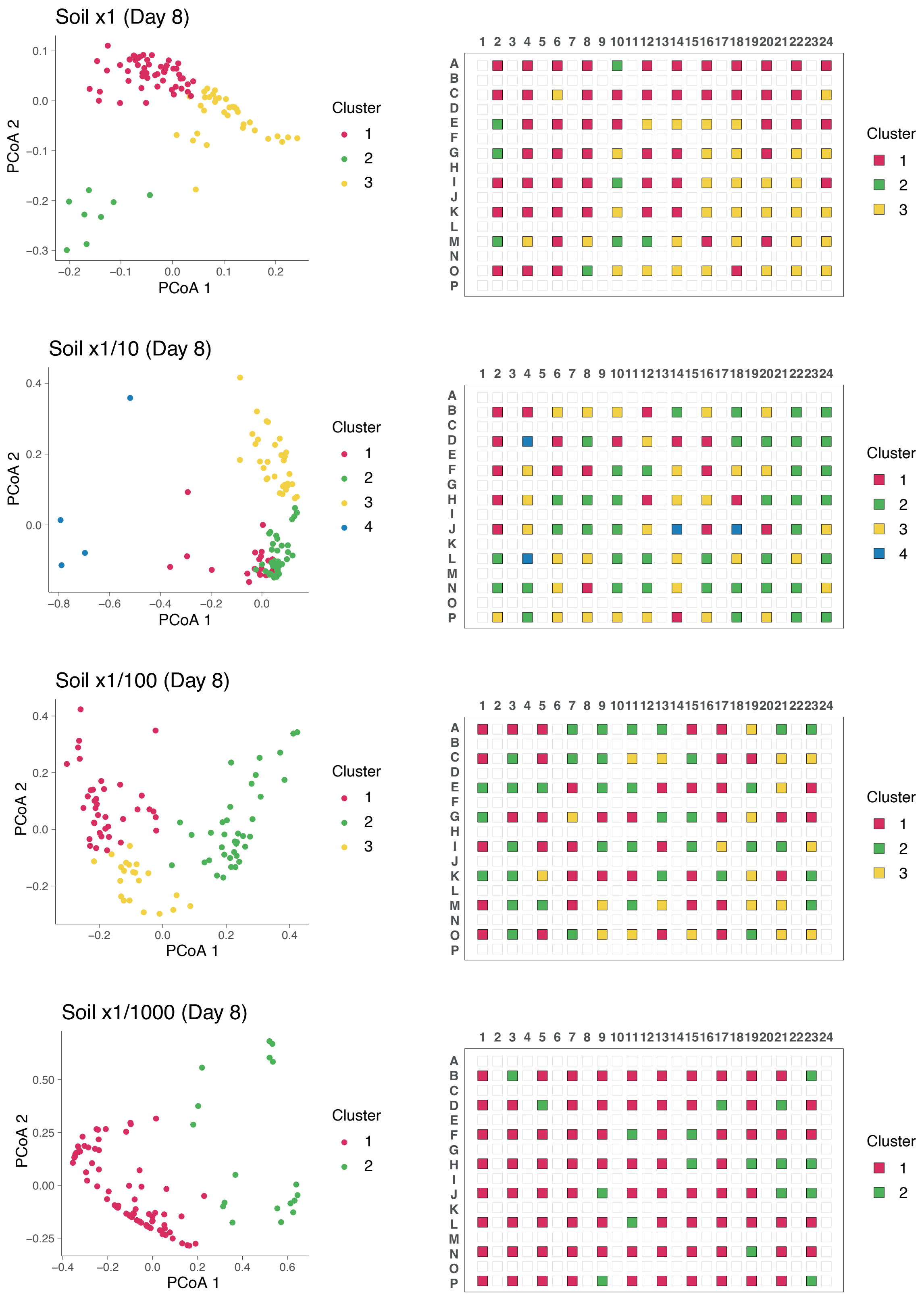

Figure S24 | Spatial distributions of community structure within the culture plate (soil-derived communities). To assess whether the spatial arrangement of wells within the plate

affects community structure, we conducted a clustering analysis of replicate communities. For each dilution rate of the soil-derived microbiome, we applied *k*-means clustering followed by silhouette coefficient analysis to determine the optimal number of clusters. After selecting the number of clusters *k*, we used principal coordinate analysis (PCoA) to reduce the dimensionality of community composition data and visualized the results, with colors representing different clusters. The spatial positions of replicate communities assigned to each cluster are shown on the right panels indicating the spatial organization within the culture plate.

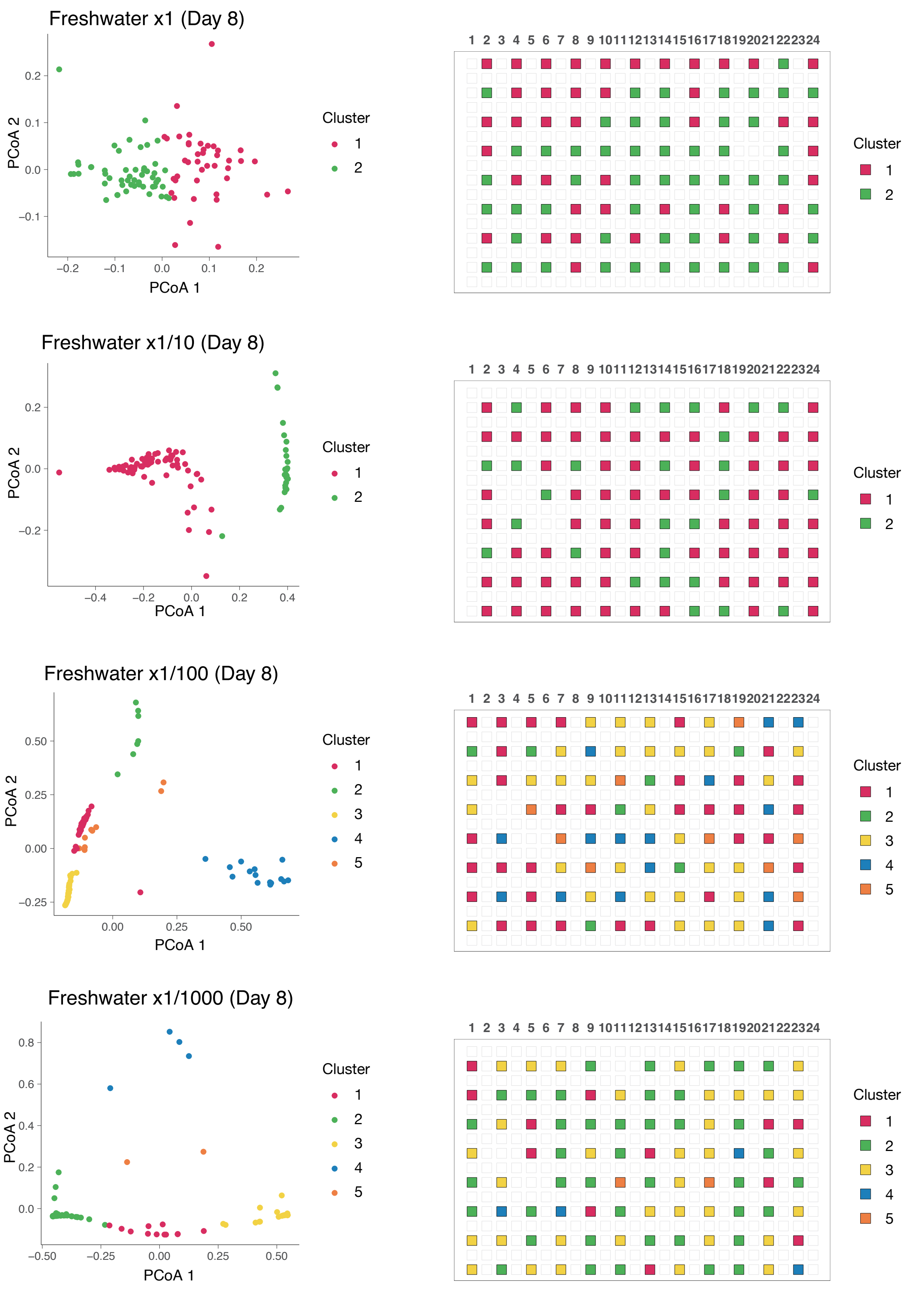

Figure S25 | Spatial distributions of community structure within the culture plate (freshwater-derived communities). To assess whether the spatial arrangement of wells within the plate

affects community structure, we conducted a clustering analysis of replicate communities. For each dilution rate of the freshwater-derived microbiome, we applied *k*-means clustering followed by silhouette coefficient analysis to determine the optimal number of clusters. After selecting the number of clusters *k*, we used principal coordinate analysis (PCoA) to reduce the dimensionality of community composition data and visualized the results, with colors representing different clusters. The spatial positions of replicate communities assigned to each cluster are shown on the right panels indicating the spatial organization within the culture plate.

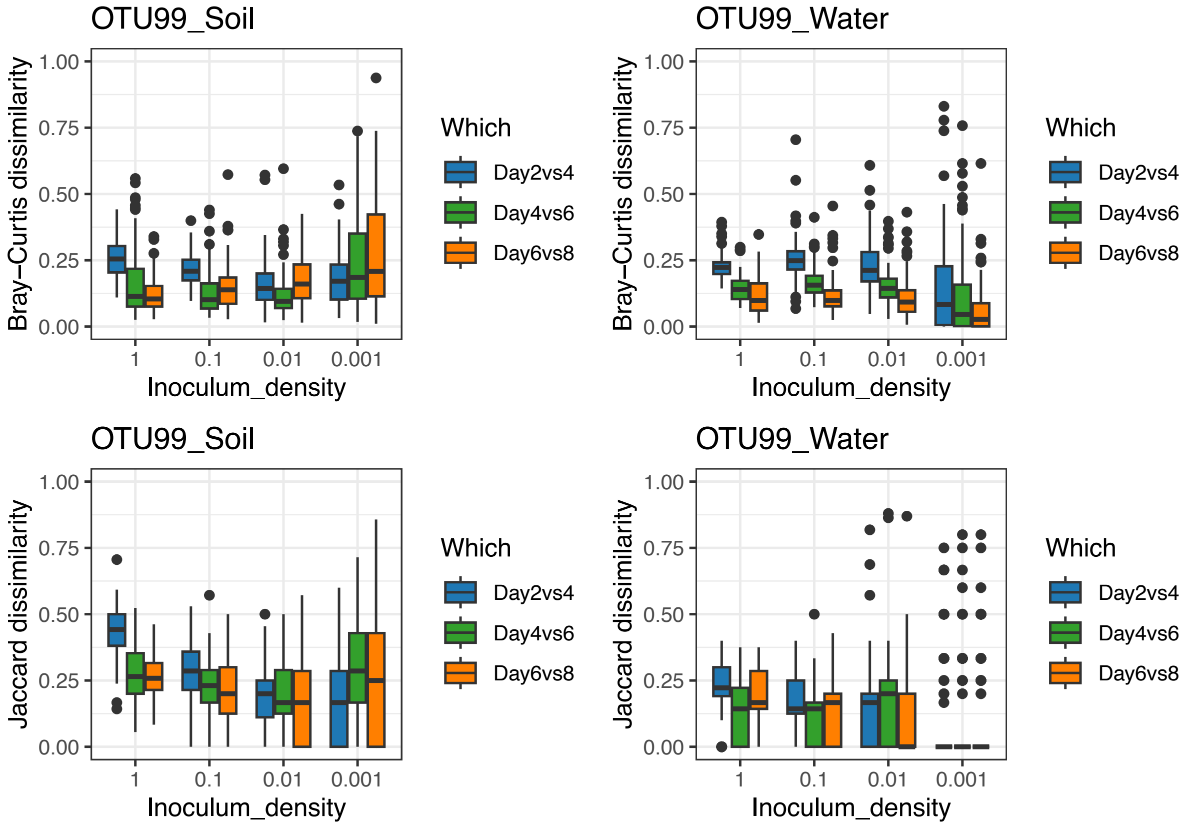

Figure S26 | Temporal changes in community composition. Community compositional dissimilarity between consecutive time points is shown for each source community at each dilution rate. Results based on Bray–Curtis and Jaccard dissimilarities are shown in the upper and lower panels, respectively.
